# Systematic inference and comparison of multi-scale chromatin sub-compartments connects spatial organization to cell phenotypes

**DOI:** 10.1038/s41467-021-22666-3

**Published:** 2021-05-10

**Authors:** Yuanlong Liu, Luca Nanni, Stephanie Sungalee, Marie Zufferey, Daniele Tavernari, Marco Mina, Stefano Ceri, Elisa Oricchio, Giovanni Ciriello

**Affiliations:** 1 Department of Computational Biology, University of Lausanne, Lausanne, Switzerland; 2 Swiss Cancer Center Leman, Lausanne, Switzerland; 3 Swiss Institute of Bioinformatics, Lausanne, Switzerland; 4 Department of Electronics, Information, and Bioengineering, Politecnico di Milano, Milan, Italy; 5 Swiss Institute for Experimental Cancer Research (ISREC) School of Life Sciences, EPFL, Épalinges, Switzerland

**Keywords:** Computational biology and bioinformatics, Epigenetics

## Abstract

Chromatin compartmentalization reflects biological activity. However, inference of chromatin sub-compartments and compartment domains from chromosome conformation capture (Hi-C) experiments is limited by data resolution. As a result, these have been characterized only in a few cell types and systematic comparisons across multiple tissues and conditions are missing. Here, we present Calder, an algorithmic approach that enables the identification of multi-scale sub-compartments at variable data resolution. Calder allows to infer and compare chromatin sub-compartments and compartment domains in >100 cell lines. Our results reveal sub-compartments enriched for poised chromatin states and undergoing spatial repositioning during lineage differentiation and oncogenic transformation.

## Abstract (web summary)

Computational algorithms to infer chromatin sub-compartments and compartment domains require high-resolution Hi-C maps. Here the authors present Calder, an algorithm that can infer sub-compartments and compartment domains with variable resolution Hi-C data, and they apply it to more than a hundred Hi-C experiments to study sub-compartment repositioning.

## Introduction

In interphase, the chromatin is packaged into a hierarchy of three-dimensional (3D) structural elements (SEs) emerging from interactions and insulation of distinct DNA regions^1–3^. Hi-C technologies have allowed to quantify and computationally model such interactions to unravel chromatin spatial organization^4–6^. The formation of chromatin SEs is driven by two major mechanisms: loop extrusion of chromatin fibers mediated by CTCF and cohesin, and chromatin compartmentalization, which segregates chromatin regions with different patterns of histone acetylation and methylation^7^. Loop extrusion has been associated with the formation of topologically associating domains (TADs) and structural loops^8–10^, whereas chromatin compartmentalization segregates the chromatin into spatial compartments^11,12^ and compartment domains^10^. At a broad scale, chromatin segregates into two major compartments, one preferentially localized at the core of the nucleus and exhibiting high transcriptional activity (A compartment), and another localized closer to the nuclear lamina and enriched for repressed and gene-depleted chromatin (B compartment). Within each chromosome, DNA regions belonging to a given compartment are defined as compartment domains. Interestingly, recent experiments based on CTCF and cohesin depletion have shown that although often overlapping and sometime coincident, TADs and compartment domains are in fact distinct SEs^13^. Compartment domains from the same compartment preferentially interact among each other and, in Hi-C contact correlation maps, the alternance of A and B compartment domains generates a chessboard or “plaid” pattern reflecting enrichment or depletion of Hi-C interactions^4^.

Computational inference of A and B compartments has been performed across multiple cell types and it showed, for example, that compartments are less conserved across cell types than TADs^5^ and phenotypic changes are more frequently associated with compartment repositioning of a given genomic region than with structural disruption of loops or TADs^14^. Importantly, A and B compartments have been shown enriched for distinct histone modifications, which are consistent with the observed transcriptional activity. However, chromatin activity encompasses multiple states^15^, some of which can only be captured through a more refined subcompartmentalization^2^. It was previously proposed that the A and B dichotomy might not be sufficient to explain chromatin compartmentalization^16^. Indeed, up to 6 subcompartments have been inferred by clustering interchromosomal interactions using a Gaussian Hidden Markov Model (GHMM)^2,10^ and, recently, an intermediate compartment was characterized using Hi-C and imaging techniques in colorectal tumor samples^17^. Computational inference of subcompartments has so far relied on interchromosomal contacts and, thus, it has been possible only for high-resolution experiments. For example, the GHMM approach was exclusively applied to the GM12878 cell line (4.9 billion read pairs). Machine learning-based imputation of Hi-C contacts has been used to enhance data resolution and allow subcompartment inference in eight additional Hi-C experiments^18^. However, as we will show, this approach frequently fails to correctly infer subcompartments when challenged with relatively low-resolution experiments or when patterns of interchromosomal interactions deviate significantly from the training dataset. As a consequence, the identification of chromatin subcompartments and compartment domains remains unfeasible for the vast majority of available Hi-C datasets. Here, we propose an algorithm able to infer compartment domains and hierarchies of subcompartments in Hi-C contact maps with highly variable total number of reads. By inferring chromatin subcompartments across multiple cell types and states, we could study repositioning of compartment domains and its association with cell phenotypes.

## Results

### The Calder algorithm

**Fig. 1:**
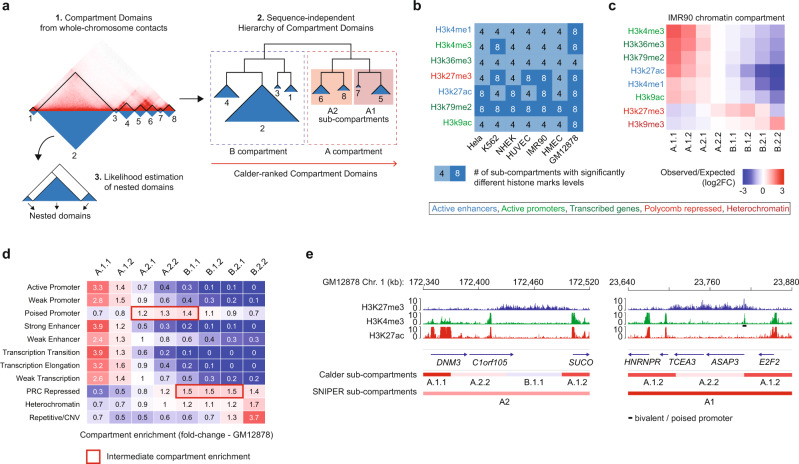
Inference and characterization of chromatin subcompartments using Calder. **a** Main steps of Calder: Step 1) identifying compartment domains from whole-chromosome contacts (top left); Step 2) deriving sequence-independent hierarchy of compartment domains (right); Step 3) finding nested domains from short-range contacts (bottom left). **b** Maximum number of subcompartments among which significant differences between mean ChiP-seq intensities of the domains were found for different histone marks (rows) and cell lines (columns). **c** Enrichment of histone marks (rows) in each subcompartment (columns) of IMR90 cell line. Log_2_ fold-changes between the median value within a compartment and the expected median value is color coded. Histone modification names are color coded based on the regulatory element they mark. **d** Enrichment of ChromHMM states (rows) in each subcompartment (columns) for GM12878. Ratios between the distribution of compartment labels for each ChromHMM state and their expected distribution is color coded and reported. **e** Representative examples of subcompartments inferred by Calder and SNIPER (colored tracks at the bottom) in two regions of Chr.1. of the GM12878 cell line. ChIP-seq tracks for H3K27me3 (blue), H3K4me3 (green), and H3K27ac (red) are shown in these regions. The bivalent/poised promoter (marked by both H3K4me3 and H3K27me3) of the *ASAP3* gene is marked.

We introduce an algorithmic approach that infers a complete hierarchy of compartment domains using exclusively intrachromosomal interactions, which are more frequent than interchromosomal ones and thus alleviate the requirements on data resolution (Fig. 1a). Our approach consists of two main steps plus an optional one. First, it computes whole-chromosome contact similarities among genomic loci (Fisher’s z-transformed correlations) and identifies compartment domains by segmenting each chromosome into regions having high intraregion similarity and low inter-region similarity. Next, compartment domains are clustered using a divisive hierarchical clustering approach exclusively based on their interdomain contacts (3D-proximity) and ignoring their contiguity along the genome sequence (1D-proximity). Dendrograms generated for each chromosome are internally reordered, without disrupting the clustering structure, to match subcompartments among different chromosomes. Finally, a mixture log-normal distribution model can be applied to short-range contacts within each compartment domain to estimate the likelihood of nested subdomains (see Methods for an in-depth description of the algorithm). The resulting compartment domain hierarchy describes how compartment domains group to progressively form subcompartments at various scales. As such, subcompartments can be explored at multiple levels of granularity, as each internal node of the dendrogram can in principle be thought as a subcompartment comprising all the domains descending from it. For analytical purposes, compartment domains within each chromosome are assigned a normalized rank between 0 and 1, which identifies their position in the dendrogram (0 being the most inactive and 1 the most active compartment domain). Importantly, the position of a compartment domain in the dendrogram can vary across cell models since it depends exclusively on 3D-proximity with other domains. This is in stark contrast to previous approaches inferring TAD hierarchies^19–21^, which invariably preserved TAD 1D-proximity, i.e., two contiguous TADs along the genome sequence will also be contiguous in the inferred hierarchy. We called our approach Calder to draw a suggestive analogy between the nonstatic nature of compartment domain hierarchies and the mobile sculptures of Alexander Calder (Supplementary Fig. 1a).

### Chromatin subcompartments and compartment domains

First, we used Calder to infer chromatin compartmentalization in seven cell lines analyzed by Hi-C at high data resolution (Supplementary Data 1 and 2). Note that, in the following, we will refer to the total number of reads of an Hi-C experiment as its data resolution, which is related but distinct from the Hi-C map resolution, which typically refers to the bin size of the Hi-C contact map. At the top of the hierarchy, Calder identified A and B compartments that were largely coincident with those determined using previous approaches^2,4,18^ (Supplementary Fig. 1b). Descending the hierarchy, subcompartments could be explored at different levels of granularity (Fig. 1a) and were independent of data normalization (Supplementary Fig. 2a). Given that chromatin compartmentalization is expected to be associated with histone modifications^7^, we determined a biologically meaningful number of subcompartments by analyzing chromatin immunoprecipitation and sequencing (ChIP-seq) data for seven histone marks. We found that eight subcompartments were sufficient to capture significant changes of these histone modifications in all cell lines (Fig. 1b): 4 within the A compartment (A.1.1, A.1.2, A.2.1, A.2.2) and 4 within the B compartment (B.1.1, B.1.2, B.2.1, B.2.2). Histone modifications associated with active transcription were overall enriched in A.1.1, A.1.2, and A.2.1 subcompartments, whereas the repressive chromatin mark H3K27me3 was enriched in the intermediate subcompartments B.1.1 and B.1.2, and the heterochromatin mark H3K9me3 in B.2.1 and B.2.2 (Fig. 1c and Supplementary Fig. 2b). To infer chromatin activity states from histone modifications, we used the ChromHMM algorithm^22^ and confirmed the associations between active states (e.g., active promoters and enhancers) and A.1.1-A.1.2 and between heterochromatin and B.2.1-B.2.2 (Fig. 1d). Interestingly, this analysis further highlighted the associations between intermediate subcompartments and poised chromatin states, with poised promoters enriched in A.2.1-to-B.1.1 and H3K27me3-rich polycomb repressed chromatin in B.1.1-to-B.2.1 (Fig. 1d). These results show that the broad dichotomy into A and B compartments is insufficient to capture the diversity of chromatin states and their biological activity.

Next, we compared Calder 8 subcompartments with those inferred using the few available approaches. First, we used the adapted K-means (AKm) approach, originally used to detect three subcompartments^16^, to infer k = 8 subcompartments. However, AKm sometimes failed to call the desired number of compartments, a behavior already observed in a previous study^23^. Correlations between Akm compartment assignments and average histone mark intensities or mRNA expression were consistently lower than observed for Calder subcompartments (Supplementary Fig. 3a). Next, we compared Calder 8 subcompartments to the previously proposed partition into 5–6 subcompartments^2^: A1, A2, B1, B2, B3, and B4 (the latter however is specifically associated with a ~11MB region of Chr. 19 in GM12878). Although the original GHMM approach was applied only to the GM12878 Hi-C map, a Hi-C contact imputation approach called SNIPER^18^ was later proposed to enhance data resolution in five additional Hi-C maps^2^ (IMR90, HMEC, K562, HUVEC, and HeLa) and infer five subcompartments in these models. Subcompartments inferred by Calder and SNIPER in these six cell lines were highly concordant (Supplementary Fig. 3b) although those identified by Calder exhibited a stronger association with histone modifications and transcriptional activity than the ones identified by SNIPER (Supplementary Fig. 3a). To test the robustness and versatility of the two approaches, we inferred subcompartments in 38 cell lines representing diverse conditions and with different data resolution. Here, we found that SNIPER frequently called highly unbalanced subcompartments (Supplementary Fig. 4a): in 50% of the cases at least one subcompartment (most often A1) accounted for less than 1% of the genome, and in eight cases the method could not identify all five subcompartments (Supplementary Data 3). In these unbalanced cases, subcompartment assignments did not correlate with transcriptional activity, further suggesting that these did not reflect the true chromatin compartmentalization (Supplementary Data 4). Vice versa, Calder always called balanced subcompartments (Supplementary Fig. 4a) exhibiting high correlation with transcriptional activity (Supplementary Data 4). In particular, we found that SNIPER failed to call subcompartments in cell lines where interchromosomal contacts exhibited different frequency distributions from what was observed in the GM12878, which SNIPER uses as training dataset. This was most evident in cell lines that exhibited chromosomal translocations, which are frequent in cancer (e.g., see Supplementary Fig. 4b). Furthermore, by inferring subcompartments at high map resolution (10 kb bin), Calder revealed fine subcompartmentalization of the chromatin that could not be captured by SNIPER (100 kb bin). Representative examples showed that a shift to intermediate subcompartments, such as B.1.1 or A.2.2, was associated with increased H3K27me3 even in regions smaller than 100 kb, or that transitions from A.1.1 to A.1.2 and A.2.2 was associated with decreasing H3K27ac peak intensity (Fig. 1e). Overall, these results confirmed that histone mark heterogeneity is tightly linked to chromatin subcompartmentalization, and Calder can robustly reveal this association across multiple experiments and with greater detail than previous approaches.

**Fig. 2:**
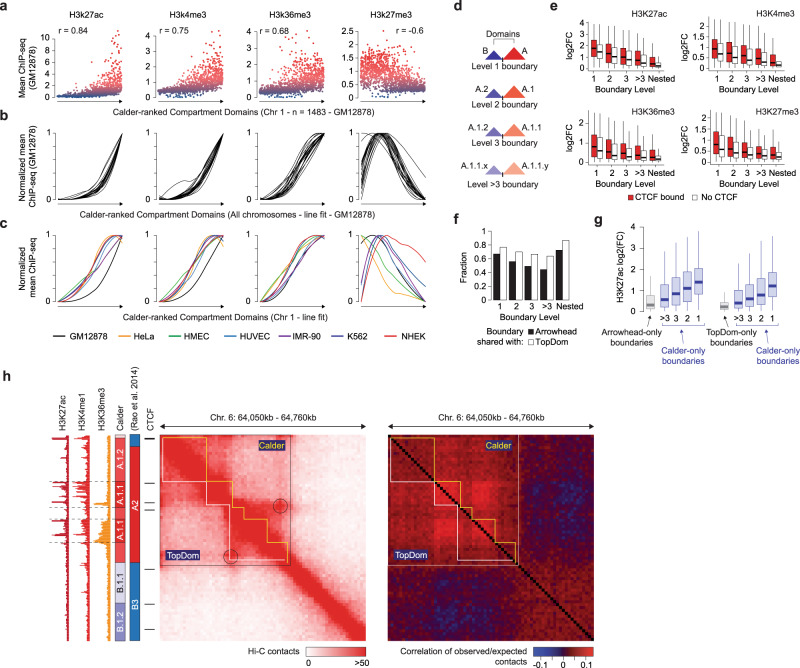
Association between compartment domains, topologically associating domains, and histone modifications. **a** ChIP-seq intensity of H3K27ac, H3K4me3, H3K36me3, and H3K27me3 for chromosome 1 compartment domains in GM12878. Each dot is a chromatin domain color coded by ChIP-seq intensity (blue-to-red). Domains were ordered by Calder-derived domain rank. *r* values are Spearman’s correlation coefficients. All correlation tests return *p*-values < 1E-100. **b, c**) LOESS fit lines of ChIP-seq intensity of H3K27ac, H3K4me3, H3K36me3, and H3K27me3 based on domain rank, for 22 chromosomes of GM12878 (**b**), and for chromosome 1 of GM12878, Hela, HMEC, HUVEC, IMR90, K562 and NHEK (**c**). Lines were normalized to the same maximum value. **d** Compartment domain boundaries are classified (Level 1, 2, 3, >3) based on the distance in the hierarchy of the domains separated by the boundary. **e** Fold-change of H3K27ac, H3K4me3, H3K36me3, and H3K27me3 ChIP-seq intensity at boundaries in GM12878, grouped by their level (from left to right) and presence/absence of CTCF (color coded). Each panel comprises n samples per boxplot with *n* = 1716, 2531, 2462, 2824, 2006, 2007, 2278, 1979, 850, and 1640 (from left to right). **f** Fraction of compartment domain boundaries inferred by Calder and inferred as TAD boundaries by arrowhead (black bars) and TopDom (white bars). **g** H3K27ac fold-changes at domain boundaries exclusively inferred by arrowhead (gray left), TopDom (gray right), and Calder (blue). Each panel comprises n samples per boxplot with *n* = 2296, 1411, 2339, 2054, 2383; 5860, 1003, 1597, 1351, 1551 (from left to right). **h** Visual comparison of compartment domains inferred by Calder (yellow track) and TADs inferred by TopDom (white track) on a Chr. 6 genomic region of the GM12878 cell line. The corner of the TAD identified by TopDom but not by Calder is circled. Domain tracks are overlaid to the Hi-C contact map (left) and the matrix of correlations of observed/expected contact ratios (right), which has been previously shown to reveal chromatin compartments. Subcompartment annotations and ChIP-seq tracks for H3K27ac (dark red), H3K4me1 (red), and H3K36me3 (orange) are shown on the left. The bounds of the boxplots in the plot are first quartile (*Q*_1_) and third quartile (*Q*_3_). The lower and upper whiskers are computed by extending the box bounds by 1.5*IQR, where *IQR* = *Q*_3_ − *Q*_1_.

At the bottom level of the hierarchy, compartment domains were clustered and ranked based on their 3D interdomain contacts and, indeed, the distance between two domains was anticorrelated with the mean number of interdomain interactions (Supplementary Fig. 5a). Compartment domain ranks were highly associated with histone mark intensities. For example, markers of active enhancers (H3K27ac), promoters (H3K4me3), and transcribed regions (H3K36me3) were all positively correlated with the ranks of chromosome 1 domains in GM12878 whereas, H3K27me3 showed significant anticorrelation (Fig. 2a). These trends were confirmed for all chromosomes (Fig. 2b) and all cell lines used for testing (Fig. 2c). Whereas several TAD callers have been proposed in the literature^24^, compartment domain boundaries have been simply inferred as genomic positions where a change of A/B compartment or subcompartment occurs^10^ or as positions where there is a switch of contact propensity towards either A or B compartments^25^. We obtained compartment domain boundaries using these approaches in the GM12878 cell line, based on available subcompartment annotations^2^. On the one hand, compartment domain boundaries inferred by Calder covered 92–93% of boundaries determined based on these annotations and approaches (Supplementary Fig. 5b). On the other hand, Calder inferred a much larger number of boundaries, consistent with its ability to infer a finer chromatin compartmentalization than other approaches. To explore the features of boundaries identified exclusively by Calder, we computed fold-changes of histone mark intensity between the contiguous domains that they separated. Histone mark fold-changes were significantly higher than expected at these boundaries (Supplementary Fig. 5c), supporting that they delimited domains within different subcompartments. To further explore the features of compartment domain boundaries inferred by Calder, we assigned each boundary to one of four classes based on how far apart in the dendrogram were the domains it separated (Fig. 2d). Histone mark fold-changes were greater when the two domains were assigned to distinct A and B compartments (Level 1) and decreased as the domains were assigned to more similar subcompartments, independently of CTCF binding status (Fig. 2e). Boundaries separating nested domains (Calder Step 3—Fig. 1a) exhibited the lowest histone mark fold-changes (Fig. 2e). Interestingly, the enrichment of CTCF and cohesin binding also decreased from level 1 to level >3 compartment domain boundaries, but it was greatest at nested boundaries (Supplementary Fig. 5d). These results suggest that nested domain boundaries are not compartment domain boundaries, but they are more likely to be associated with structural loops or TADs.

To further assess the overlap and differences between compartment domain boundaries inferred by Calder and TAD boundaries inferred by other callers, we compared our results in the GM12878 cell line with those obtained using TopDom^26^ and Arrowhead^2^, two top performing TAD callers as evaluated in a recent benchmarking study^24^. Between 50% and 70% of Calder compartment domain boundaries were within one bin distance (±10 kb) from TAD boundaries inferred by TopDom or arrowhead, but this percentage increased to 75–85% for nested domain boundaries (Fig. 2f). Genome-wide, boundaries inferred exclusively by one of these tools exhibited different features. Calder-specific boundaries were associated with greater changes of histone mark intensities than arrowhead- or TopDom-specific boundaries (Fig. 2g). Conversely, even though all boundaries identified by only one method were less enriched for CTCF and cohesin binding, this trend was particularly evident for Calder-specific boundaries (Supplementary Fig. 5e). Visual inspection of genomic regions where Calder and TopDom identified different boundaries further confirmed that TopDom boundaries delimited domains exhibiting local contact enrichment consistent with loop domains or TADs (Fig. 2h—left-side map). Conversely, Calder boundaries were aligned with the ‘plaid’ pattern characteristic of chromatin compartments, (Fig. 2h—right-side map). Lastly, we analyzed independent Hi-C cohorts generated before and after degradation of CTCF^9^ or deletion of the cohesin loading factor Nipbl^11^. Across both comparisons, we observed a consistent loss of nested domain boundaries, with 44% and 37% fewer nested boundaries upon CTCF and cohesin removal, respectively (Supplementary Fig. 5f). Interestingly, after Nipbl deletion, we found that loss of nested boundaries was accompanied by an increased number of compartment domain boundaries inferred by Calder (Supplementary Fig. 5f), consistent with the fine compartmentalization reported after cohesin depletion^11^. The different effect on compartment domain boundaries induced by CTCF and cohesin depletion was consistent with evidence that only cohesin loss impairs loop extrusion and lead to fine compartmentalization^7^. These results demonstrate that Calder specifically infers compartment domains, which are associated with histone post-translational modifications and, although often overlapping, are not coincident with TADs.

### Compartment domain inference and repositioning across >100 cell lines

**Fig. 3:**
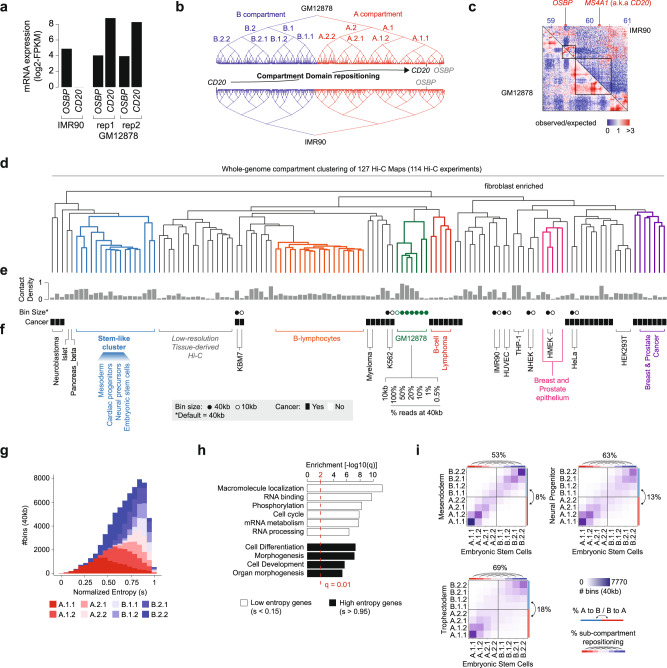
Subcompartments and compartment domain repositioning analysis across 114 cell lines. **a** mRNA expression levels (RNA-seq, log-transformed FPKM) of *OSBP* and *CD20* (gene name: *MS4A1*) in the IMR90 cell line and two replicates of the GM12878. **b** Chromatin domain hierarchy built by Calder for chromosome 11 in IMR90 (bottom) and GM12878 (top). TADs containing OSBP and CD20 and the 8 subcompartments are indicated in the hierarchy. The CD20 domain is repositioned from B.2.2 to A.1.1 compartment (arrow). **c** Observed/expected (O/E) contact map of GM12878 (lower triangle) and IMR90 (upper triangle) at Chr.11:58,700–61,000 kb. The *OSBP* and *MS4A1* gene loci are indicated in red. **d**–**f** Clustering of 127 Hi-C maps based on the subcompartment annotation of each bin (8-compartment model). Hi-C maps are annotated with their contact density (histogram), bin size for maps analyzed at different bin sizes, and based on whether they were derived from a healthy or cancerous tissue (black boxes) (**e**). Representative clusters and cell lines are highlighted (**f**). **g** Compartment entropy distribution of genomic bins colored by their compartment annotation. **h** Gene ontology enrichment [-log10(q-value)] for genes in low (S < 0.15) and high (S > 0.95) entropy bins. **i** Comparison of the number of bins assigned to each compartment (color coded) between H1 embryonic stem cells (*X*-axis) and H1 lineage differentiation in mesendoderm (left), neural progenitors (center), and trophectoderm (right).

Consistent with the chromatin epigenetic status, compartment domains ranks correlated with transcriptional activity (Supplementary Fig. 6a). As transcriptional activity is highly variable among different cell types, we wondered whether this variability is reflected in spatial repositioning of compartment domains. We first tested this hypothesis by comparing the position of the domain containing the B-cell marker CD20 (gene name *MSA41*) in lymphoblastoid cells (GM12878), where the gene is highly expressed, and in lung fibroblasts (IMR90), where the gene is silenced (Fig. 3a). Calder inferred a drastic repositioning of the *CD20*-containing domain between IMR90 (rank: 0.0035, B.2.2 subcompartment) and GM12878 (rank: 0.92, A.1.1 subcompartment). Conversely, we did not observe any repositioning for the domain containing the *OSBP* gene (rank_IMR90_: 0.92, rank_GM12878_: 0.98, A.1.1 subcompartment) (Fig. 3b), which is proximal to *CD20* in the genome sequence but similarly expressed in the two cell lines (Fig. 3a). *CD20* repositioning was associated with a different enrichment of Hi-C contacts at its locus in GM12878 and IMR90, whereas contact frequencies were similar at the *OSBP* locus (Fig. 3c). Notably, previous approaches to infer TAD hierarchies always preserved 1D-proximity, i.e., genome sequence contiguity, and thus cannot be used to capture repositioning events (Supplementary Fig. 6b).

To explore chromatin subcompartments and compartment domain repositioning across a broader and more diverse set of conditions, we collected Hi-C data for 114 cell lines and tissue samples (Supplementary Data 1 and 2, Supplementary Fig. 7a). To assess the robustness of our approach to Hi-C data and map resolution, we analyzed 8 cell lines using different bin sizes (10 kb and 40 kb) and five downsampled versions of the GM12878 interaction map, retaining only 50, 20, 10, 1, and 0.5% of its total number of contacts. Overall, we clustered 127 Hi-C datasets based on Calder subcompartment (*n* = 8) assignments for each bin (Supplementary Data 5). Cell lines clustered largely based on their lineage instead of resolution (Fig. 3d–f and Supplementary Fig. 7b), as shown by downsampled or differentially binned contact maps of the same cell line that always clustered together (Fig. 3c). For example, B-lymphoblastoid GM12878 contact maps (Fig. 3f—green cluster) and cancer cell line models of B-cell malignancies (Fig. 3f—red cluster) formed two separate but contiguous clusters, and together with other B-lymphocytes were characterized by the repositioning of the CD20-containing domain to the active compartment (Supplementary Fig. 7c). Other lineage-associated clusters included a stem-like cluster comprising cardiac and neural progenitor and embryonic stem cells (Fig. 3f—blue cluster), as well as two separate clusters comprising normal epithelial and cancer cells (e.g., breast and prostate normal and cancer cell lines—Fig. 3f—pink and purple clusters, respectively). A lineage-independent cluster was found for a set of low data resolution Hi-C experiments generated from tissue specimen (Fig. 3f). Even though other Hi-C maps at similar resolution clustered based on their lineage, we cannot exclude the possibility that our results were here affected by low read counts and/or other technical issues. Previous approaches used the first principal component of the contact enrichment correlation matrix (PC1) either to rank genomics bins within a chromosome (based on the values of PC1) or to determine A and B compartments (based on the sign of PC1). Clustering of the Hi-C datasets based on PC1 values was largely dependent of data and map resolution, failing to recover lineage-associated clusters and clustering apart even Hi-C profiles from the same cell line (Supplementary Fig. 8a,b). Clustering using the sign of PC1 (i.e., A/B compartment calls) was less sensitive to resolution, but still could not recover lineage-associated clusters to the same extent of the 8-subcompartment classification inferred by Calder (Supplementary Fig. 8a, c). Finally, to further corroborate the relevance of intermediate compartments, we compared Hi-C maps from stem-like cell lines and fully differentiated tissues, using exclusively genomic bins that were assigned to the same compartment (A or B) in all cell lines, but that differed at the level of subcompartments. Clustering based on these bins perfectly separated stem-like models from differentiated tissues (Supplementary Fig. 8d).

Using Shannon’s information entropy (S), we examined which regions were found in the same subcompartment across multiple cell lines (low entropy) and which instead were frequently repositioned (high entropy). The overall *c*ompartment entropy was significantly lower than expected by chance (Supplementary Fig. 9a), and it was higher in cancer than in normal cell lines (Supplementary Fig. 9b), suggesting that tumor molecular heterogeneity is also reflected in structural heterogeneity. Low-entropy bins were almost exclusively assigned to the most extreme compartments, i.e., B.2.2 and A.1.1. In contrast, intermediate subcompartments only appeared in bins with high-entropy values (Fig. 3g), indicating that genomic regions in an intermediate subcompartment in one cell line are more likely to be repositioned in other cell lines. Among the 114 cell lines that we analyzed, on average 49–54% of regions in A.1.1 or B.2.2 in a given cell line changed subcompartment in another, as opposed to 80–85% of regions in the intermediate subcompartments (A.2.1 to B.1.2) (Supplementary Fig. 9c). Protein-coding genes located in genomic regions of low entropy (S < 0.15, *n* = 532) were significantly enriched for fundamental cellular processes, such as protein transport, localization, and phosphorylation, RNA processing and metabolism, and cell cycle (Fig. 3h—top). Vice versa, high-entropy genes (S > 0.95, *n* = 315) were significantly enriched for cell differentiation, development, and morphogenesis (Fig. 3h—bottom and Supplementary Data 6). To explore how lineage differentiation is reflected in compartment repositioning, we examined Hi-C maps of H1 human embryonic stem cells and 3 H1-derived lineages. Compartment hierarchies exhibited extensive compartment changes. However, in each comparison, only a minor fraction corresponded to A to B (or B to A) switches, whereas in all comparisons more than 50% of the genome exhibited subcompartment repositioning (Fig. 3i). Overall, these results suggest that subcompartments repositioning is frequent and potentially associated with lineage commitment and cell differentiation.

**Fig. 4:**
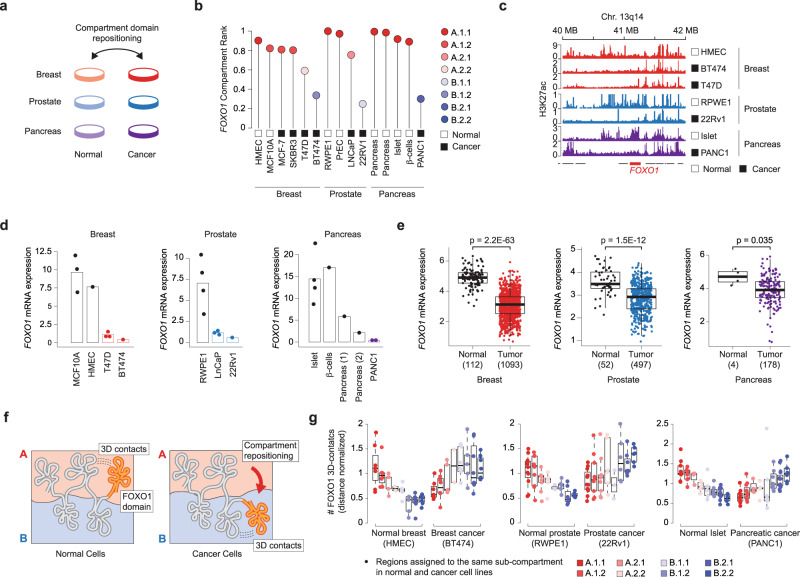
Subcompartment repositioning between normal and cancer cell lines. **a** Schematic comparison of the comparison between normal and cancer cell lines from breast (red), prostate (blue), and pancreas (purple) tissues. **b** Compartment (color coded) and domain rank (*Y*-axis) of the *FOXO1*-containing domain in normal and cancer cells. **c** H3K27ac intensity at the *FOXO1* locus for normal and cancer cell lines derived from breast (red), prostate (blue), and pancreatic (purple) tissues. **d**, **e** *FOXO1* mRNA expression level (log2-transformed FPKM normalized RNA-seq reads) in either the cell lines with matching Hi-C data (**d**), or in normal and cancer samples profiled by The Cancer Genome Atlas (TCGA) (**e**). Each data point represents a replicate in (**d**, *n* = 3, 1, 3, 1–4, 3, 1–4, 1, 1, 1, 2) and a sample in (**e**, numbers of samples in brackets). **f** Schematic representation of interdomain interactions (dashed lines) between the *FOXO1*-containing compartment domain (yellow) and regions that do not change compartment in normal and cancer cells (gray). The *FOXO1*-domain is repositioned in cancer cells. **g** Distance normalized contact values between *FOXO1* and regions that do not change compartment in the compared breast (left, *n* = 7, 7, 4, 2, 5, 5, 7, 8), prostate (center, *n* = 11, 10, 9, 6, 3, 6, 7, 6), and pancreatic (right, *n* = 9, 12, 4, 5, 6, 8, 16, 6) cell lines. The bounds of the box in the plot are first quartile (*Q*_1_) and third quartile (*Q*_3_). The lower and upper whiskers are computed by extending the box bounds by 1.5*IQR, where *IQR* = *Q*_3_ − *Q*_1_.

Lastly, we investigated subcompartment repositioning in malignant transformation and analyzed three relatively homogeneous groups of cell lines including normal and cancer cells derived from breast, prostate, and pancreatic tissues (Fig. 4a). Subcompartment repositioning between cancer and normal cells was associated with changes in gene expression (Supplementary Fig. 9d) and these were on average higher with greater repositioning. By focusing on common subcompartment repositioning events between normal and cancer cell lines (Supplementary Data 7), we found a shift from active to inactive compartment of the Forkhead box O transcription factor *FOXO1*, which has been frequently associated with tumor suppressive functions^27,28^. The *FOXO1* locus was in A.1.1 or A.1.2 in all normal cells and it shifted towards intermediate and inactive compartments in cancer cell lines (Fig. 4b). Although it was not genetically altered in these cells^29^, *FOXO1* repositioning was associated with loss of H3K27ac upstream of the gene (Fig. 4c) and downregulation of *FOXO1* mRNA expression (Fig. 4d). Interestingly, by analyzing large human tumor cohorts profiled by The Cancer Genome Atlas^30^ (TCGA), we found that *FOXO1* was downregulated in the vast majority of human breast, prostate, and pancreatic tumors (Fig. 4e) despite being only rarely target of genetic alterations in these tumor types: ~8% in prostate, <2% in breast, and never in pancreatic cancer (source cBioPortal^31^). To verify that subcompartment repositioning was effectively associated with modified 3D-proximity to other domains, we analyzed intrachromosomal contact frequencies between the *FOXO1*-containing compartment domain and other compartment domains that remained in the same subcompartment in normal and cancer cell lines (Fig. 4f). In normal cell lines, *FOXO1* interacted more frequently with regions in active than with regions in inactive subcompartments, but this trend was reversed in cancer cells (Fig. 4g), supporting a physical repositioning of the *FOXO1* locus in the chromatin 3D structure. Subcompartment repositioning events hence allow to map transcriptional changes to modified chromatin interactions, pinpointing associations between gene expression and spatial organization.

## Discussion

Computational analyses of DNA interaction maps generated by Hi-C experiments have allowed to define the structural building blocks of chromatin architectures, such as loops, TADs, (sub-) compartments and compartment domains^13^. In contrast to TADs, the inference and comparison of chromatin subcompartments and compartment domains has so far been limited to a handful of cell lines, mostly due to the limited number of approaches for this task, and their demanding data resolution requirements. Here we showed that Calder outperformed previous approaches on high data resolution experiments and, especially, it enabled this type of analyses on a wide variety of cell lines at different resolutions.

We showed that subcompartment differences are extensive between different cell lines, even when domain boundaries are preserved, and these differences were often associated with epigenetic and transcriptional heterogeneity among tissue types, differentiation stages, and malignant states. These results caution against assuming that subcompartments partitions derived from one cell line can be applied to other samples. Intriguingly, the most different subcompartments (i.e., A.1.1 and B.2.2) were the most stable across cell lines, whereas regions in intermediate subcompartments were rarely the same across samples. These findings indicate a different degree of chromatin plasticity along the genome. Future studies should investigate the biological relevance of this plasticity and whether it reflects the chromatin conformation of individual cells or the average conformation of a heterogeneous cell population.

Moreover, we found that subcompartment repositioning was frequently associated with changes of gene expression and enrichment for different histone marks. Although the chain of causality of these events needs further investigation, recent evidence indicated that altering histone modifications is sufficient to increase and decrease the frequency of chromatin interactions^32,33^ or lead to changes in chromatin compartmentalization^34^. In this context, robust inference of chromatin subcompartments and compartment domains will be crucial to appreciate the effect of altered epigenetic states on chromatin compartmentalization.

Overall, the identification of chromatin subcompartments across a variety of cell types and conditions opens the possibility to their systematic comparison and quantification of spatial repositioning to aid the understanding of the link between structure and phenotype.

## Methods

### Generating Hi-C contact matrices

Hi-C intrachromosomal contact maps (or matrices) were either generated from raw FASTQ files using Juicer tools^35^ with default parameters or obtained from processed public data. Data sources are provided in Supplementary Data 1. The Knight-Ruiz (KR) method was used for contact matrix normalization when the source data was not normalized. In cases when KR normalization failed to converge, the VR (vanilla coverage) normalization was applied. All Hi-C contact maps were binned at 40 kb resolution. Contact maps of GM12878, HUVEC, IMR90, K562, NHEK, HMEC, KBM7, and HeLa were additionally analyzed using 10 kb bins. In addition, for subcompartment comparisons, SNIPER was run with a bin size equal to 100 kb and Adapted k-means with a bin size equal to 1 Mb. Contact maps from tamoxifen-control and cohesin-removed datasets were binned at 20 kb. The 100 kb, 1 Mb, or 20 kb bin sizes were chosen to be consistent with the setting used in the study where each method was original proposed. Lastly, we generated six downsampled contact maps for GM12878 at 40 kb resolution by randomly retaining 50, 20, 10%, 1, or 0.5% of the total contact reads. To reduce technical noise within regions of low mappability, matched rows and columns with more than 99% values equal to 0 were removed. For each processed contact map, we defined its contact density as the fraction of non-zero entries. Only autosomal chromosomes were analyzed.

### Analyzing ChIP-seq data of histone modifications

Processed ChIP-seq value (fold change over control, pooled replicates) were retrieved from ENCODE [https://www.encodeproject.org/] or GEO [https://www.ncbi.nlm.nih.gov/geo/]. We also processed H3K27ac for BT474 from publicly available raw sequencing data. FASTQ files were aligned to hg19 reference genome by bowtie2^36^ version 2.3.4.3 with local alignment option. Peaks of H3K27ac enrichment compared to the corresponding input control were identified using MACS2^37^ version 2.1.1. Extreme values, defined as those greater than the upper 0.999 quantile of each chromosome, were replaced with the 0.999 quantile. Data sources are provided in Supplementary Data 1.

### Quantification of gene expression

Gene expression values at mRNA level for cell lines were quantified by FPKM (fragments per kilobase per million mapped fragments) and were retrieved from ENCODE or processed from publicly available raw FASTQ files using RSEM^38^ v.1.3.1. For running RSEM, we set hg19 as the reference genome and used STAR^39^ 2.7.0d for alignment. Other parameters were set as default. We also retrieved gene expression values for normal and cancer human samples from The Cancer Genome Atlas (TCGA) collected in the FireHose data repository [https://gdac.broadinstitute.org/]. We used TPM (transcripts per million) as a measure of gene expression level for these samples. Data sources are provided in Supplementary Data 1.

### The Calder Algorithm

#### Step 1: Compartment Domain Calling

We defined compartment domains as consecutive bins having high intradomain similarity while low interdomain similarity, where the similarity is measured based on whole-chromosome interaction pattern. Identification of compartment domains proceeds through the following steps (Supplementary Fig. 10a).

1. Providing an intrachromosomal contact matrix A, we generated an aggregated matrix A* if the bin size is smaller than 40 kb, by compressing every 10 columns into a single column, thus the i_th_ row of A* represents the total contacts between bin_i_ and every 10 bins. If the bin size is ≥40 kb we omit this step thus A* = A. The purpose of this step is to sum up contact values to reduce data sparsity for small bin size, thus, to allow applying our method to bin size up to 10 kb.
2. A correlation matrix ρA was computed storing all pairwise correlations of the rows of A*. To enhance the correlation signals, we computed pairwise column correlations again from ρA to generate a second correlation matrix ρ_2_A.
3. ρ_2_A was transformed into a similarity matrix defined as sA = arctanh(ρ_2_A). The function arctanh was chosen such that correlations with absolute value greater than ~0.3 have a greater amplification ratio. This is also commonly referred to as Fisher z-transformation, which the converted correlation score is approximately normally distributed.
4. Finally, we called compartment domain boundaries separating regions having high intraregion values and low inter-region values. To this purpose, we used the same strategy proposed in the TopDom algorithm applied to the sA matrix (instead of the Hi-C contact matrix).

#### Step 2: Deriving the hierarchy of chromatin domains

Given *k* chromatin domains identified at Step 1, we derived a clustering dendrogram based on the similarity of whole-chromosome interdomain contacts. To build a hierarchy of chromatin domains, we proceed through the following steps (Supplementary Fig. 10b).

1. First, we computed 4 raw trend matrices T^*m*^, with m = 1,…,4, each of dimension (k-m) × k, to summarize the plaid pattern commonly observed in the Hi-C contact matrix and that reflects compartmentalization. Values of T^*m*^ are defined as: T^*m*^(*i*,*j*) = 1 if the mean contact value between bins in domain *i* and in domain *j* + *m* is greater than the mean contact value between bins in domain *i* and bins in domain *j* (thus indicating enrichment of contacts); otherwise T^*m*^(*i*,*j*) = 0 (depletion of contacts). These four trend matrices were concatenated by rows to form a combined trend matrix T. A correlation matrix ρT was computed storing all the pairwise correlations of the columns of T and then converted to a score matrix sT using arctanh transform. Although a single raw trend matrix (m = 1) can also be used to compute the correlation in this step, we found the concatenation of multiple matrices can increase the correlation signals, however the marginal gain is negligible when the number if greater than 4.
2. Next, we used the first 10 principal components (PCs) of sT to perform a divisive hierarchical clustering. Starting from the complete set of compartment domains, we iteratively split a set into two sub-sets using k-means (k = 2), until each compartment domain is on a separate set. PC values can be rescaled by a factor w, to give different weight to different PCs. Given the first PC (PC_1_) was the most associated with chromatin compartmentalization (similarly to the PC_1_ of normalized Hi-C maps^4,40^), in the analyses presented in this manuscript, we set w = 1 for PC_1_ and w = 0.25 for all other PCs. We chose the first 10 PCs in this step as we observed the marginal variance explained by each additional PC is negligible.
3. Fianlly, since there are multiple possible ways to order the dendrogram branches without disrupting its structure, we sought a consistent ordering among chromosomes reflecting chromatin compartmentalization.
  - Given the sign of PCs is arbitrary, we fixed the sign of PC_1_ such that it correlates with gene density (gene density is expected to be higher in A than in B compartment).
  - Then we derived the non-linear major axis of the point cloud (each point represent one domain) formed by PC_1_ and PC_2_ using loess regression, i.e. loess(PC_2_ ~ PC_1_), and projected each data point, i.e., domain, [PC_1_(x),PC_2_(x)] to this major axis.
  - To each domain we assigned a score *z* based on its ranking *j* of this major axis: ${z}_{j}=0$ if $j=1$; and ${z}_{j}={\sum }_{i=2}^{j}\sqrt{{({x}_{i}-{x}_{i-1})}^{2}+{({y}_{i}-{y}_{i-1})}^{2}}$ if *j* > 1, which is the Euclidean distance of between each pair of adjacent domains along this axis.
  - Finally, we ordered the branches of the dendrogram such that the branch on the right side should have a greater average *z* value than the branch on the left side, for any sibling branch pair.

The left and right branch at the top level of the ordered dendrogram were labelled as B and A respectively. For a sub-branch X, its left child branch was labelled as X.2 and right child branch as X.1. The chromatin domain ranks were derived from the order of the domains in the final dendrogram and normalized by the total number of chromatin domains in that dendrogram.

#### Step 3: Calling for nested subdomains

A stochastic approach was developed to call nested subdomains within each compartment domain exclusively based on short-range contacts. The basic idea is to determine whether a hierarchical structure of smaller domains can be further inferred from local contact patterns within a given compartment domain. In this step of the Calder (and unlike what is done in the previous step) hierarchies of nested domains contained in a domain are anchored to the genome sequence, i.e., adjacent nested domain in the hierarchy are also contiguous along the genome sequence. This step can be split into two major tasks: (1) finding the best-fitting dendrogram D; (2) trimming D to get meaningful nested domains.

1. Finding the best-fitting dendrogram: We assume the contact probability between bins from two sibling domains in the dendrogram D follows a mixture log-normal distribution with parameter ${{\boldsymbol{\theta }}}_{k}=\{{\alpha }_{k},{\mu }_{k},{\sigma }_{k}\}$
  $${p}_{{{\mathrm{mix}}}}\left(x,|,{\alpha }_{k},{\mu }_{k},{\sigma }_{k}\right)={\alpha }_{k}{p}_{0}\left(x\right)+\left(1-{\alpha }_{k}\right){p}_{1}(x{\rm{|}}{\mu }_{k},{\sigma }_{k})$$
  where ${p}_{0}(x)$ is the density function of a degenerate distribution which has P(*X* = 0) = 1; ${p}_{1}(x)$ is the density of a log-normal distribution which has ${\rm{ln}}\left(X\right) \sim N(\mu ,{\sigma }^{2})$.
  Given the contact matrix $A$, the likelihood function of the dendrogram and the associated parameters is given by
  $$L( D, \{ {{\boldsymbol{\theta }}}_{k} \} {|} A ) = \mathop{\prod}\limits_{k \{ {\mathrm{all}}\, {\mathrm{sibling}}\, {\mathrm{pairs}}\}} {p}_{{\mathrm{mix}}} (A {|} {{\boldsymbol{\theta }}}_{k})$$
  We computed the best-fitting dendrogram and corresponding parameters using maximum likelihood estimation
  $$\{D; \{{{\boldsymbol{\theta}}}_{k}\} \} ={{\rm{argmax}}}_{D; \{ {{\boldsymbol{\theta }}}_{k} \}} L(D, \{ {{\boldsymbol{\theta }}}_{k}\} {|}A),$$
  which can be computed via dynamic programming and has a computation complexity of $O({n}^{3})$, where $n$ is the number of bins in the chromatin domain under investigation (Supplementary Fig. 10c—left).
2. Trimming the dendrogram to get meaningful nested domains: Given the best-fitting dendrogram D, we extracted meaningful nested domains by splitting iteratively a domain into subdomains as described below.
  - First, we compute the observed vs. expected contact matrix A_oe_ to eliminate distance-bias of the contact matrix A_oe_ is defined as the ration between the contact maps A and E, where E is the expected contact matrix with:
    $${E}_{i,j}={{\mathrm{mean}}}({A}_{j,k}{\mathrm{for}}\, {\mathrm{all}}\left|j-k\right|{\mathrm{equal}}\, {\mathrm{to}}|i-j{\rm{|}}).$$
  - A_oe_ within a given domain is split into four parts: **A:** the triangle delimited by the off-diagonal corner of the domain and the two off-diagonal corners of the candidate nested domains; **B:** the triangle comprising the contacts between the two nested domains that are not included in (a); **C–D:** contacts within the two nested domains (Supplementary Fig. 10c—right).
  - Finally, a given split was accepted if (1) contacts within **A** were significantly higher than those in **B** and (2) contacts in **C** and **D** were significantly higher than contacts in **B**. Contact differences are tested using a one-tailed Wilcoxon test and considered as significant if *p*-value < 0.05 and $\Delta {{\mathrm{mean}}}$ between the compared regions is greater than 0.1 (0.1 is equivalent to 10% of relative difference given that the expected mean in any region of the distance-corrected matrix A_oe_ is 1).

The above procedures result in a trimmed dendrogram representing nested subdomains in a compartment domain.

### Number of compartments required to capture chromatin epigenetic pattern variation

To determine the number of compartments that is sufficient to explain the chromatin epigenetic pattern variation, we proceed as follow:

- For each histone mark, we first log-transformed (ln(x + 1)) bin-level ChIP-seq intensity values and took the mean of these for each chromatin domain.
- Next, given two sibling compartments in the domain hierarchy with mean intensity values X = {x_1_,…,x_n_} and Y = {y_1_,…,y_m_} and overall mean μ(X) > μ(Y), we defined the effect size Δμ as the maximum possible value such that μ(X)-Δμ is significantly greater than μ(Y) (one-tailed t-test, significance threshold α = 0.001).
- Mean intensity values of two adjacent compartments were considered as significantly different if the median Δμ of all 22 chromosomes was above a threshold γ. To penalize large number of compartments that did not lead to high gains in terms of explained variance (similar to what is done in a penalized regression), we used progressive thresholds: $\gamma =0.05$ for 2 and 4 compartments, $\gamma =0.1$ for 8 compartments, and $\gamma =0.2$ for >8 compartments.

We performed this test for each histone mark starting from the top (A/B compartment) and iteratively on lower levels of the hierarchy until no significant differences were reached. The number of compartments corresponding right before the stopping step was retained as the minimal number of compartment sufficient to explain epigenetic pattern variation.

### Comparison with (sub-)compartments identified by SNIPER and Adapted K-means

We applied SNIPER on 38 Hi-C datasets at the resolution of 100 kb. SNIPER is a machine learning-based method which first learned the relation between the interchromosomal contact pattern and compartment annotation of (Rao et al. 2014)^2^, then predict five compartments (labeled as A1, A2, B1, B2, B3) of a new dataset using the new interchromosomal contact pattern. The author provided five trained models for different downsample ratios (2%, 3%, 4%, 5%, 10%). We chose the downsample ratio that best matches the observed ratio between a given dataset and the training dataset of GM12878 (ratio is defined as total interchromosomal contacts of the given dataset divided by total interchromosomal contacts of the GM12878 dataset). We also applied Adapted k-means on 7 Hi-C datasets (GM12878, Hela, HMEC, HUVEC, IMR90, K562, and NHEK), using 1 Mb bin size as specified in the original paper. We set *k* = *8* with the aim to identify the same number of subcompartments as Calder. We ordered the inferred subcompartments based on the average H3Kme3 intensity of each subcompartment (By default, Adapted-kmeans does not give a label or rank to each compartment it infers).

#### Comparison with domain boundaries defined from other approaches

Compartment domain boundaries have been simply inferred as genomic positions where a change of A/B compartment or subcompartment occurs^10^, or as positions where there is a switch of contact propensity with A/B compartments^25^. For the first approach, we retrieved compartment domain boundaries based on the publicly available subcompartment annotation from^2^. For the second approach, we computed for each 10 kb bin the average distance-corrected contact intensity with A or B compartment. An A-B index was defined as the difference between the A/B intensity. Compartment domain boundaries were retrieved at positions where a switch of the sign (±) of A-B index occurs. Switches that occupy only 1 bin were considered as noise and omitted. To compare boundaries identified by these approaches or Calder, we considered two boundaries as overlapping if they are less than 100 kb apart (100 kb was the resolution of subcompartment annotation from^2^).

We also applied Arrowhead^2^ and TopDom^26^ to the Hi-C map of GM12878 at 10 kb bin size to call TADs, using default parameters. Two boundaries were considered as overlapping if they are less than 40 kb apart.

#### Inferring genome sequence-dependent hierarchies using TADpole

We applied TADpole to the Hi-C data of GM12878 binned at 40 kb to derive genome sequence-dependent hierarchies, for the purpose to compare with Calder’s genome sequence-independent hierarchies as shown in Supplementary Fig. 6b. Parameters for TADpole was set as *max_pcs* *=* *50*, *bad_frac* *=* *0* and *centromere_search* *=* *FALSE*.

#### Computing the chromatin entropy

Given the 8-compartment model, we defined the Shannon’s information entropy of a bin *b* as:

$${S}_{b}=-\frac{1}{{\rm{ln}}(8)}{\sum }_{j=1}^{8}{P}_{j}{\rm{ln}}({P}_{j})$$

where P_j_ is the frequency with which *b* is assigned to compartment *j* across the 114 datasets. The normalizing factor ln(8) ensures that S is in the [0,1] range. We defined the distribution of entropy values for all bins of the genome as chromatin entropy. To estimate the expected chromatin entropy, we performed 1000 permutations of compartment labels. In each permutation, we randomly shuffled the compartment labels of each bin, within each dataset independently. Chromatin entropies computed from each permutation were aggregated to derive the overall random distribution.

### Enrichment of histone mark intensities and ChromHMM states at compartments

For each histone mark, mean intensity values for each chromatin domain were computed. Next, we determined the enrichment of these values within a given compartment (8-compartment model) as the log_2_-transformed ratio between the median value in the compartment and the overall median value.

To determine the enrichment of ChromHMM states in each compartment, we used the 15-state definition reported in: [http://genome.ucsc.edu/cgi-bin/hgTrackUi?db=hg19&g=wgEncodeBroadHmm]

To each genomic region associated to a given state by ChromHMM, we assigned the domain rank value and compartment label of the chromatin domain overlapping with that region. If a region was covered by multiple chromatin domains, we assigned to that region the mean domain rank and the “least active” compartment label, i.e. the label closest to B.2.2. As a result, each of the 15 ChromHMM states were associated with a vector of domain ranks and a vector of compartment labels. To determine the enrichment of a given compartment label in a ChromHMM state, we computed the number of occurrences of each compartment label for regions annotated with a given ChromHMM state S, and divided these numbers by the expected values obtained by multiplying the number of regions in S by the vector of compartment label frequencies across the entire genome. Genomic regions that did not overlap with any chromatin domain were discarded in this analysis.

### Geneset enrichment analysis

Coding genes located in genomic regions of low entropy (S < 0.15, *n* = 532) and of high entropy (S > 0.95, *n* = 315) were tested for enrichment of Gene Ontology terms in the Molecular Function and Biological Process categories using the mSigDB web service [https://www.gsea-msigdb.org/gsea/msigdb/index.jsp] and retaining the top 100 solutions with adjusted *p*-value < 0.01. In the geneset enrichment analysis of genes with high entropy, we noticed strong enrichment for GO categories associated with cell adhesion (Supplementary Data 6). However, this enrichment was due to the presence of a cluster of protocadherin encoding genes within a single chromatin domain. For this reason, we decided to flag and disregard these results as they do not reflect functional similarities among genes in different high-entropy domains.

### Reporting summary

Further information on research design is available in the Nature Research Reporting Summary linked to this article.

## Supplementary information

Supplementary Information

Reporting Summary

Description of Additional Supplementary Files

Supplementary Dataset 1

Supplementary Dataset 2

Supplementary Dataset 3

Supplementary Dataset 4

Supplementary Dataset 5

Supplementary Dataset 6

Supplementary Dataset 7

## Data Availability

The data that support this study are available from the corresponding author upon reasonable request. All Hi-C, ChIP-seq, and RNA-seq datasets used in this study are publicly available and described in detail in Supplementary Data 1: Hi-C datasets were retrieved from the Gene Expression Omnibus (GEO) repository GSM1631184, GSE66733, GSE71831, GSE63525, GSE95014, GSE44267, GSE63525, GSE118514, GSM2809542, GSM3111886, GSE109229, GSE118588, GSE99051, GSM3112385, GSM3112403, GSE105194, GSE105318, GSE105381, GSE118514, GSE71072, GSM2334832, GSM1902603, GSM3262956, GSM3262958, GSM3262960, GSM3262962, GSM3262964, GSM3262966, GSM3263085, GSM3263087, GSM3263089, GSM3734950, GSM3734952, GSM3734958, GSM3734960, and the ENCODE repository ENCSR079VIJ, ENCSR105KFX, ENCSR312KHQ, ENCSR346DCU, ENCSR401TBQ, ENCSR444WCZ, ENCSR489OCU, ENCSR549MGQ, ENCSR834DXR, ENCSR862OGI, ENCSR440CTR, ENCSR504OTV; ChIP-seq datasets were retrieved from ENCODE ENCFF002DDH, ENCFF003CLZ, ENCFF010PHG, ENCFF038HNR, ENCFF039DZS, ENCFF039XYU, ENCFF041YQC, ENCFF046ABB, ENCFF062LIE, ENCFF104GFP, ENCFF107PYQ, ENCFF110GQH, ENCFF114EGM, ENCFF116RLU, ENCFF118MMT, ENCFF129XGI, ENCFF143CUR, ENCFF167NBF, ENCFF167TFD, ENCFF175PNA, ENCFF180LKW, ENCFF191EXE, ENCFF195CYT, ENCFF198ONW, ENCFF203KHF, ENCFF226MQR, ENCFF236IFZ, ENCFF240SOO, ENCFF240TPI, ENCFF242SAO, ENCFF247PJA, ENCFF254UZP, ENCFF255NQJ, ENCFF269UZG, ENCFF272ZUB, ENCFF282RLY, ENCFF283KXE, ENCFF312ELW, ENCFF322JKF, ENCFF335YSY, ENCFF388WMD, ENCFF391MRG, ENCFF396JIR, ENCFF420DLT, ENCFF421DIE, ENCFF432DSJ, ENCFF437CJP, ENCFF445UCR, ENCFF453XKM, ENCFF465KNK, ENCFF488GZA, ENCFF526QTS, ENCFF531OEA, ENCFF551YLH, ENCFF557RSD, ENCFF573VUK, ENCFF585TQE, ENCFF602QRW, ENCFF615INK, ENCFF662QFK, ENCFF674XOM, ENCFF678IWR, ENCFF682WPF, ENCFF693WQF, ENCFF699TXY, ENCFF707JNT, ENCFF709GNN, ENCFF738TKN, ENCFF758SEW, ENCFF761QZP, ENCFF768OPK, ENCFF790BNV, ENCFF790YPL, ENCFF792ZIY, ENCFF828CQV, ENCFF831WYD, ENCFF834HNV, ENCFF834YLI, ENCFF846MPX, ENCFF879AFU, ENCFF885XEM, ENCFF903WOV, ENCFF934OWP, ENCFF935IDJ, ENCFF958BAN, ENCFF977DET, ENCFF981TTA, ENCFF981WTU; RNA-seq datasets were retrieved from ENCODE ENCFF170MOO, ENCFF374KZN, ENCFF554RBN, ENCFF582THK, ENCFF612EVW and the Sequence Read Archive (SRA) SRR1634434, SRR2125669, SRR2125670, SRR2125671, SRR2125672, SRR2149928, SRR2149929, SRR2149930, SRR2149931, SRR2149932, SRR2149933, SRR2532390, SRR2567462, SRR2567463, SRR2567464, SRR3677548, SRR3677549, SRR3677550, SRR3677551, SRR3677552, SRR3677553, SRR4341895, SRR4341896, SRR4341897, SRR4341898, SRR4341899, SRR4341900, SRR5120472, SRR5266566, SRR5266567, SRR5266568, SRR5364126, SRR5364127, SRR5364128, SRR5364129, SRR5454441, SRR5454442, SRR5454459, SRR5454460, SRR5511204, SRR5511205, SRR5511206, SRR5511207, SRR5558489, SRR5558490, SRR5558491, SRR5558492, SRR5558493, SRR5808857, SRR6048770, SRR6048771, SRR6048794, SRR6048798, SRR6290531, SRR6290532, SRR6290533, SRR6290534, SRR6804604, SRR6804605, SRR7063017, SRR7063018, SRR7063019, SRR7063020, SRR7063021, SRR7063022, SRR7063023, SRR7063024, SRR7071094, SRR7071095, SRR710092, SRR710093, SRR710094, SRR710095, SRR7585372. Chromatin domain hierarchies and compartment scores generated by Calder for all 127 Hi-C maps are available as Supplementary Data 2 in a bed format.

## Code availability

The Calder algorithm is implemented in R and is available on Github [https://github.com/CSOgroup/CALDER] along with a detailed user manual and test dataset. It is also deposited at Zenodo [https://doi.org/10.5281/zenodo.4555593].
