# Supplementary material for: Systematic inference and comparison of multi-scale chromatin sub-compartments connects spatial organization to cell phenotypes
Source: Nat Commun. 2021 May 10;12:2439. doi: 10.1038/s41467-021-22666-3 (PMC8110550; doi:10.1038/s41467-021-22666-3)
Supplement: Supplementary file 1 — Supplementary Information [file 41467_2021_22666_MOESM1_ESM.pdf]

Supplementary Information

Systematic inference and comparison of multi-scale chromatin sub-compartments connects spatial organization to cell phenotypes

Yuanlong Liu[1,2,3], Luca Nanni[4], Stephanie Sungalee[2,5], Marie Zufferey[1,2,3], Daniele Tavernari[1,2,3], Marco Mina[1,2,3], Stefano Ceri[4], Elisa Oricchio[2,5], Giovanni Ciriello[1,2,3,*]

[1] Department of Computational Biology, University of Lausanne, Lausanne, Switzerland
[2] Swiss Cancer Center Leman, Lausanne, Switzerland
[3] Swiss Institute of Bioinformatics, Lausanne, Switzerland
[4] Department of Electronics, Information, and Bioengineering, Politecnico di Milano, Milan, Italy
[5] Swiss Institute for Experimental Cancer Research (ISREC) School of Life Sciences, EPFL

* Correspondence to: giovanni.ciriello@unil.ch

Supplementary Figure 1

a

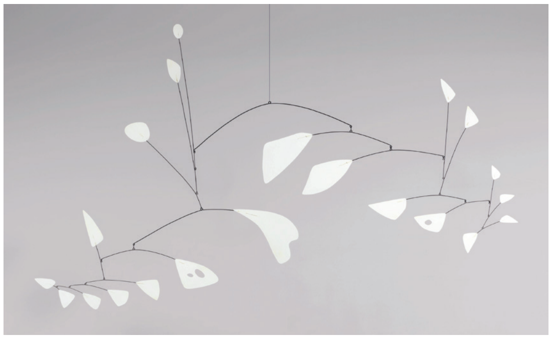

b

GM12878
% of genome having same A/B compartment

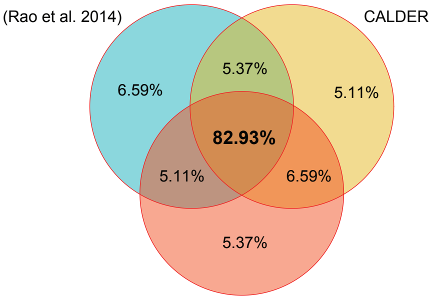

(Lieberman-Aiden et al. 2009)

IMR90
% of genome having same A/B compartment

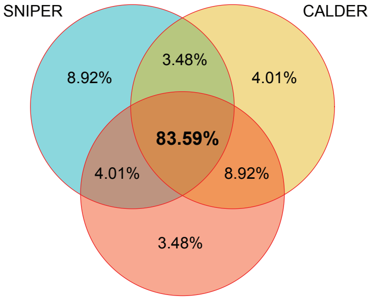

(Liebermann-Aiden et al. 2009)

NHEK
% of genome having same A/B compartment

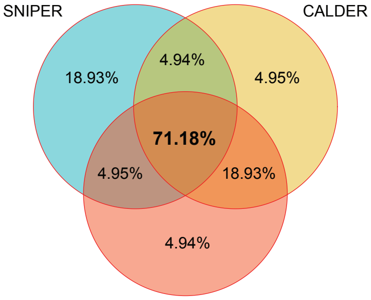

(Liebermann-Aiden et al. 2009)

K562
% of genome having same A/B compartment

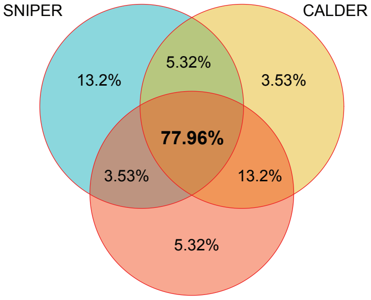

(Liebermann-Aiden et al. 2009)

HUVEC
% of genome having same A/B compartment

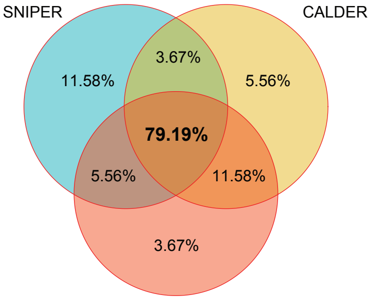

(Liebermann-Aiden et al. 2009)

HMEC
% of genome having same A/B compartment

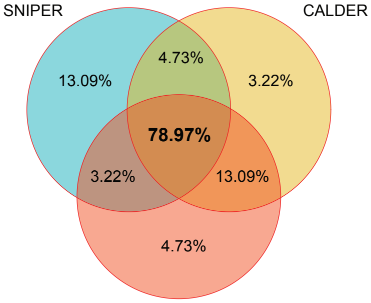

(Liebermann-Aiden et al. 2009)

HeLa
% of genome having same A/B compartment

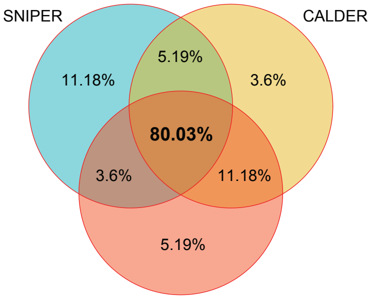

(Liebermann-Aiden et al. 2009)

Supplementary Figure 1. Overlap of A/B compartments called by differenct methods. a) *21 Feuilles Blanches* (1953), Alexandre Calder (1898-1976). **b)** Comparison of A and B compartments called by Calder (yellow), inferred by principal component analysis (Liebermann et al. 2009) (red), and determined by clustering inter-chromosomal interactions either as reported in (Rao et al. 2004) or using SNIPER (blue). Values correspond to the percentage of genomic bins assigned to the same A or B compartment by the different methods.

Supplementary Figure 2

a

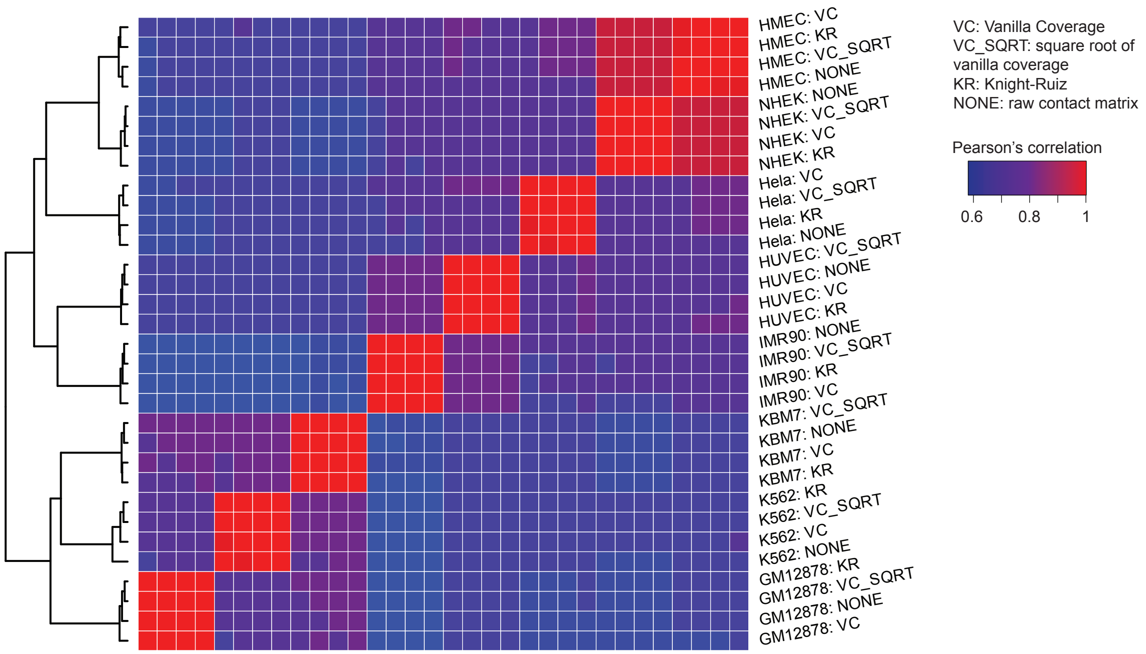

b

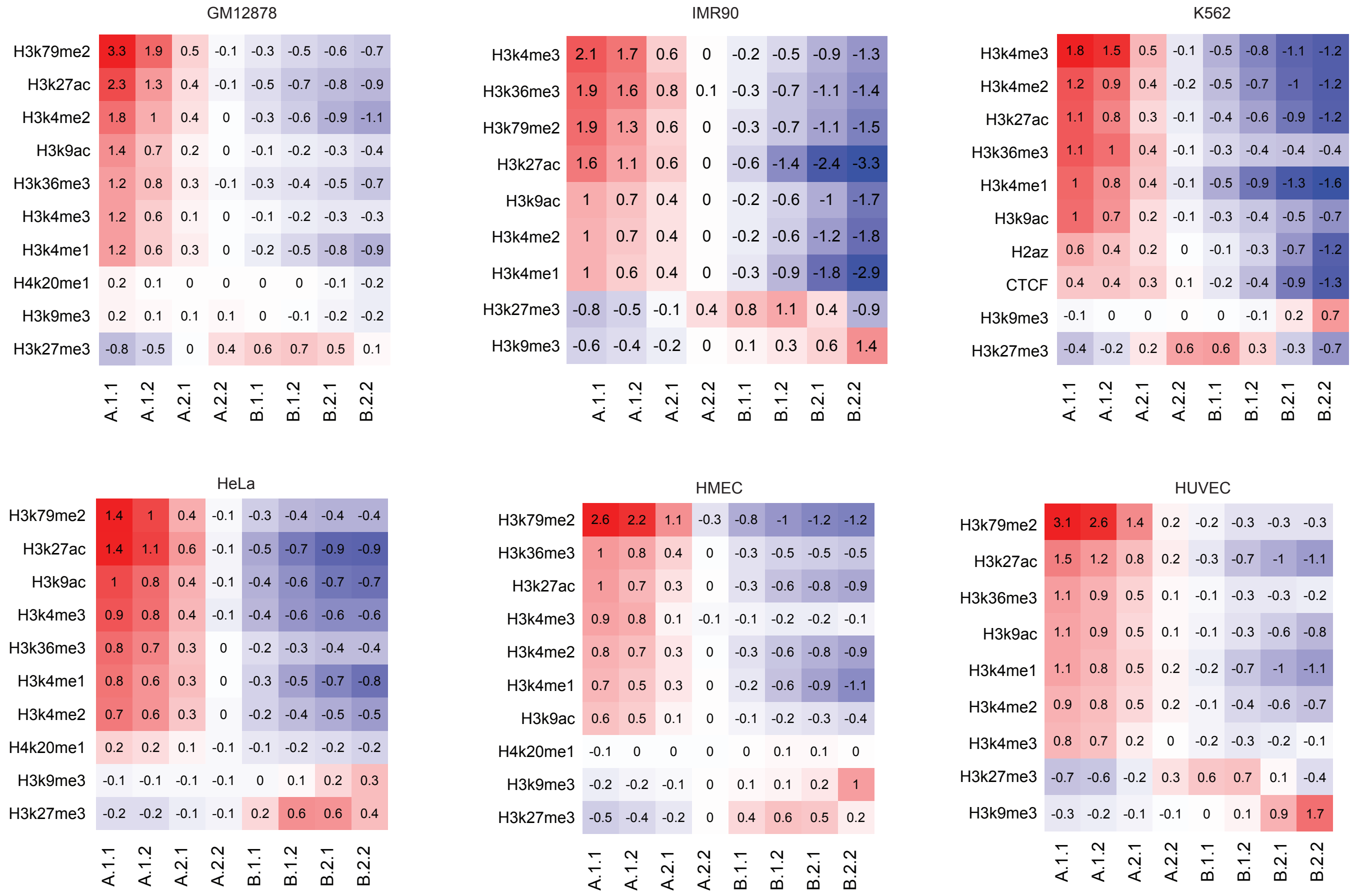

Supplementary Figure 2. Clustering of sub-compartment profiles & enrichment of histone marks in sub-compartments. a) Clustering of sub-compartment profiles inferred from 32 Hi-C contact maps corresponding to 8 cell lines and 4 normalization methods for each cell line map. Clustering was based on Pearson's correlation coefficients between each pair of sub-compartment profiles (8 sub-compartments). **b)** Enrichment of histone marks (rows) in each sub-compartment (columns). Log_2 fold-changes between the median value within a compartment and the overall median value is color coded and reported for 5 cell lines (cell line name reported on the top of the heatmap).

Supplementary Figure 3

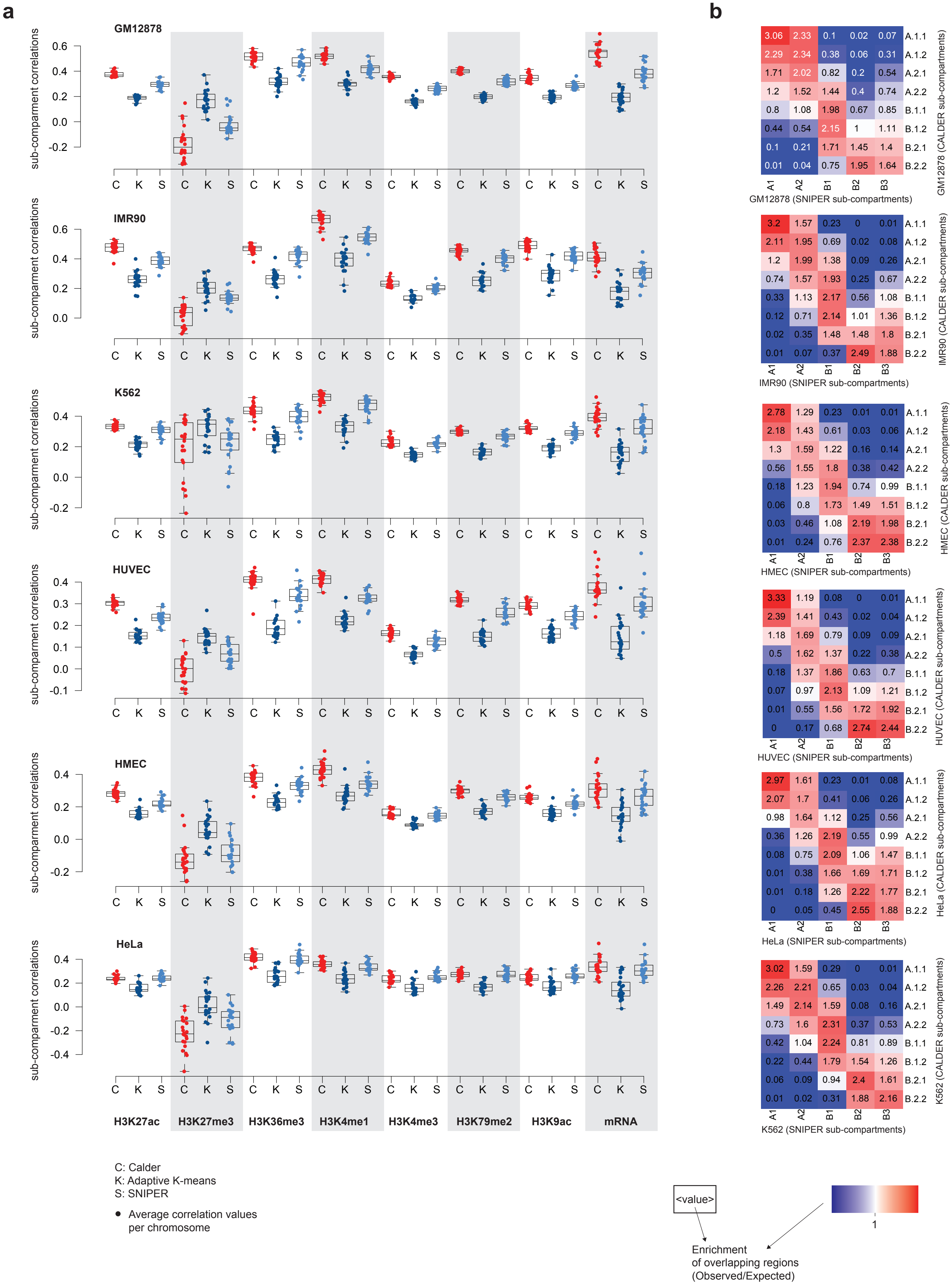

Supplementary Figure 3. Comparison of Calder with two other other methods. a) Correlation between histone mark intensities and sub-compartment assignment inferred by Calder (C, red points), Adaptive K-means (K, dark blue points), and SNIPER (S, light blue points). Boxplots relative to each histone mark are arranged by columns and to each cell line by row. Each data point represents one autosomal chromosome (22 points per box). The bounds of the box in the plot are first quartile (Q_1) and third quartile (Q_3). The lower and upper whiskers are computed by extending the box bounds by 1.5*IQR, where IQR=Q_3−Q_1. **b)** Enrichment (observed/expected) of overlaps between sub-compartments called by SNIPER (columns) and Calder (rows) in 6 cell lines (indicated in the label).

Supplementary Figure 4

a

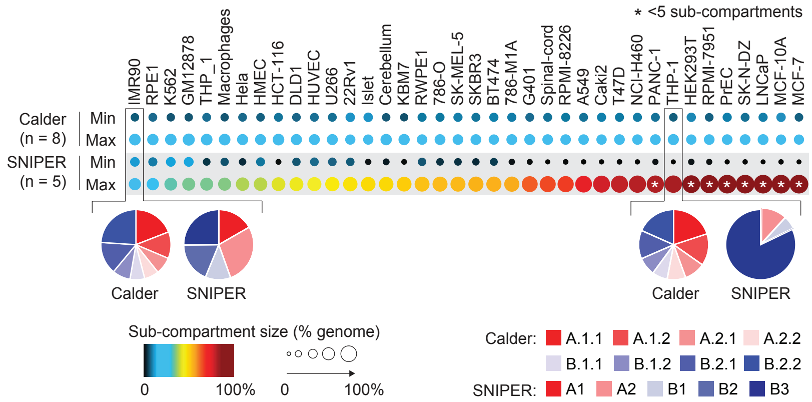

b

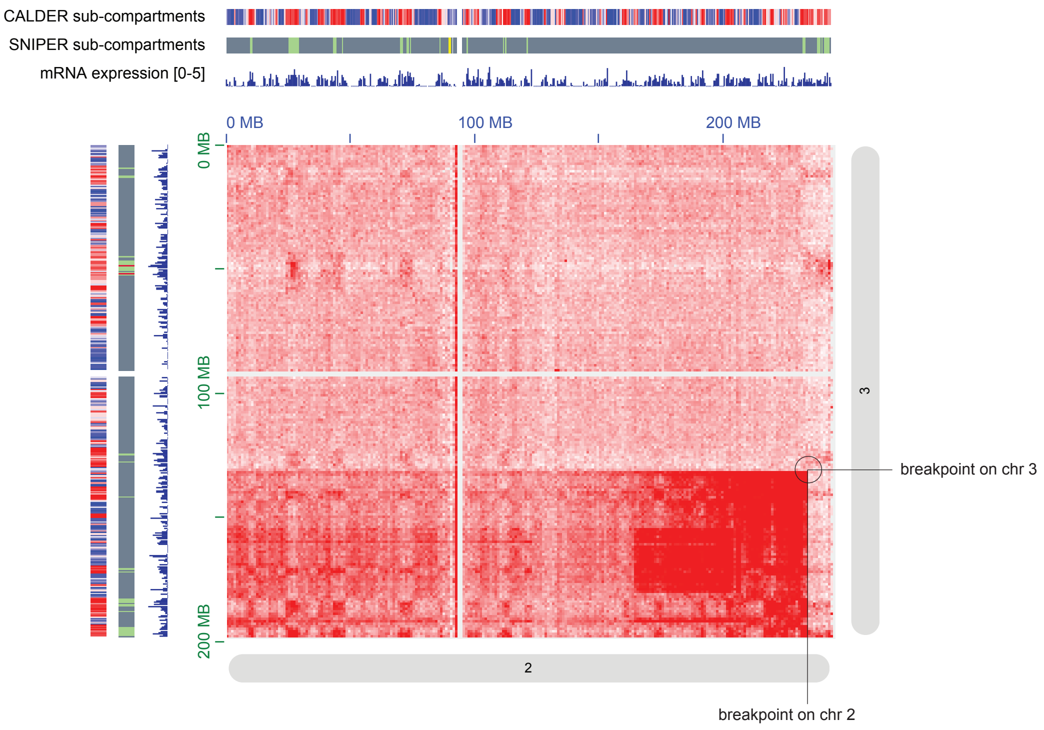

Supplementary Figure 4. Comparison of compartment calling between Calder and SNIPER. a) Minimum and maximum sub-compartment size (measured as % of the genome) identified by Calder (top) and SNIPER (bottom) across 38 cell lines. Zoom-in pie charts of sub-compartment distribution are shown for the cell lines where SNIPER inferred most (IMR90) and least (THP1-macrophages) balanced sub-compartments. **b)** Inter-chromosomal Hi-C contact map (Chr. 2 – Chr. 3) for the renal cancer cell line Caki-2. The dark red corner in the bottom-right area of the map indicates the presence of a chromosomal translocation with a breakpoint corresponding to the vertex of the dark right area (circled in black). Sub-compartment called by Calder and SNIPER and expression values (capped at $\log_2$FPKM=5) of genes in these chromosomes are reported on top and left side of the map.

Supplementary Figure 5

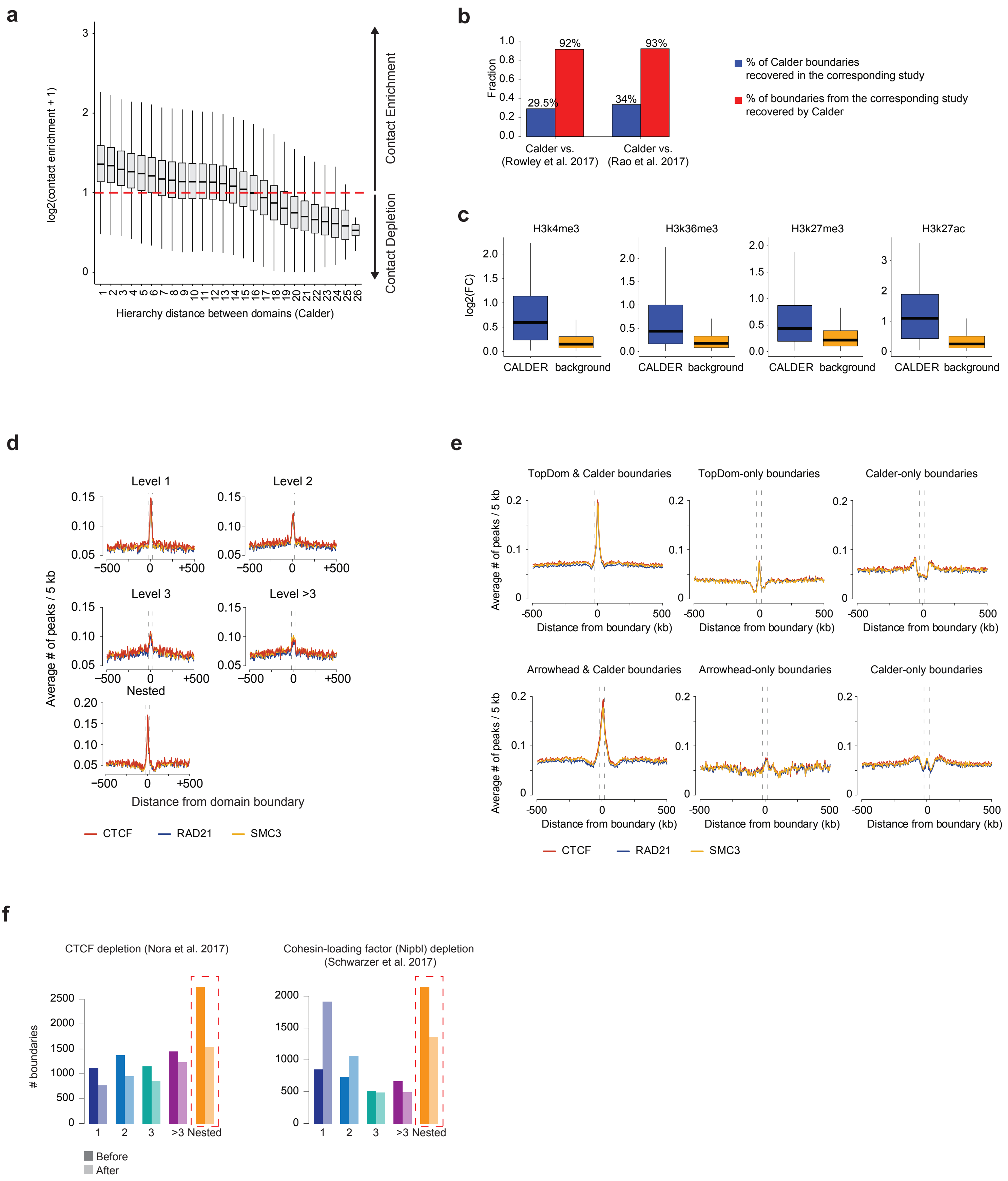

Supplementary Figure 5. Features of compartment domains and boundaries identified by Calder. **a)** Distribution of expression contact enrichment (observed/expected) among compartment domains (Y-axis) based on their distance in the compartment hierarchy inferred by Calder (X-axis). Number of data points for each box from left to right: 7214, 8824, 10468, 14570, 18744, 26336, 36722, 50744, 71792, 99182, 140464, 199336, 283084, 401122, 543456, 675156, 745854, 733164, 631852, 449370, 242752, 91554, 24738, 5066, 640, 40**.** The bounds of the box in the plot are the first quartile (Q_1) and the third quartile (Q_3). The lower and upper whiskers are computed by extending the box bounds by 1.5*IQR, where IQR=Q_3−Q_1**.** **b)** Fraction of compartment boundaries identified by both Calder and either one of the two studies referenced in the figure. The fraction is computed with respect to the total number of boundaries inferred by Calder (blue) or either of the referenced studies (red). **c)** Histone mark intensity fold-change for 4 histone marks (labelled at the top of each boxplot) observed at Calder specific boundaries and expected from the background distribution. P-values are computed by tow-tailed Wilcoxon test. **d)** CTCF, RAD21 and SMC3 ChIP-seq peak signals (average number of peaks in 5-kb intervals) for boundaries inferred by Calder at different levels. **e)** CTCF, RAD21 and SMC3 ChIP-seq peak signals (average number of peaks in 5-kb intervals) for boundaries inferred by both TopDom and Calder (top left), or arrowhead and Calder (bottom left), or exclusively by one of these tools. **f)** Change in number of compartment boundaries (stratified by level) inferred by Calder before and after CTCF depletion (left) and depletion of the cohesin loading factor Nipbl (right).

Supplementary Figure 6

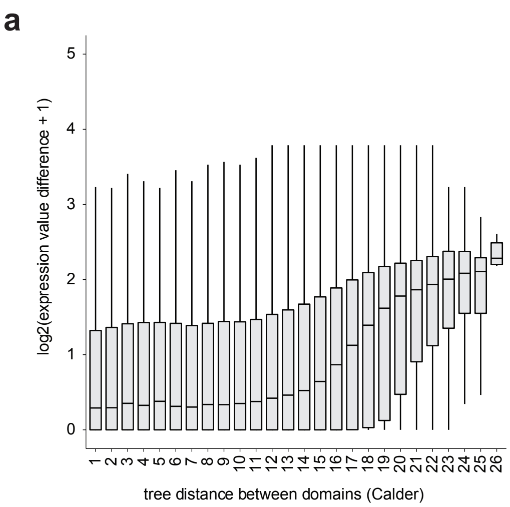

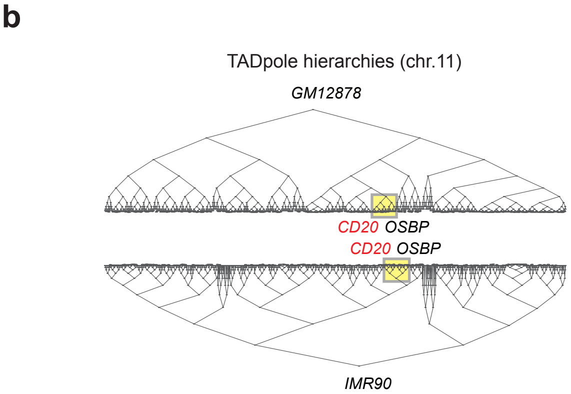

Supplementary Figure 6. **Association of domain distance with gene expression difference & domain hierarchy difference between Calder and TADpole a)** Distribution of expression differences among gene pairs (Y-axis) based on the distance of their corresponding compartment domains (X-axis). Number of data points for each box is the same as in Supp. Fig 5a. The bounds of the box in the plot are the first quartile (Q_1) and the third quartile (Q_3). The lower and upper whiskers are computed by extending the box bounds by 1.5*IQR, where $IQR=Q_3-Q_1$. **b)** domain hierarchy inferred by TADpole for IMR90 (left) and GM12878 (right) cell lines. The domains containing *MS4A1* (a.k.a. *CD20*) and *OSBP* are highlighted.

Supplementary Figure 7

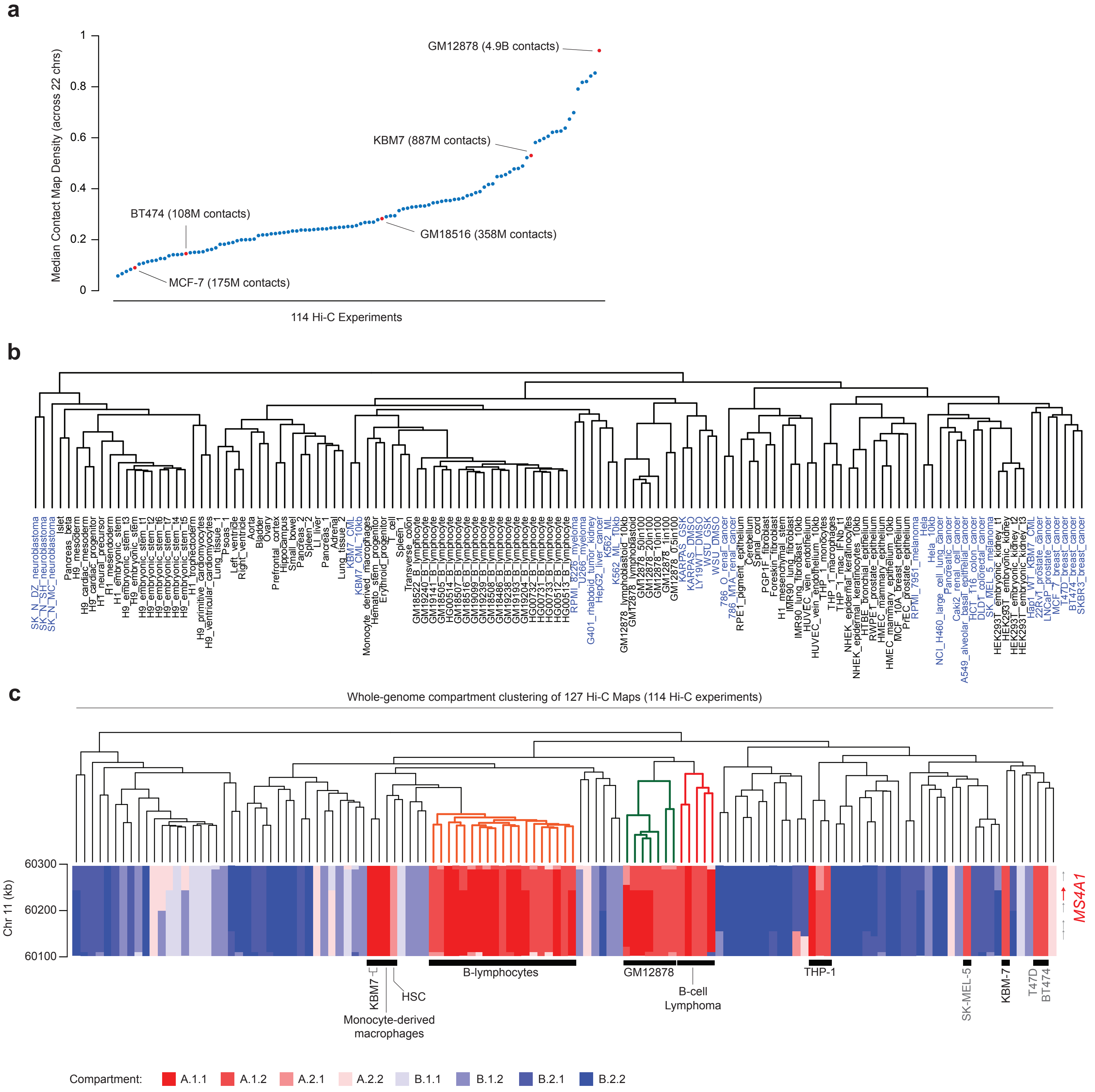

Supplementary Figure 7. Applying Calder to 127 Hi-C maps. a) Median contact map density across 22 chromosomes (Y-axis) of 114 Hi-C experiments (X-axis). The corresponding total number of contacts are shown for GM12878, KBM7, GM18516, BT474 and MCF-7. **b)** Clustering of 127 Hi-C maps based on whole-genome bin compartment annotation. Hi-C dataset labels are shown for all Hi-C dataset including normal (black font) and cancer (blue font) cell lines. **c)** Compartment annotation of the *MS4A1* (CD20) locus for all Hi-C datasets clustered as in panel (b) and as in Fig. 3a.

Supplementary Figure 8

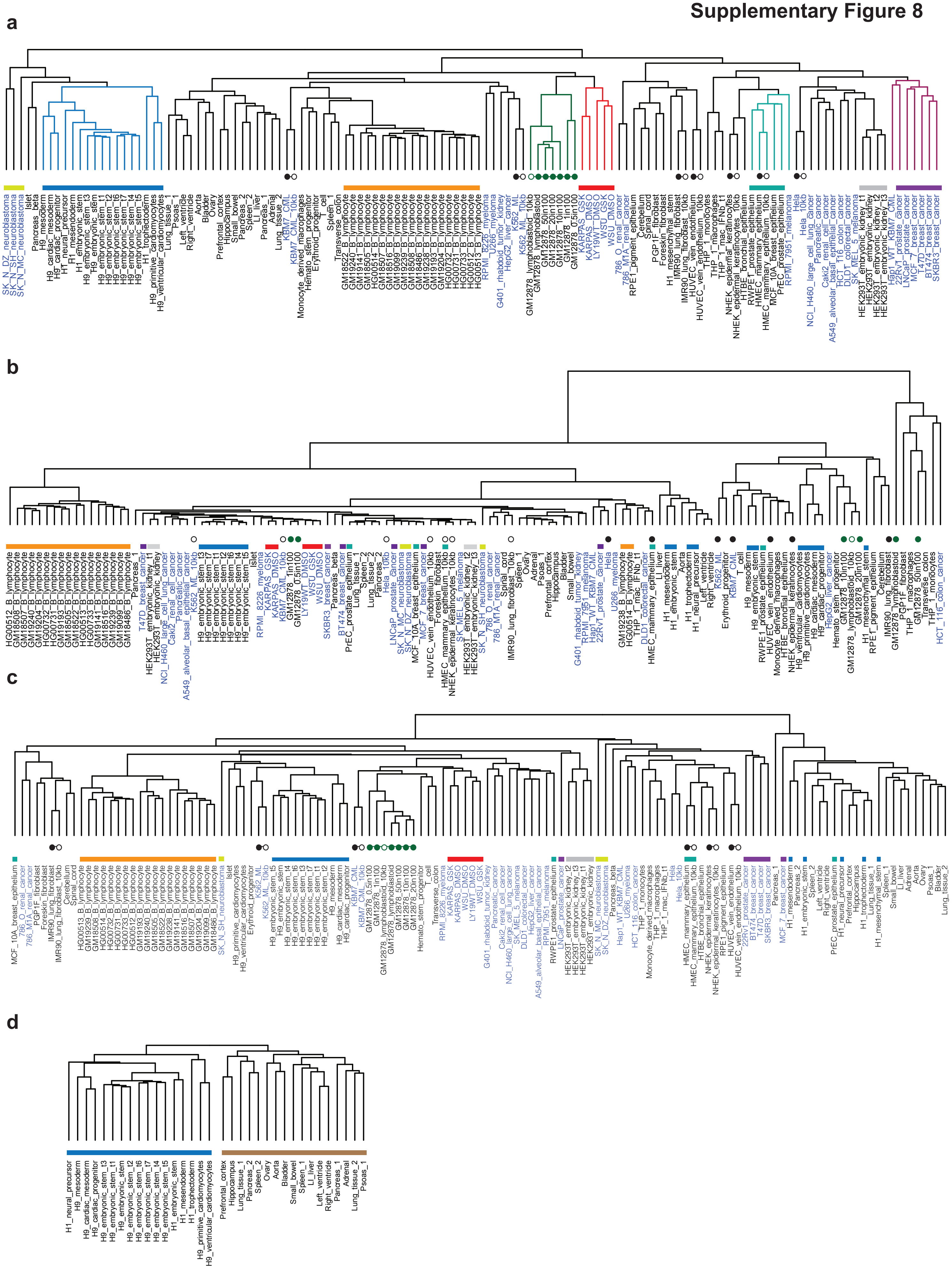

Supplementary Figure 8. Clustering of Hi-C maps. a) Clustering of 127 Hi-C maps based on bin sub-compartment annotations inferred by Calder. **b)** Clustering of 127 Hi-C maps based on the first principal component values of the observed/expected contact matrix for each bin.**c)** Clustering of 127 Hi-C maps based on the sign of the first principal component values of the observed/expected contact matrix for each bin.Hi-C dataset labels are shown for all Hi-C dataset including normal (black font) and cancer (blue font) cell lines. **d)** Clustering of Hi-C maps derived from stem-like cell lines (blue cluster) and differentiated tissues (brown cluster) based on sub-compartment annotations of genomic regions found in the same A (or B) compartment across all cell lines.

Supplementary Figure 9

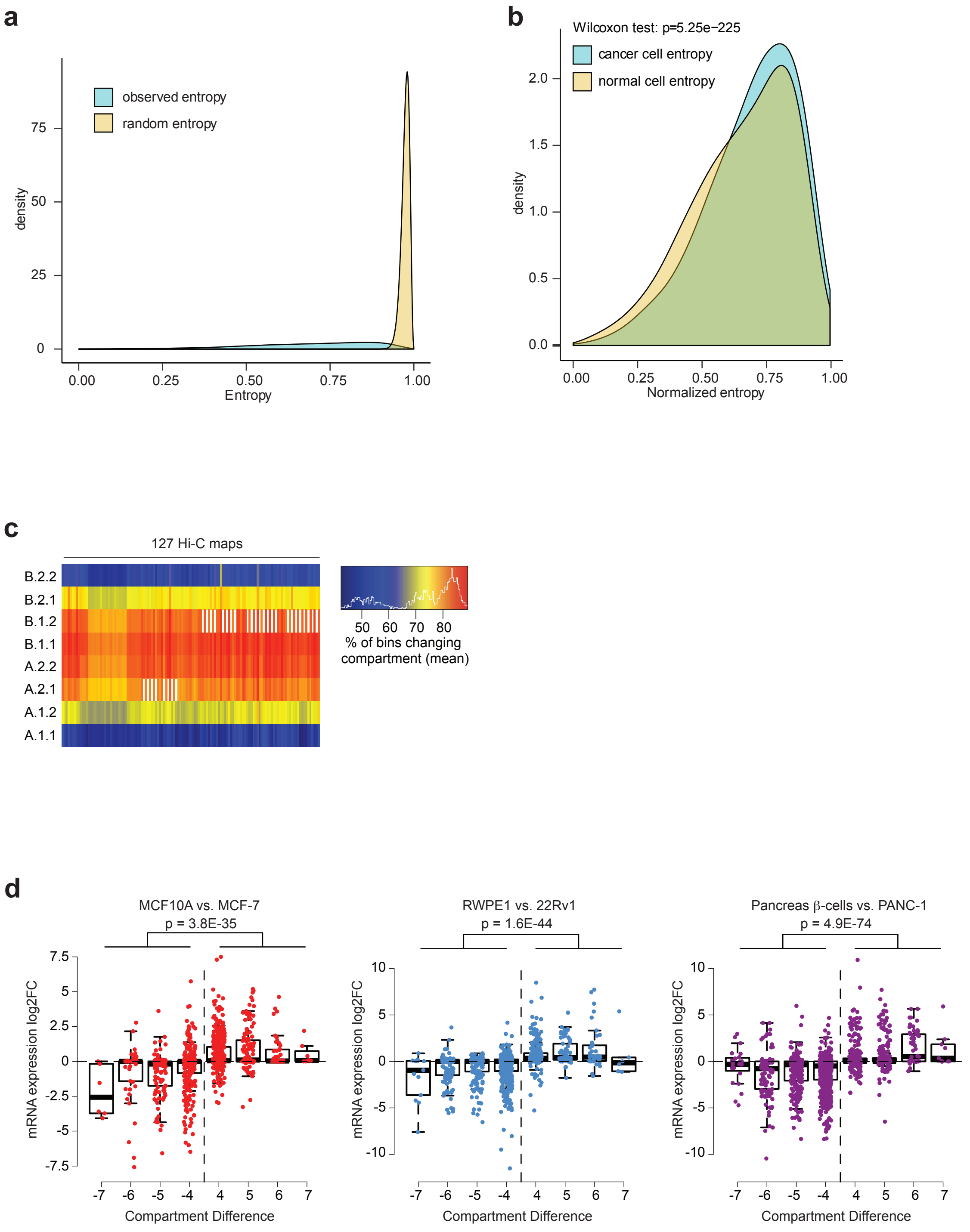

Supplementary Figure 9. Comparison of compartment profile between datasets. a) Density distribution of the observed chromatin entropy (light blue) and chromatin entropy after randomization of bin compartment labels (yellow). **b)** Density distribution of chromatin entropy in cancer (light blue) and normal (yellow) cell lines. **c)** Percentage of bins (color coded in heatmap) that had different compartment in at least one cell line among the 127 analyzed (X-axis) within each compartment (Y-axis). **d)** mRNA expression change (log2 fold-change of FPKM normalized RNA-seq reads) of genes having at least 4 compartment difference of between normal and cancer cells derived from breast (left), prostate (center), and pancreatic (right) tissues.

Supplementary figure 10

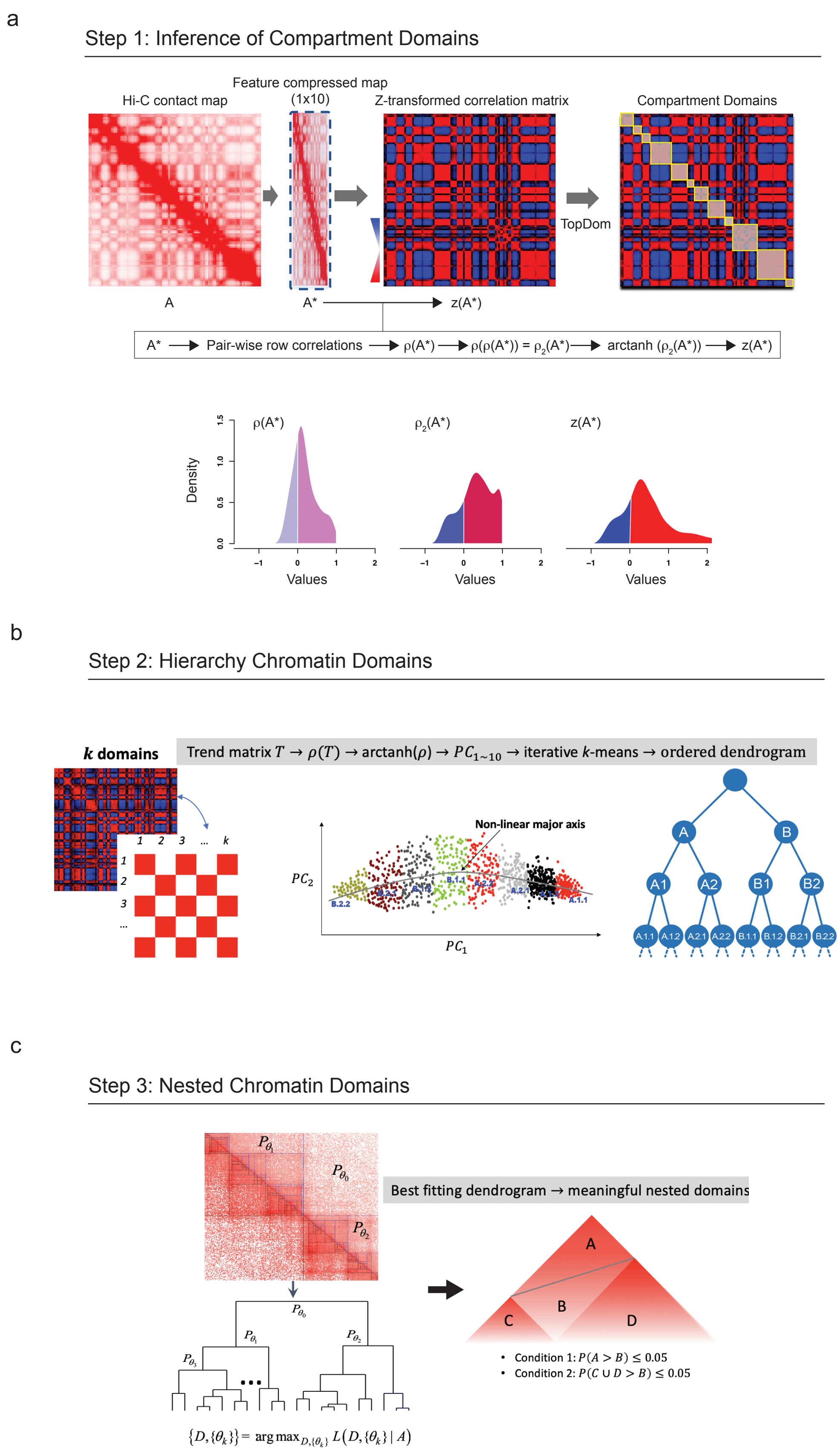

Supplementary Figure 10. Schematic representation of the Calder algorithm. a) First, Hi-C contact maps are compressed along one dimension to increase the bin size and number of contacts per bin. Bin pairwise correlations are computed and transformed by Fisher's Z-transformation. Compartment domains are inferred using the approach implemented in TopDom applied to the Z-transformed correlation matrix z(A*). b) Next, a trend matrix is built from z(A*). Compartment domains are clustered using a divisive hierarchical clustering approach based on K-means and exclusively based on their inter-domain contacts estimated from the first 10 principal components of the trend matrix. c) Last, a mixture log-normal distribution model can be applied to short-range contacts within each compartment domain to estimate the likelihood of nested sub-domains. (A detailed description of the algorithm is provided in **Methods**).
