# Supplementary material for: Systematic inference and comparison of multi-scale chromatin sub-compartments connects spatial organization to cell phenotypes
Source: Nat Commun. 2021 May 10;12:2439. doi: 10.1038/s41467-021-22666-3 (PMC8110550; doi:10.1038/s41467-021-22666-3)
Supplement: Supplementary file 3 — Description of Additional Supplementary Files [file 41467_2021_22666_MOESM3_ESM.pdf]

Description of Additional Supplementary Files

File Name: Supplementary Data 1
Description: IDs and links to Hi-C, ChIP-seq, and RNA-seq datasets used in this study

File Name: Supplementary Data 2
Description: Complete domain hierarchies inferred by CALDER from 127 Hi-C contact maps

File Name: Supplementary Data 3
Description: Percentage of the genome assigned to each sub-compartment by Calder and SNIPER for 38 Hi-C datasets.

File Name: Supplementary Data 4
Description: Correlation between sub-compartment calls and gene expression for SNIPER and CALDER

File Name: Supplementary Data 5
Description: Matrix of sub-compartment domain assignments for 127 Hi-C contact maps used for the clustering analysis in Figure 3

File Name: Supplementary Data 6
Description: Gene set enrichment analysis (mSigDB) results for high entropy ($S>0.95$) and low entropy ($S<0.15$) genes

File Name: Supplementary Data 7
Description: Repositioned compartment domains between normal and cancer cell lines derived from breast, prostate, and pancreatic tissue samples
