# Supplementary material for: Systematic inference and comparison of multi-scale chromatin sub-compartments connects spatial organization to cell phenotypes
Source: Nat Commun. 2021 May 10;12:2439. doi: 10.1038/s41467-021-22666-3 (PMC8110550; doi:10.1038/s41467-021-22666-3)
Supplement: Supplementary file 7 — Supplementary Dataset 4 [file 41467_2021_22666_MOESM7_ESM.pdf]

**(SNIPER**

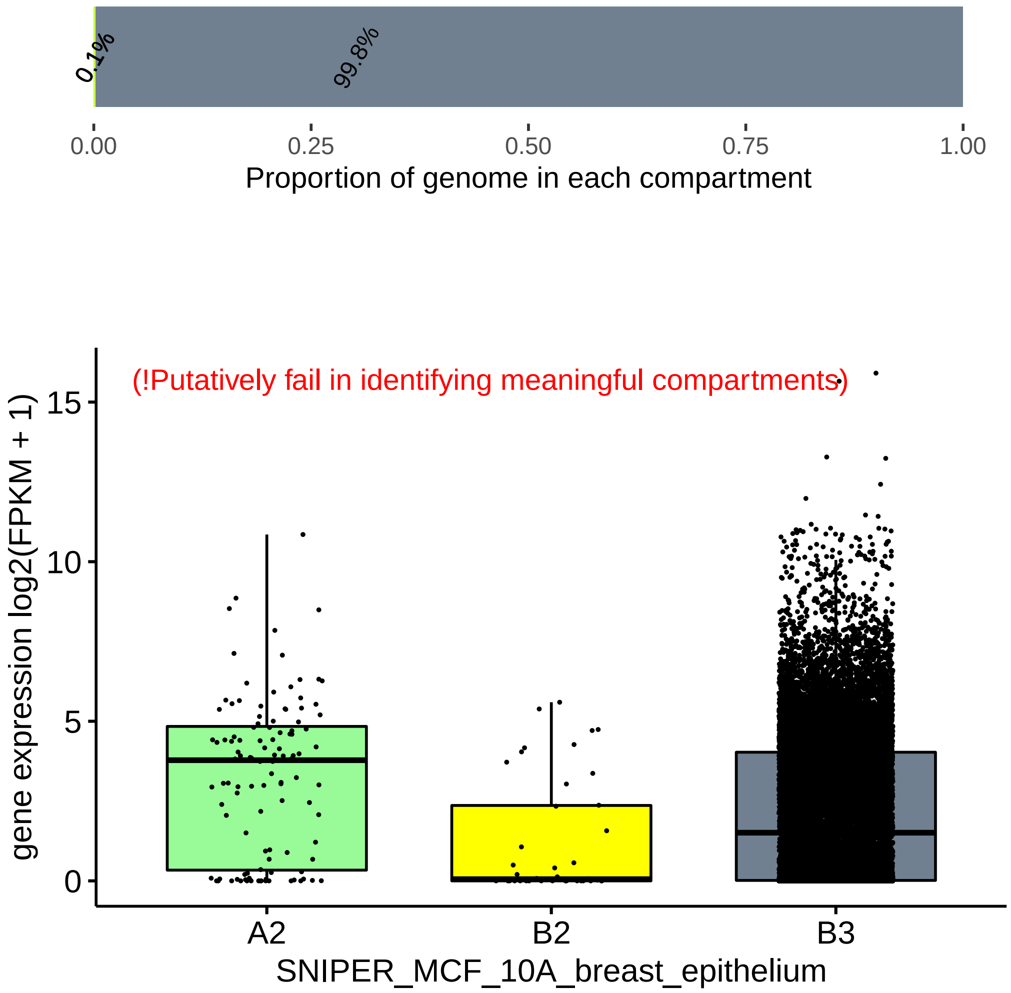


**(CALDER**

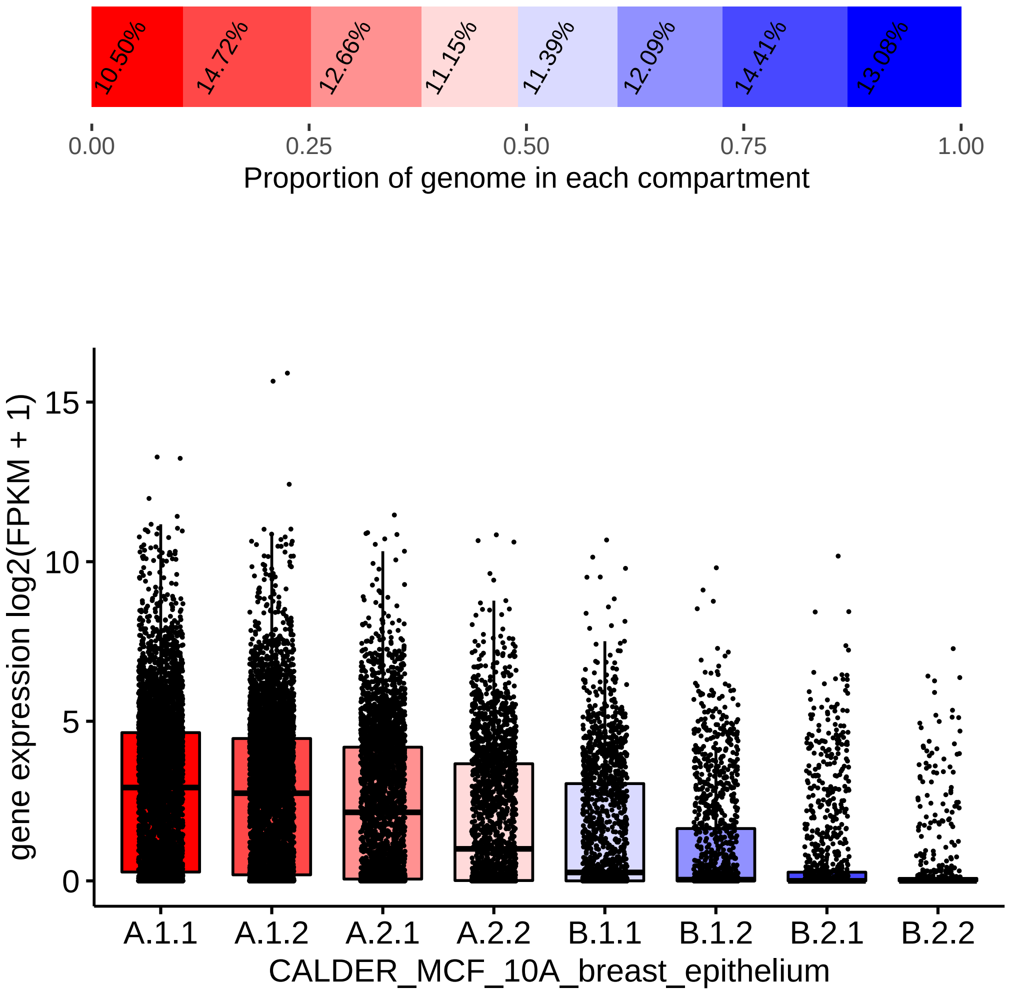

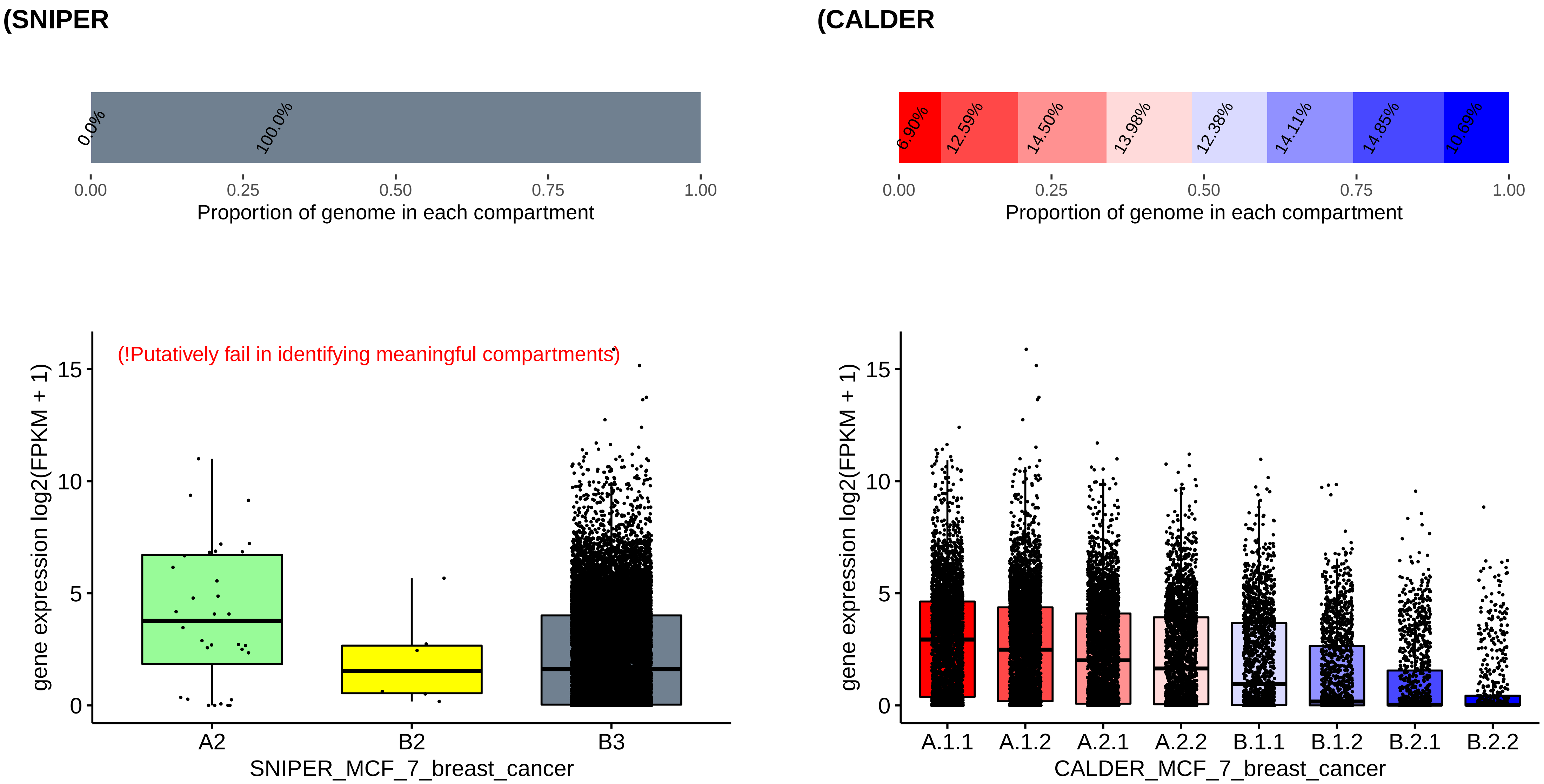
(SNIPER
0.0%
100.0%
0.00
0.25
0.50
0.75
1.00
Proportion of genome in each compartment
(!Putatively fail in identifying meaningful compartments)
gene expression log2(FPKM + 1)
0
5
10
15
A2
B2
B3
SNIPER_MCF_7_breast_cancer
(CALDER
6.90%
12.59%
14.50%
13.98%
12.38%
14.11%
14.85%
10.69%
0.00
0.25
0.50
0.75
1.00
Proportion of genome in each compartment
gene expression log2(FPKM + 1)
0
5
10
15
A.1.1
A.1.2
A.2.1
A.2.2
B.1.1
B.1.2
B.2.1
B.2.2
CALDER_MCF_7_breast_cancer

(SNIPER

(CALDER

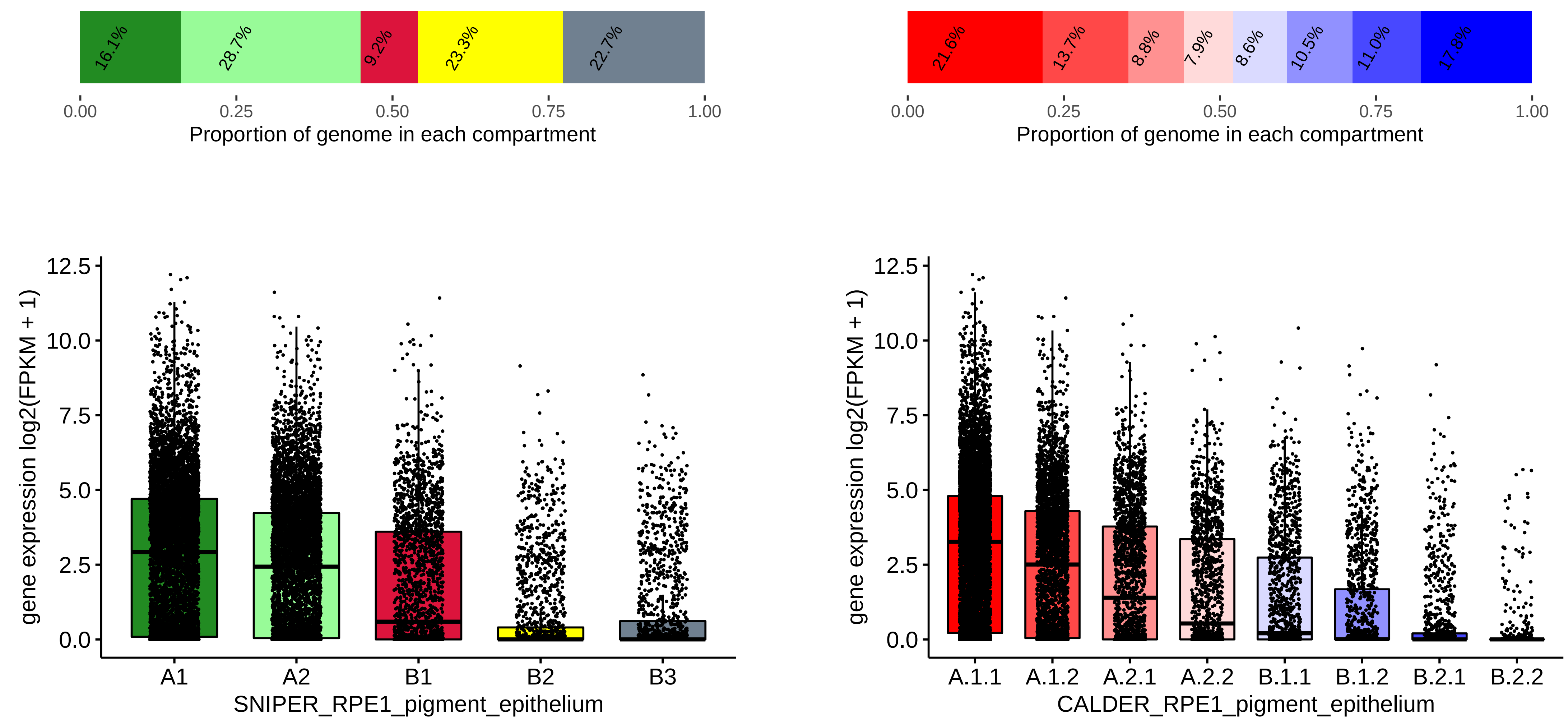

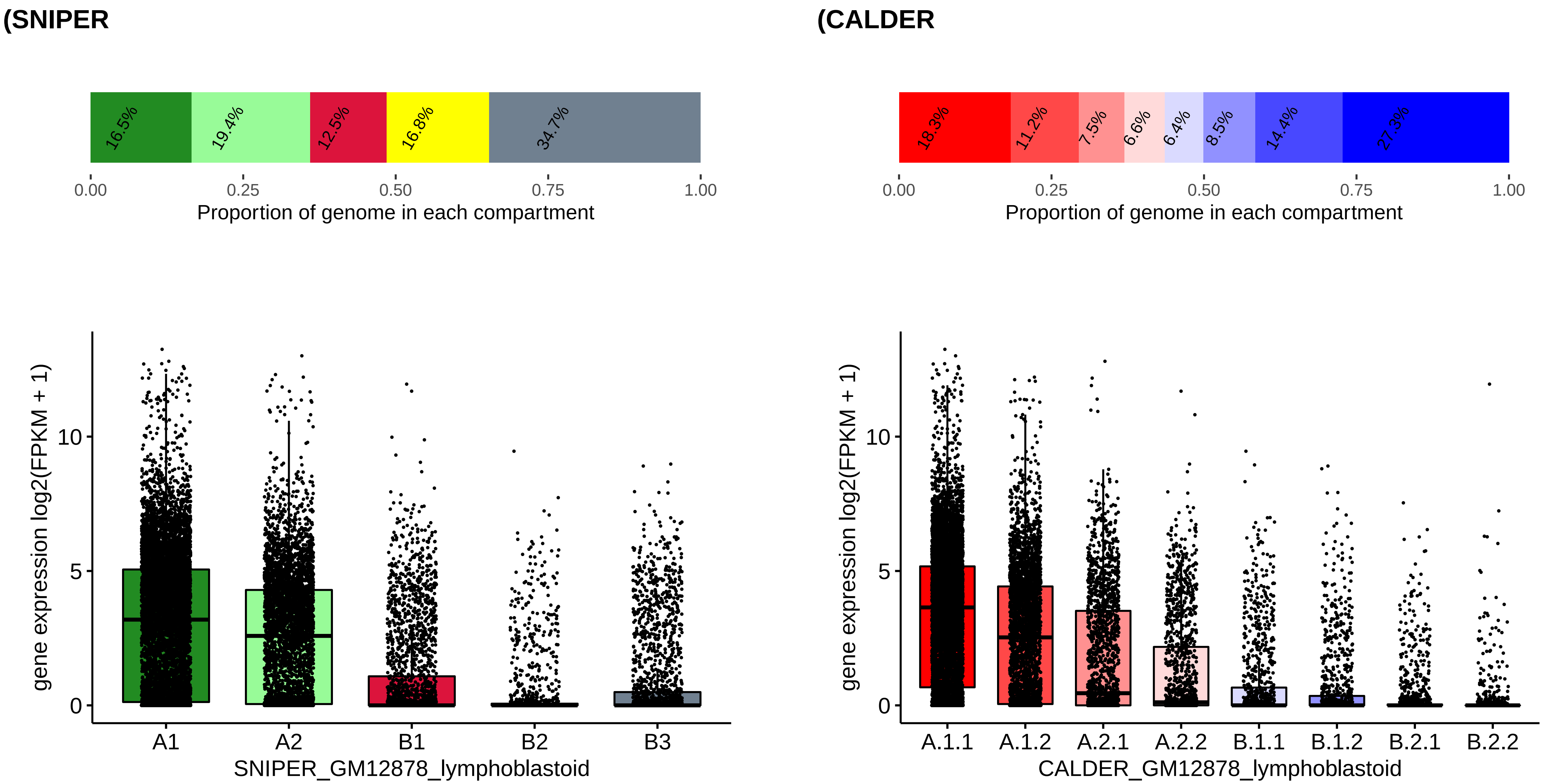
(SNIPER
(CALDER
16.5%
19.4%
12.5%
16.8%
34.7%
18.3%
11.2%
7.5%
6.6%
6.4%
8.5%
14.4%
27.3%
0.00
0.25
0.50
0.75
1.00
Proportion of genome in each compartment
gene expression log2(FPKM + 1)
0
5
10
A1
A2
B1
B2
B3
SNIPER_GM12878_lymphoblastoid
A.1.1
A.1.2
A.2.1
A.2.2
B.1.1
B.1.2
B.2.1
B.2.2
CALDER_GM12878_lymphoblastoid

(SNIPER

(CALDER

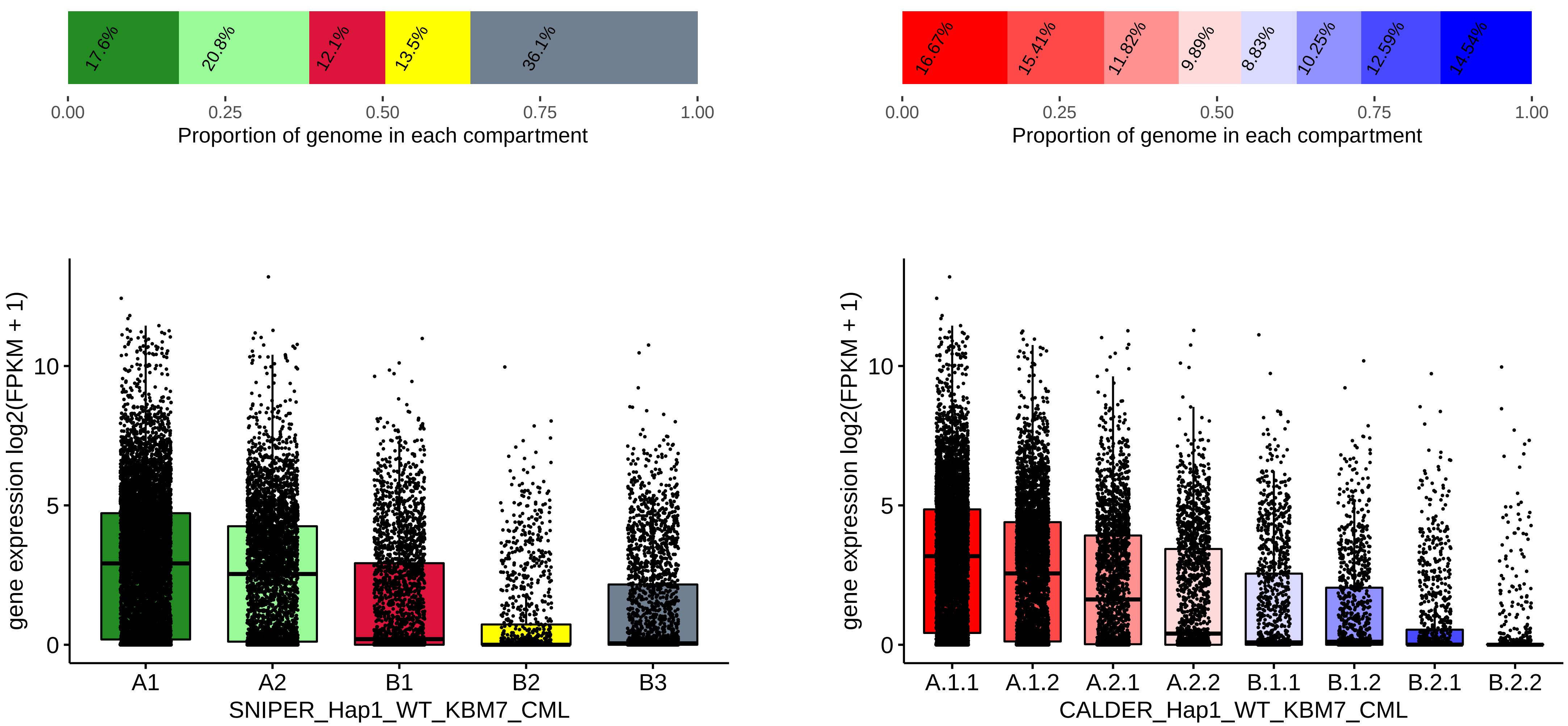

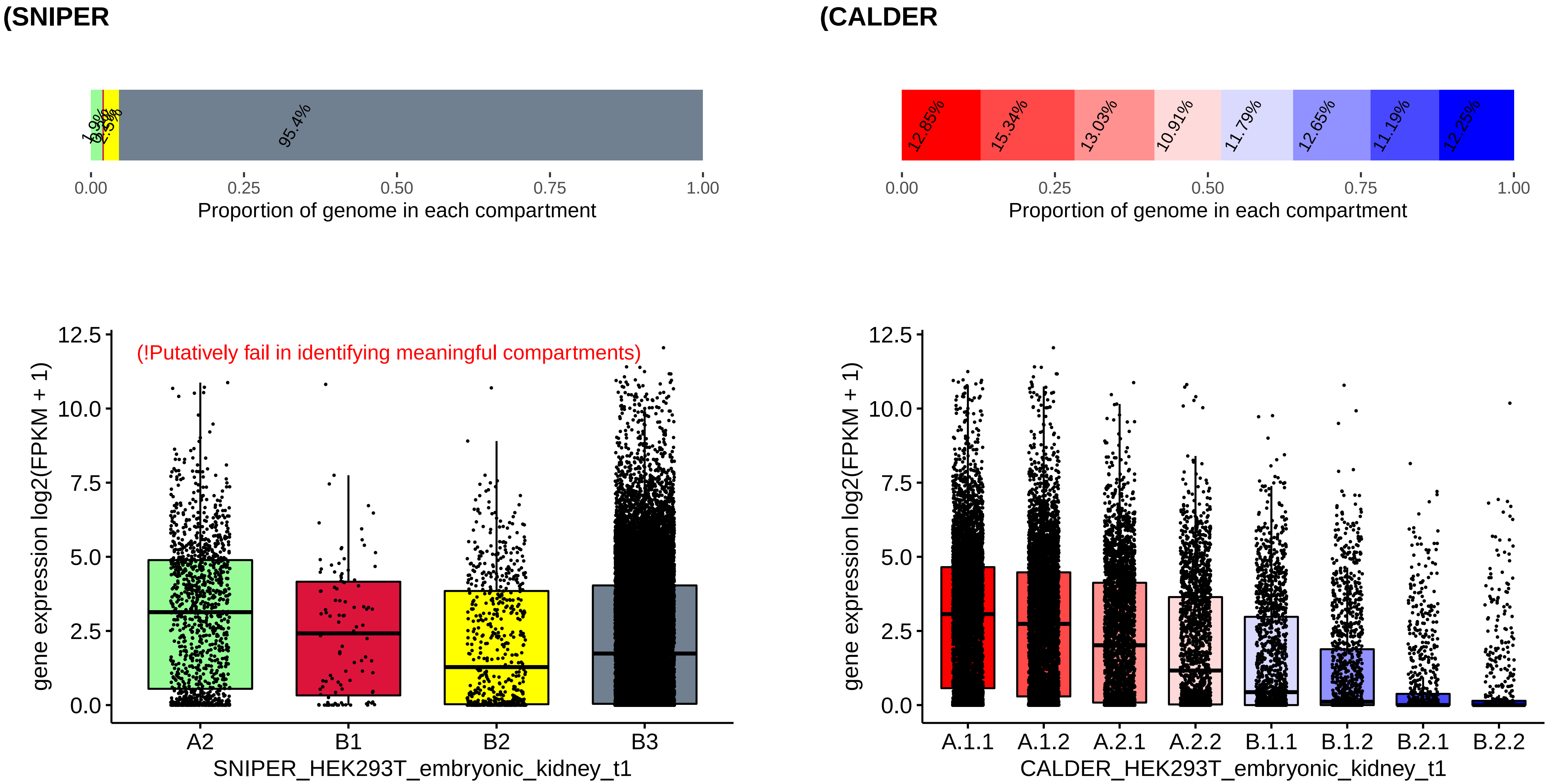

(SNIPER
95.4%
0.00
0.25
0.50
0.75
1.00
Proportion of genome in each compartment
(!Putatively fail in identifying meaningful compartments)
gene expression log2(FPKM + 1)
12.5
10.0
7.5
5.0
2.5
0.0
A2
B1
B2
B3
SNIPER_HEK293T_embryonic_kidney_t1
(CALDER
12.85%
15.34%
13.03%
10.91%
11.79%
12.65%
11.19%
12.25%
0.00
0.25
0.50
0.75
1.00
Proportion of genome in each compartment
gene expression log2(FPKM + 1)
12.5
10.0
7.5
5.0
2.5
0.0
A.1.1
A.1.2
A.2.1
A.2.2
B.1.1
B.1.2
B.2.1
B.2.2
CALDER_HEK293T_embryonic_kidney_t1

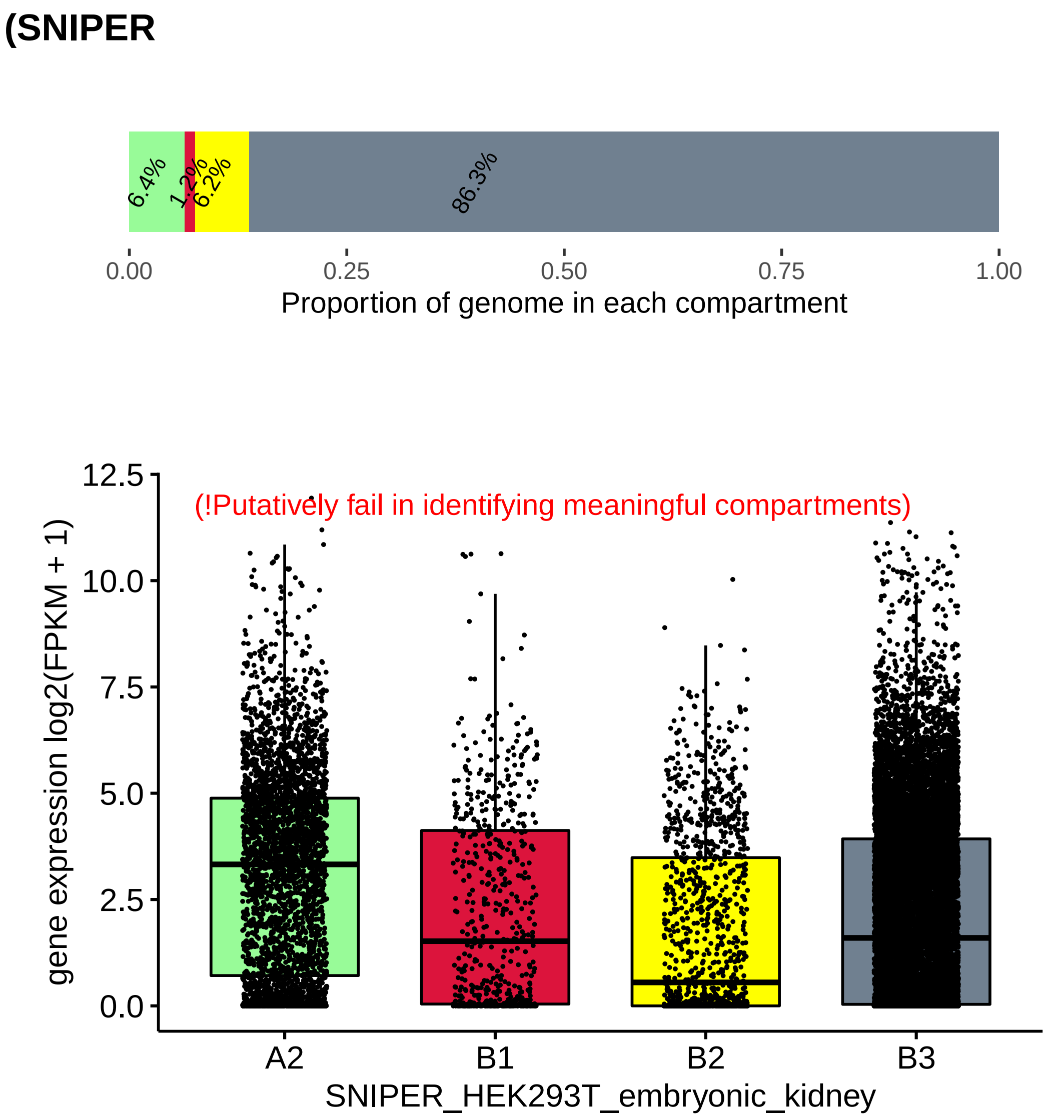
(SNIPER
6.4%
1.2%
6.2%
86.3%
0.00
0.25
0.50
0.75
1.00
Proportion of genome in each compartment
(!Putatively fail in identifying meaningful compartments)
12.5
10.0
7.5
5.0
2.5
0.0
gene expression log2(FPKM + 1)
A2
B1
B2
B3
SNIPER_HEK293T_embryonic_kidney

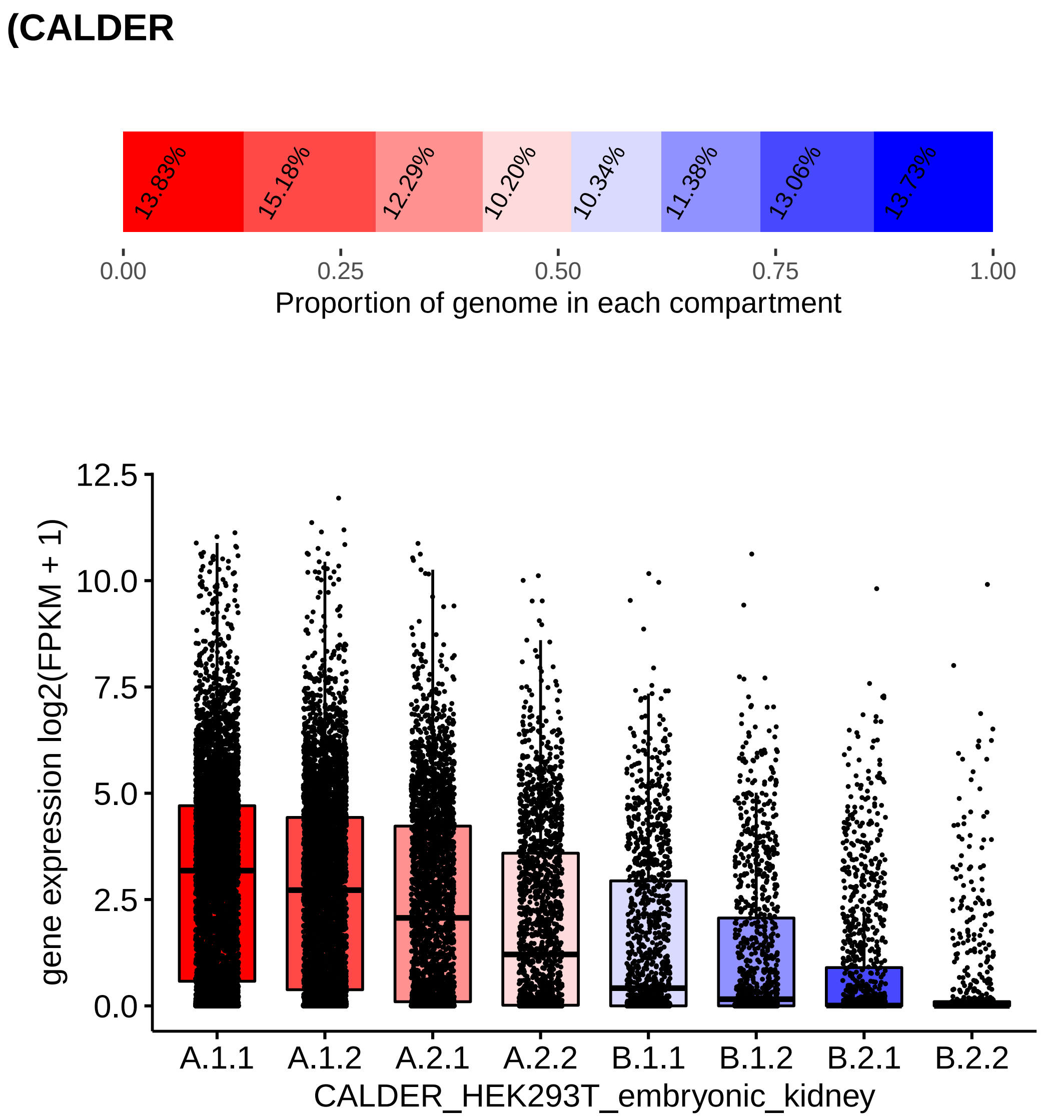
(CALDER
13.83%
15.18%
12.29%
10.20%
10.34%
11.38%
13.06%
13.73%
0.00
0.25
0.50
0.75
1.00
Proportion of genome in each compartment
12.5
10.0
7.5
5.0
2.5
0.0
gene expression log2(FPKM + 1)
A.1.1
A.1.2
A.2.1
A.2.2
B.1.1
B.1.2
B.2.1
B.2.2
CALDER_HEK293T_embryonic_kidney

(SNIPER

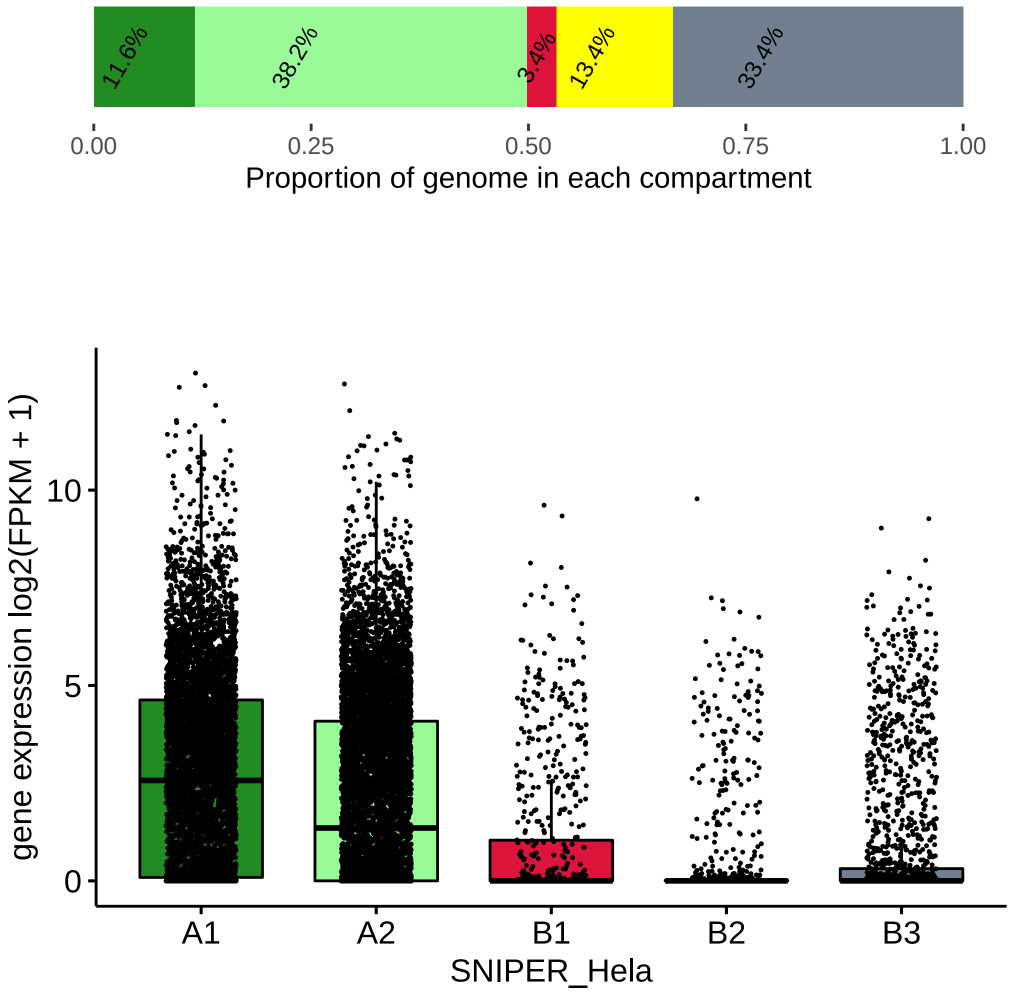


(CALDER

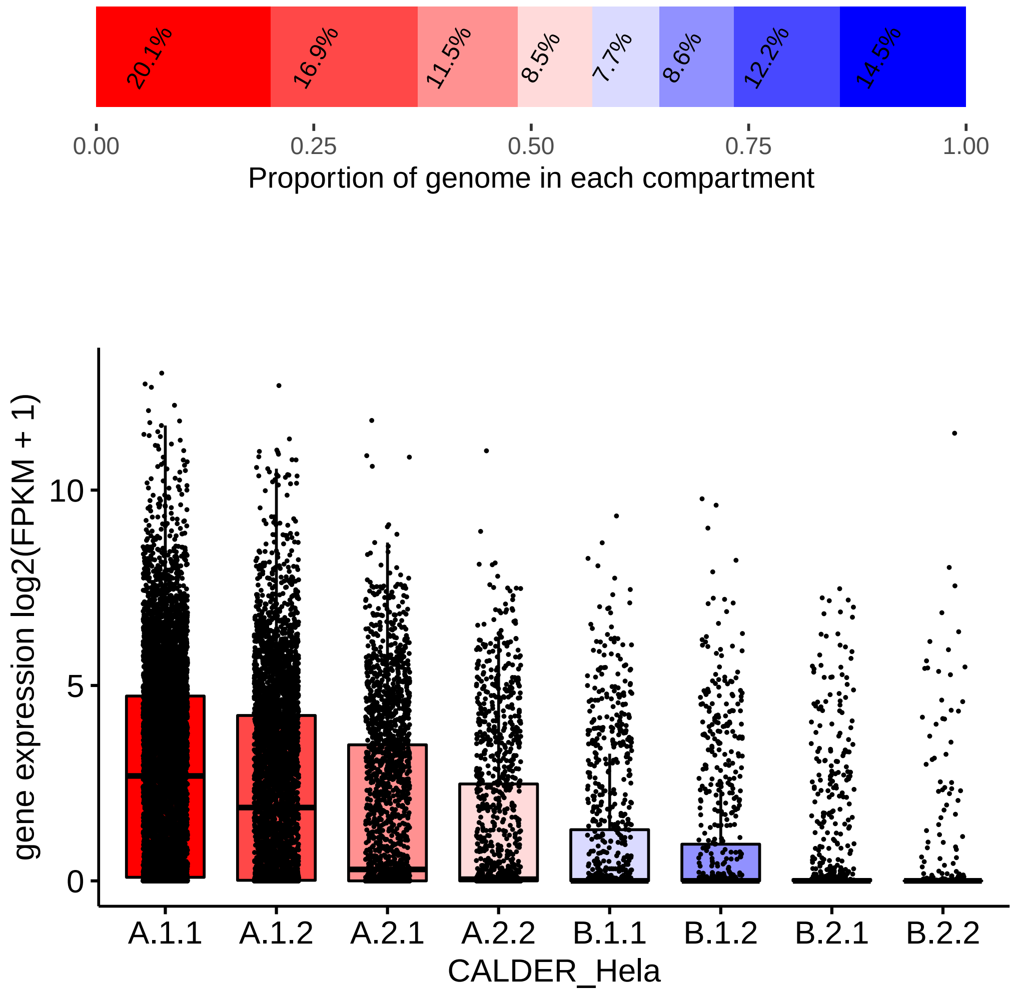

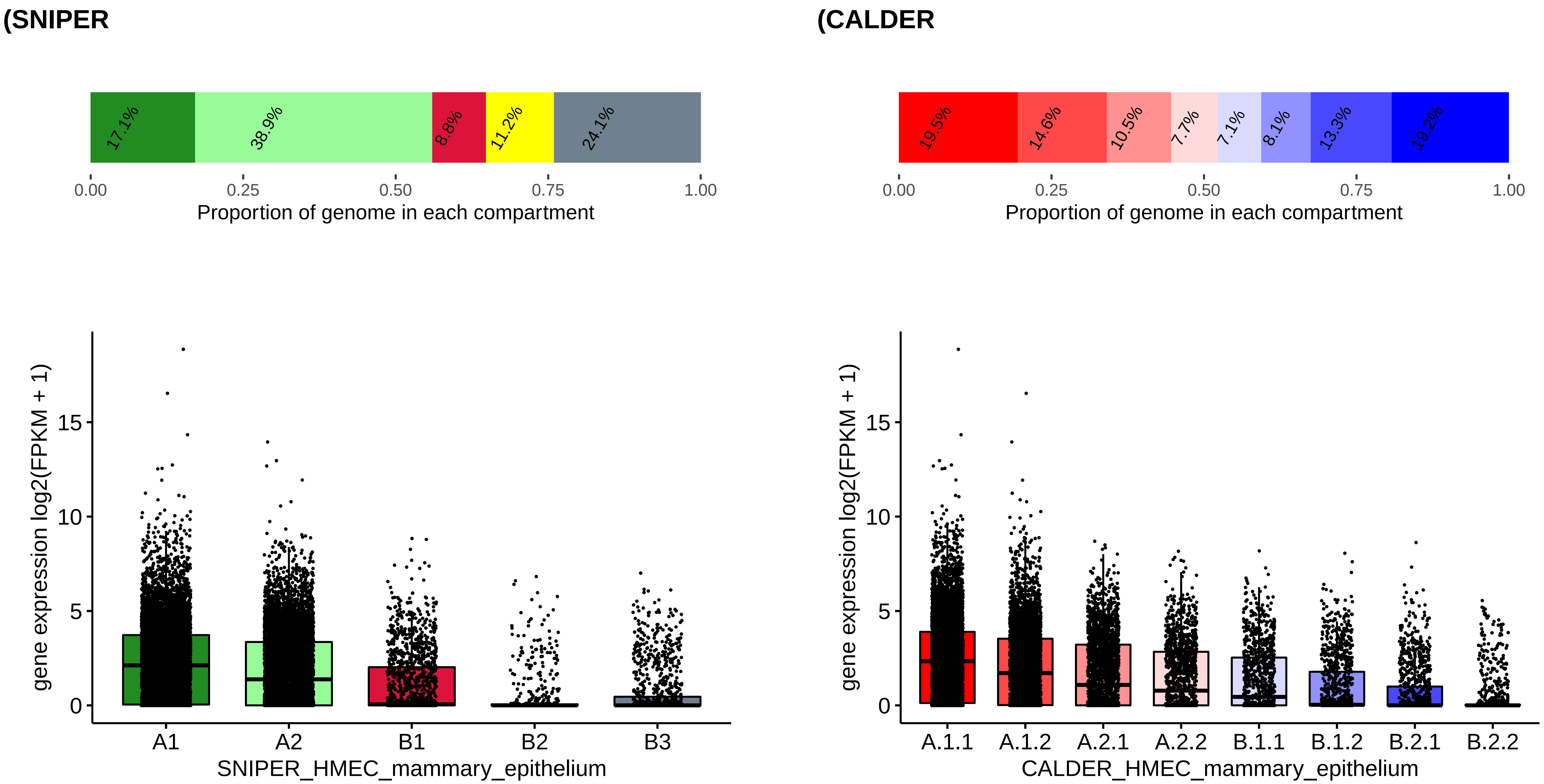

(SNIPER
(CALDER
17.1%
38.9%
8.8%
11.2%
24.1%
0.00
0.25
0.50
0.75
1.00
Proportion of genome in each compartment
19.5%
14.6%
10.5%
7.7%
7.1%
8.1%
13.3%
19.2%
0.00
0.25
0.50
0.75
1.00
Proportion of genome in each compartment
gene expression log2(FPKM + 1)
0
5
10
15
A1
A2
B1
B2
B3
SNIPER_HMEC_mammary_epithelium
gene expression log2(FPKM + 1)
0
5
10
15
A.1.1
A.1.2
A.2.1
A.2.2
B.1.1
B.1.2
B.2.1
B.2.2
CALDER_HMEC_mammary_epithelium

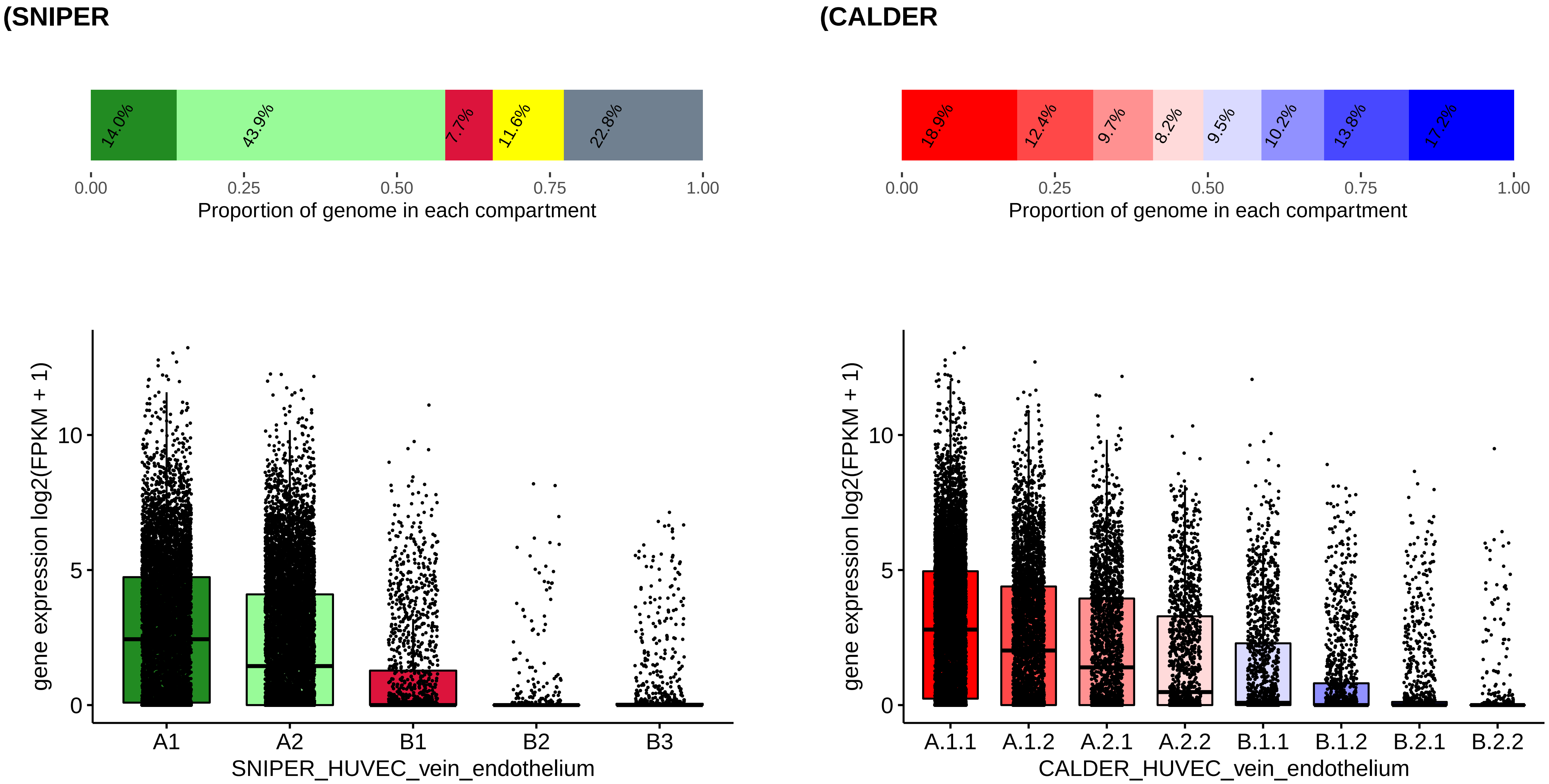
(SNIPER
(CALDER
14.0%
43.9%
7.7%
11.6%
22.8%
0.00
0.25
0.50
0.75
1.00
Proportion of genome in each compartment
18.9%
12.4%
9.7%
8.2%
9.5%
10.2%
13.8%
17.2%
Proportion of genome in each compartment
gene expression log2(FPKM + 1)
0
5
10
A1
A2
B1
B2
B3
SNIPER_HUVEC_vein_endothelium
A.1.1
A.1.2
A.2.1
A.2.2
B.1.1
B.1.2
B.2.1
B.2.2
CALDER_HUVEC_vein_endothelium

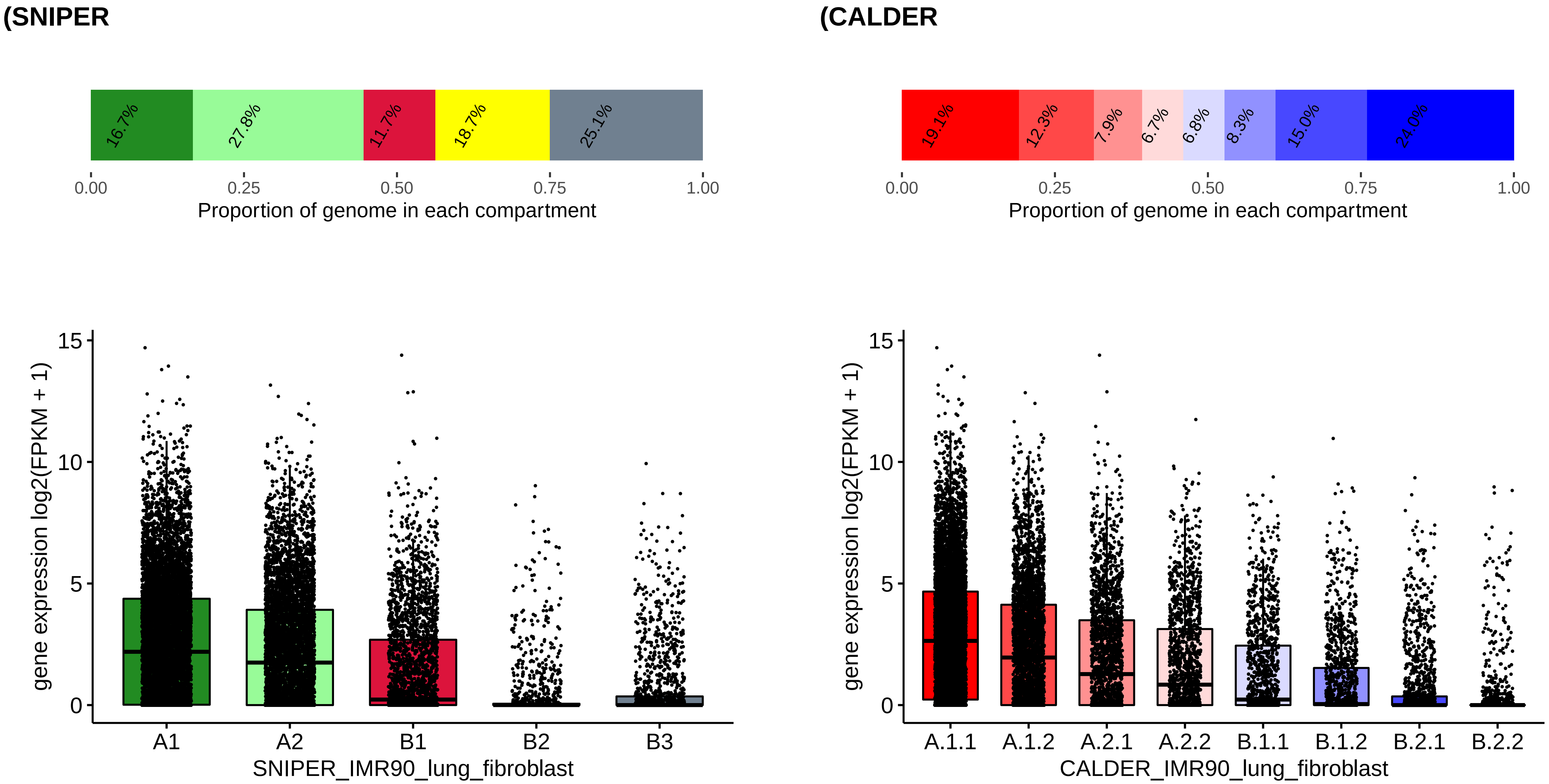
(SNIPER
16.7%
27.8%
11.7%
18.7%
25.1%
0.00
0.25
0.50
0.75
1.00
Proportion of genome in each compartment
(CALDER
19.1%
12.3%
7.9%
6.7%
6.8%
8.3%
15.0%
24.0%
0.00
0.25
0.50
0.75
1.00
Proportion of genome in each compartment
gene expression log2(FPKM + 1)
15
10
5
0
A1
A2
B1
B2
B3
SNIPER_IMR90_lung_fibroblast
gene expression log2(FPKM + 1)
15
10
5
0
A.1.1
A.1.2
A.2.1
A.2.2
B.1.1
B.1.2
B.2.1
B.2.2
CALDER_IMR90_lung_fibroblast

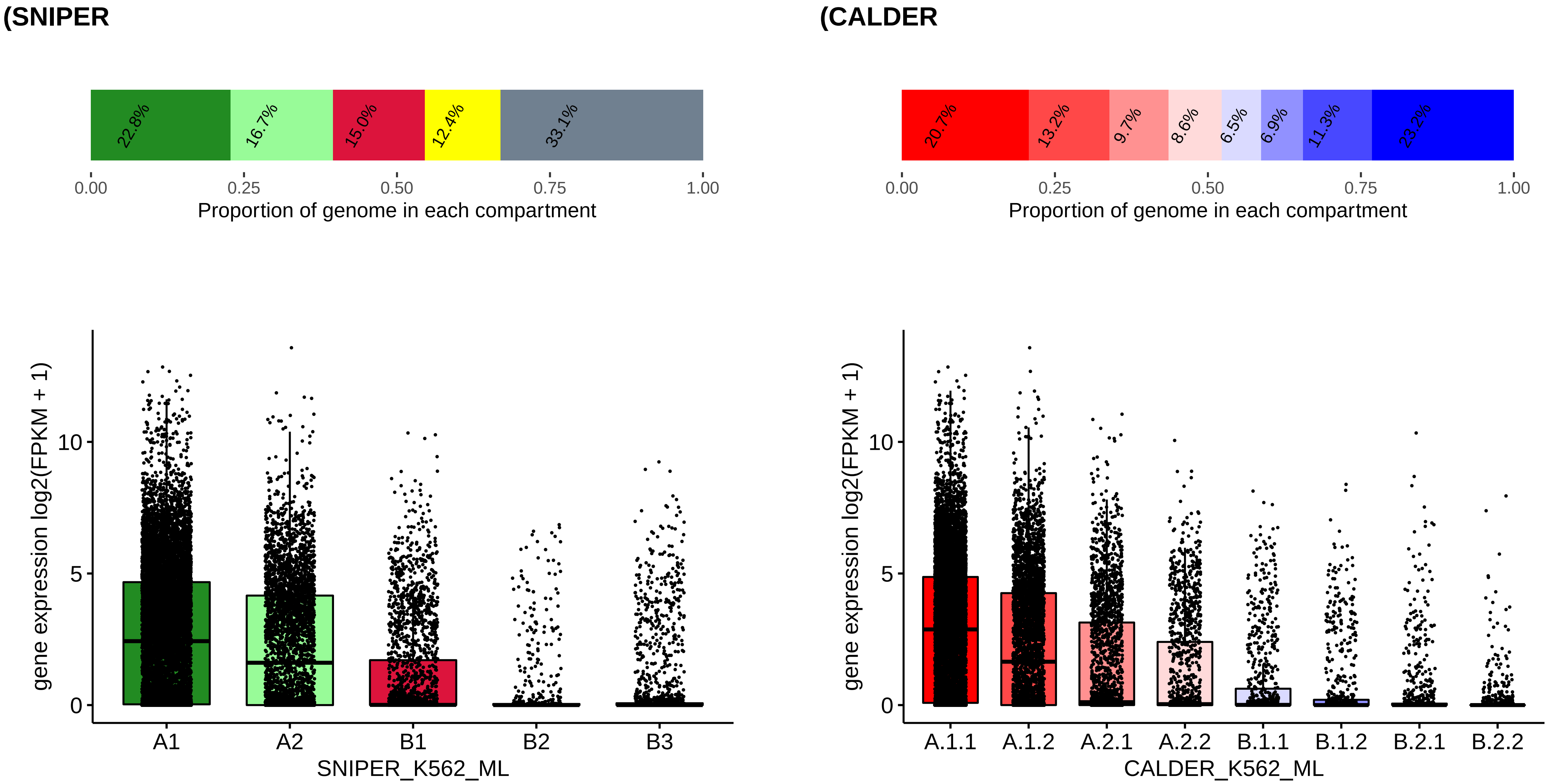
(SNIPER
22.8%
16.7%
15.0%
12.4%
33.1%
0.00
0.25
0.50
0.75
1.00
Proportion of genome in each compartment
gene expression log2(FPKM + 1)
0
5
10
A1
A2
B1
B2
B3
SNIPER_K562_ML
(CALDER
20.7%
13.2%
9.7%
8.6%
6.5%
6.9%
11.3%
23.2%
0.00
0.25
0.50
0.75
1.00
Proportion of genome in each compartment
gene expression log2(FPKM + 1)
0
5
10
A.1.1
A.1.2
A.2.1
A.2.2
B.1.1
B.1.2
B.2.1
B.2.2
CALDER_K562_ML

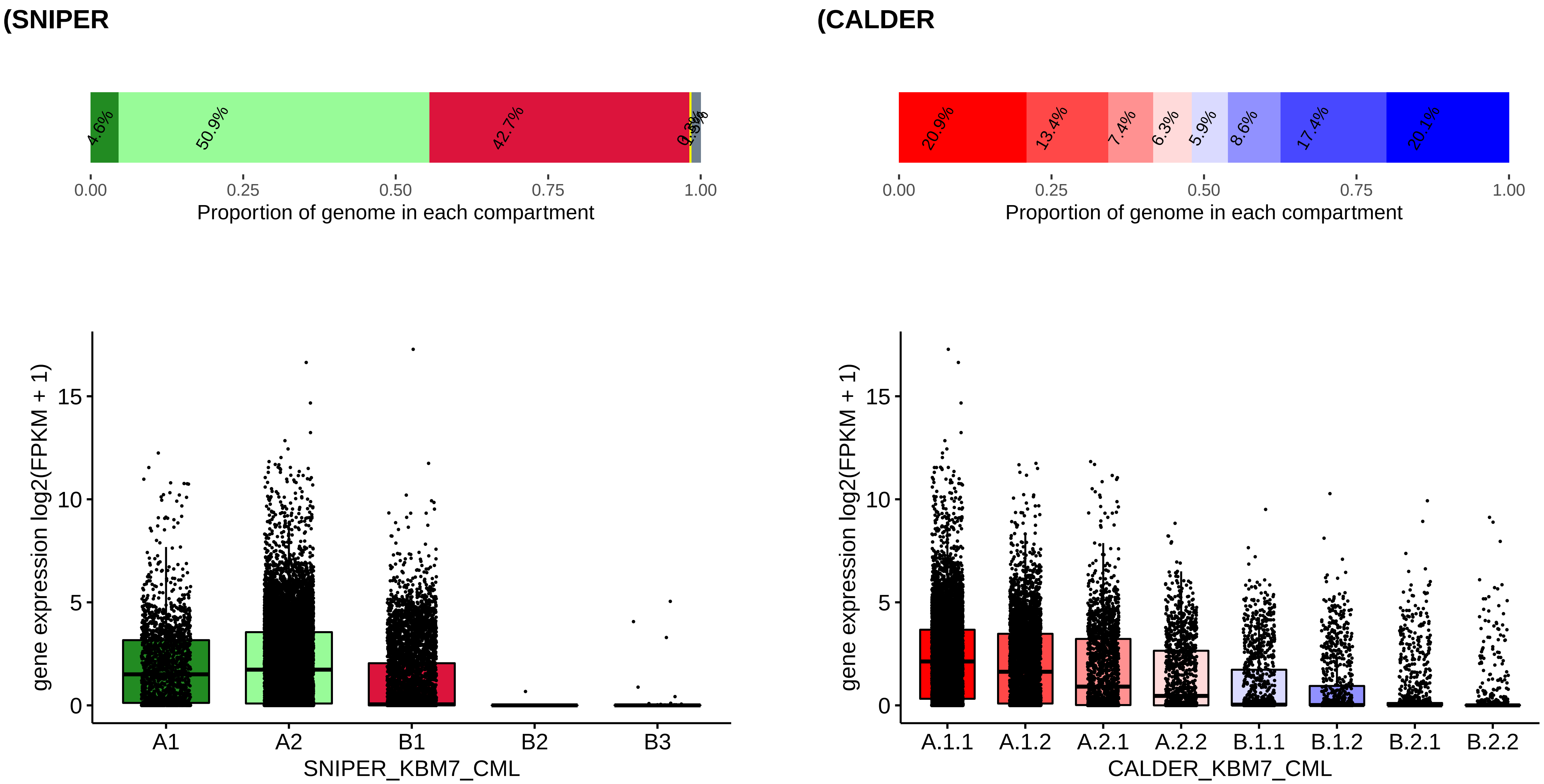
(SNIPER
4.6%
50.9%
42.7%
0.00
0.25
0.50
0.75
1.00
Proportion of genome in each compartment
gene expression log2(FPKM + 1)
0
5
10
15
A1
A2
B1
B2
B3
SNIPER_KBM7_CML
(CALDER
20.9%
13.4%
7.4%
6.3%
5.9%
8.6%
17.4%
20.1%
0.00
0.25
0.50
0.75
1.00
Proportion of genome in each compartment
gene expression log2(FPKM + 1)
0
5
10
15
A.1.1
A.1.2
A.2.1
A.2.2
B.1.1
B.1.2
B.2.1
B.2.2
CALDER_KBM7_CML

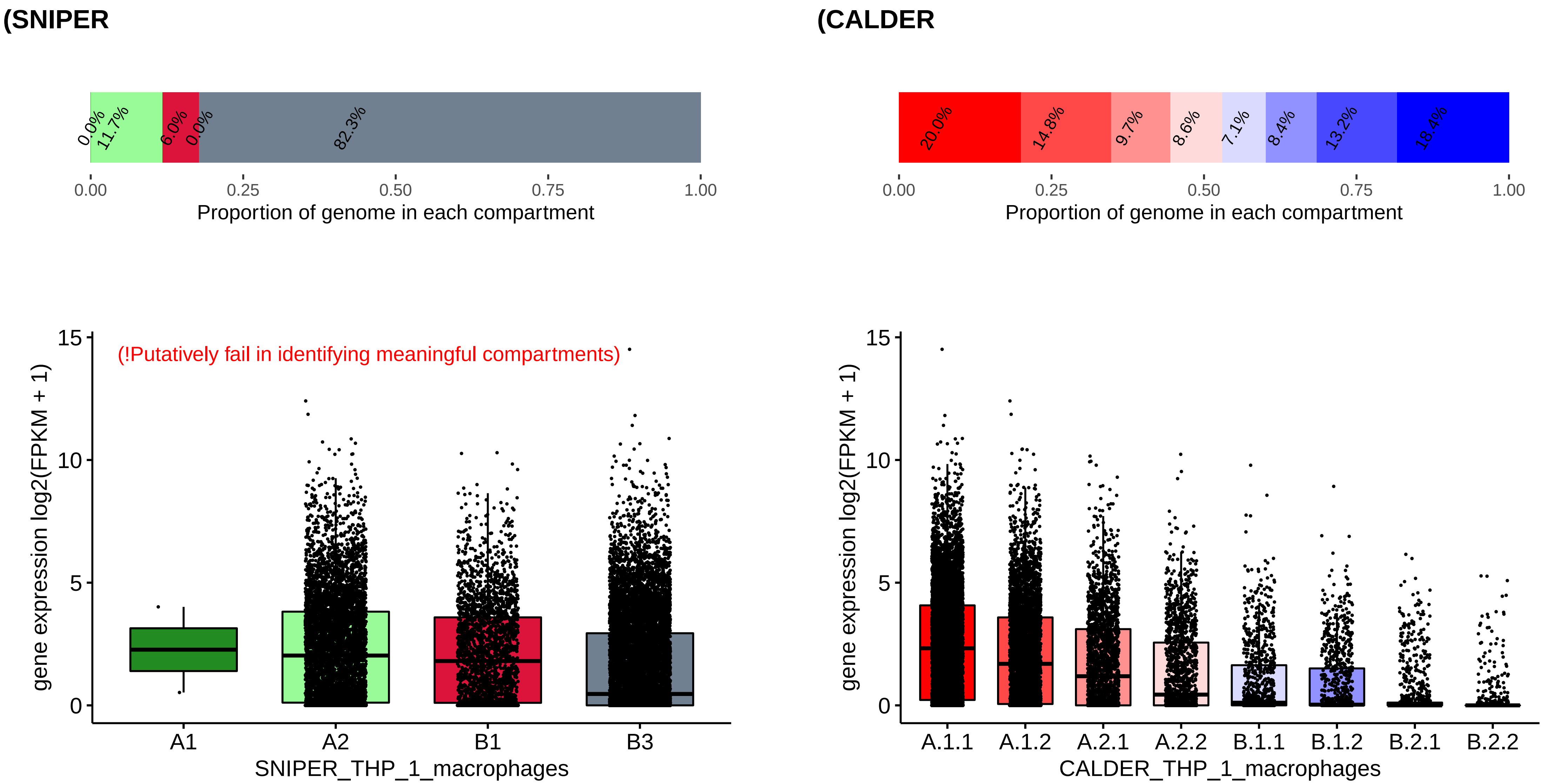

(SNIPER
0.0%
11.7%
6.0%
0.0%
82.3%
0.00
0.25
0.50
0.75
1.00
Proportion of genome in each compartment
(!Putatively fail in identifying meaningful compartments)
gene expression log2(FPKM + 1)
0
5
10
15
A1
A2
B1
B3
SNIPER_THP_1_macrophages
(CALDER
20.0%
14.8%
9.7%
8.6%
7.1%
8.4%
13.2%
18.4%
0.00
0.25
0.50
0.75
1.00
Proportion of genome in each compartment
gene expression log2(FPKM + 1)
0
5
10
15
A.1.1
A.1.2
A.2.1
A.2.2
B.1.1
B.1.2
B.2.1
B.2.2
CALDER_THP_1_macrophages

(SNIPER

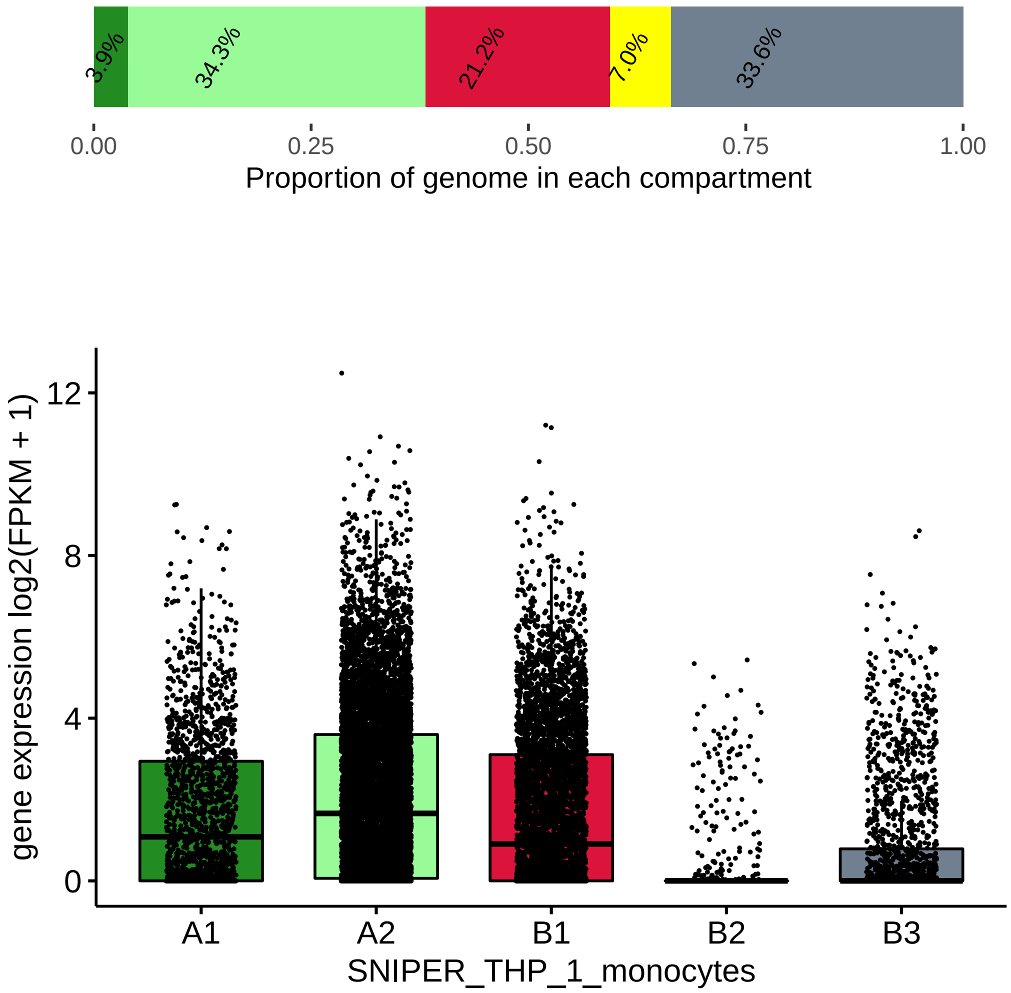


(CALDER

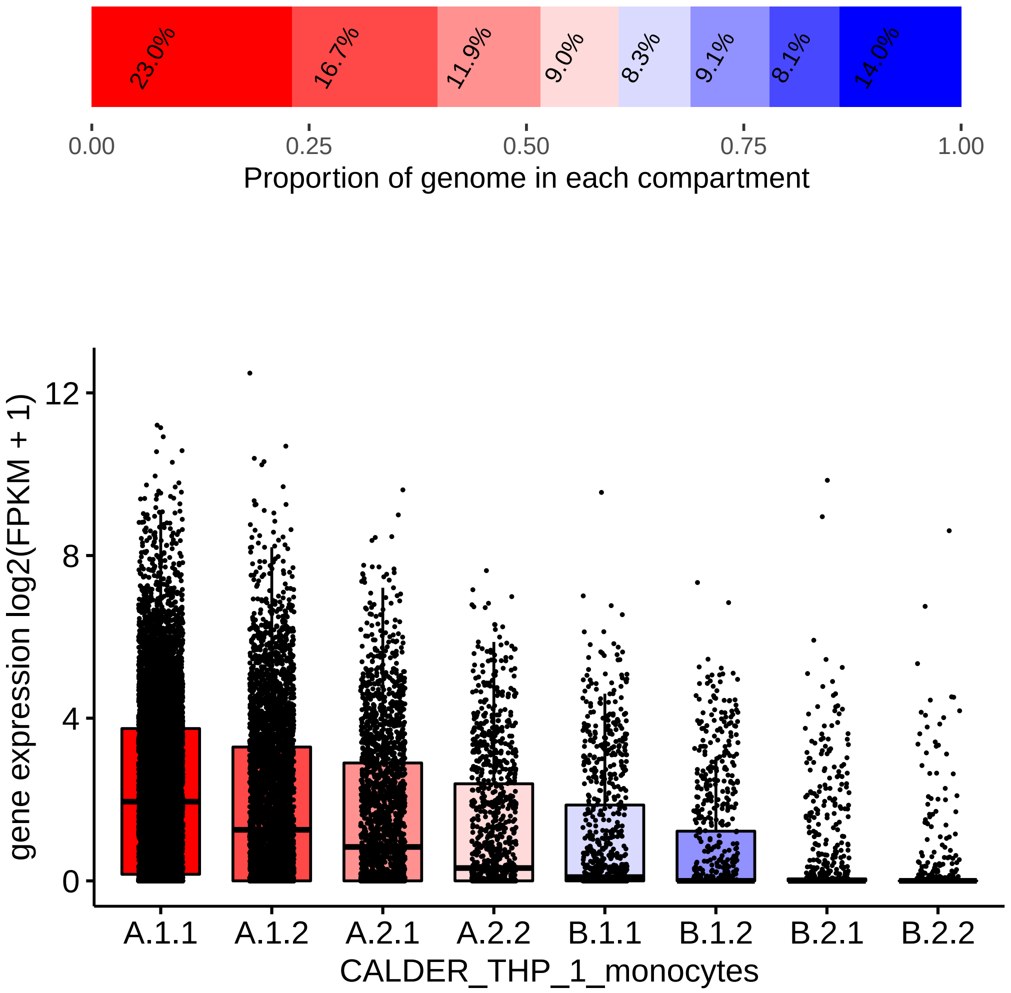

(SNIPER (CALDER

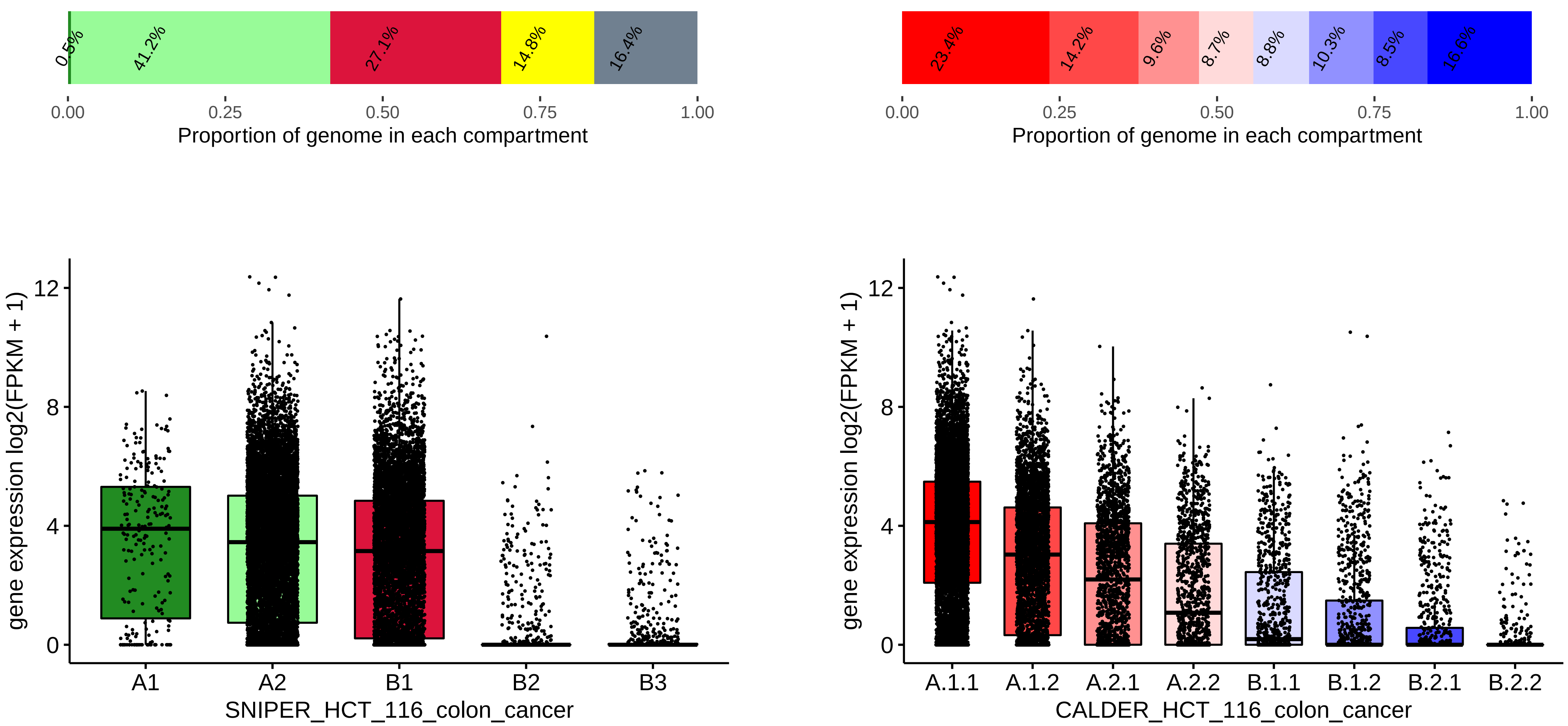

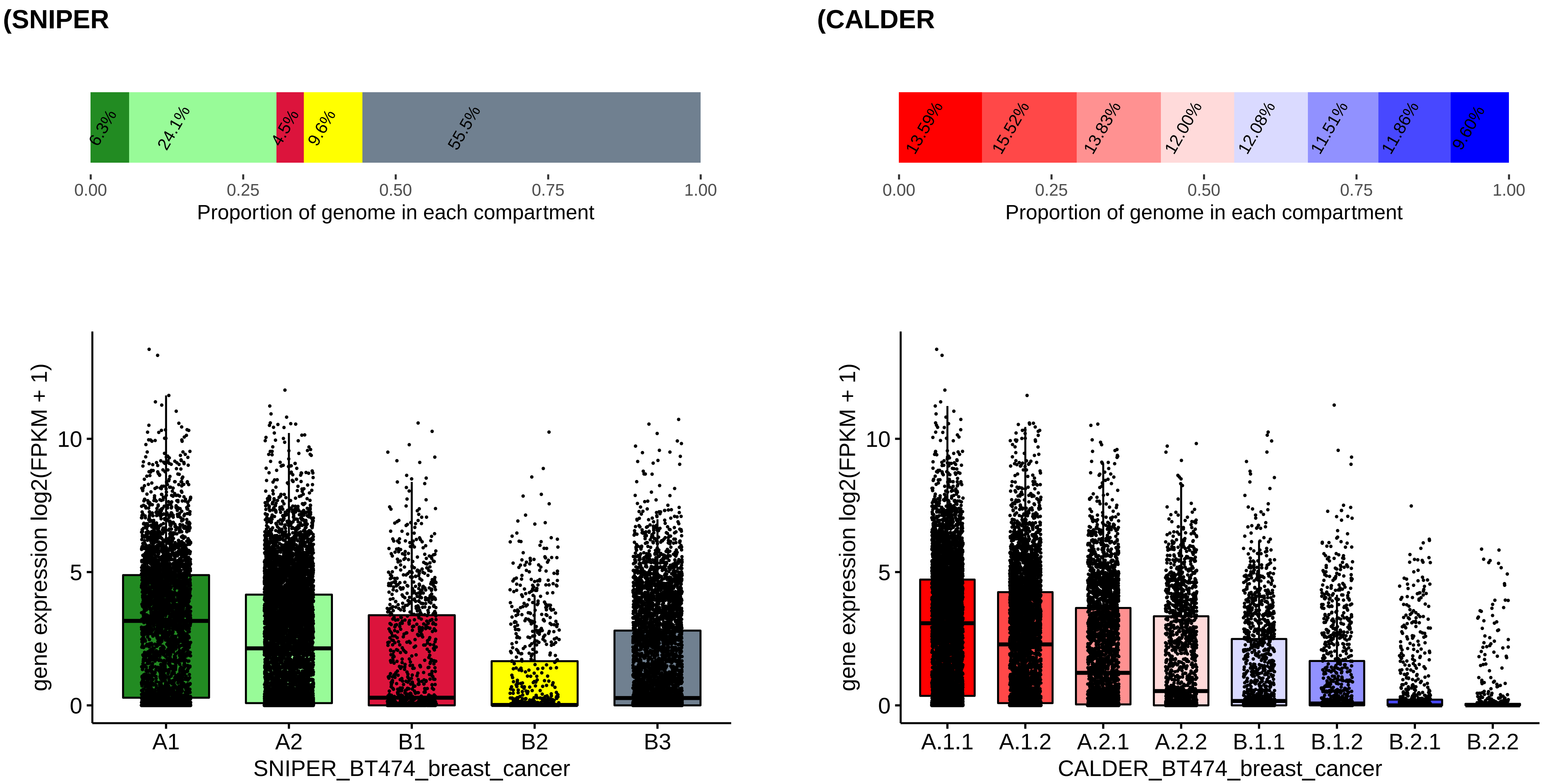

(SNIPER
(CALDER
6.3%
24.1%
4.5%
9.6%
55.5%
13.59%
15.52%
13.83%
12.00%
12.08%
11.51%
11.86%
9.60%
0.00
0.25
0.50
0.75
1.00
Proportion of genome in each compartment
Proportion of genome in each compartment
gene expression log2(FPKM + 1)
gene expression log2(FPKM + 1)
0
5
10
A1
A2
B1
B2
B3
SNIPER_BT474_breast_cancer
A.1.1
A.1.2
A.2.1
A.2.2
B.1.1
B.1.2
B.2.1
B.2.2
CALDER_BT474_breast_cancer

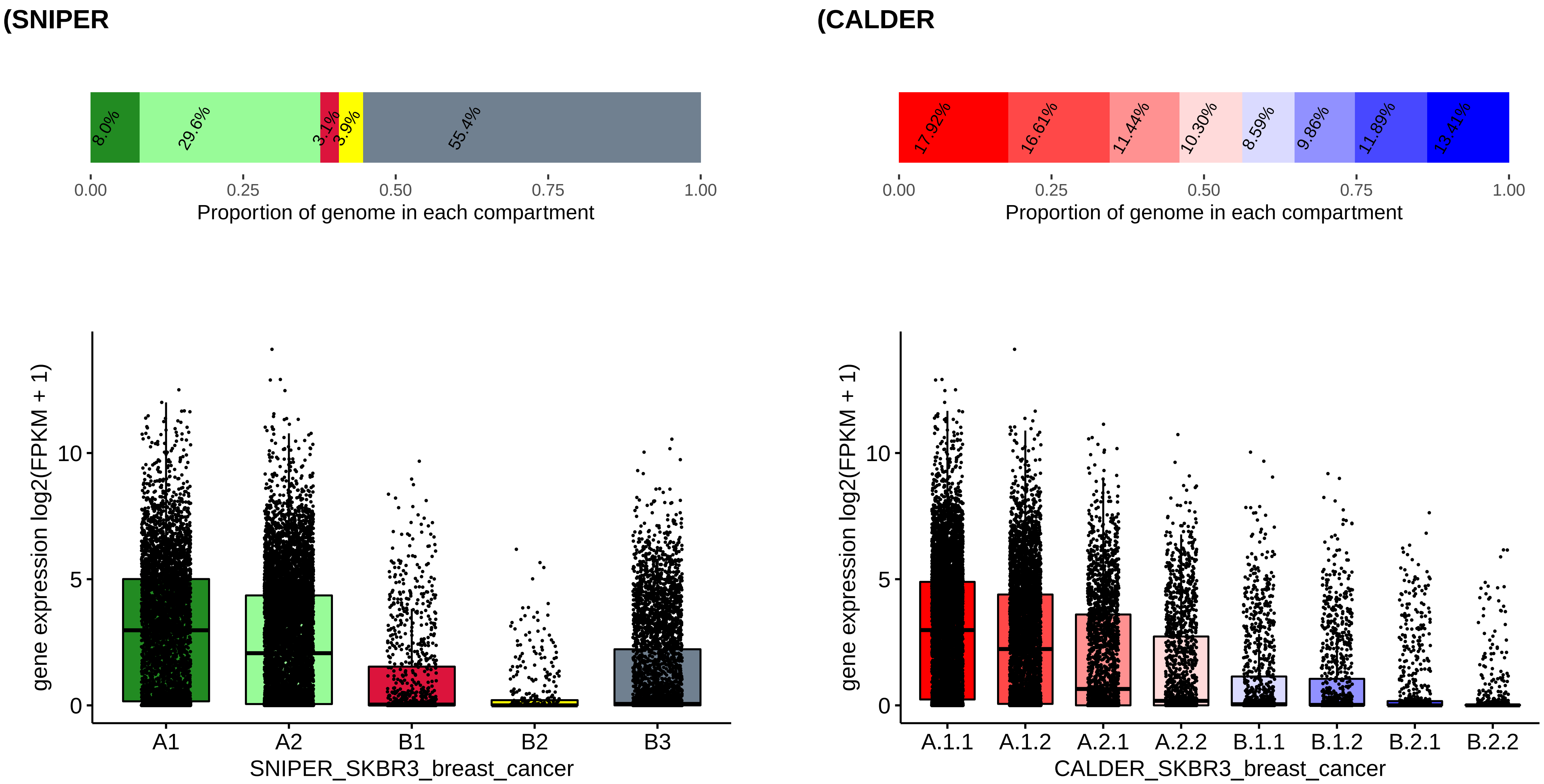

(SNIPER
8.0%
29.6%
3.1%
3.9%
55.4%
0.00
0.25
0.50
0.75
1.00
Proportion of genome in each compartment
gene expression log2(FPKM + 1)
0
5
10
A1
A2
B1
B2
B3
SNIPER_SKBR3_breast_cancer
(CALDER
17.92%
16.61%
11.44%
10.30%
8.59%
9.86%
11.89%
13.41%
0.00
0.25
0.50
0.75
1.00
Proportion of genome in each compartment
gene expression log2(FPKM + 1)
0
5
10
A.1.1
A.1.2
A.2.1
A.2.2
B.1.1
B.1.2
B.2.1
B.2.2
CALDER_SKBR3_breast_cancer

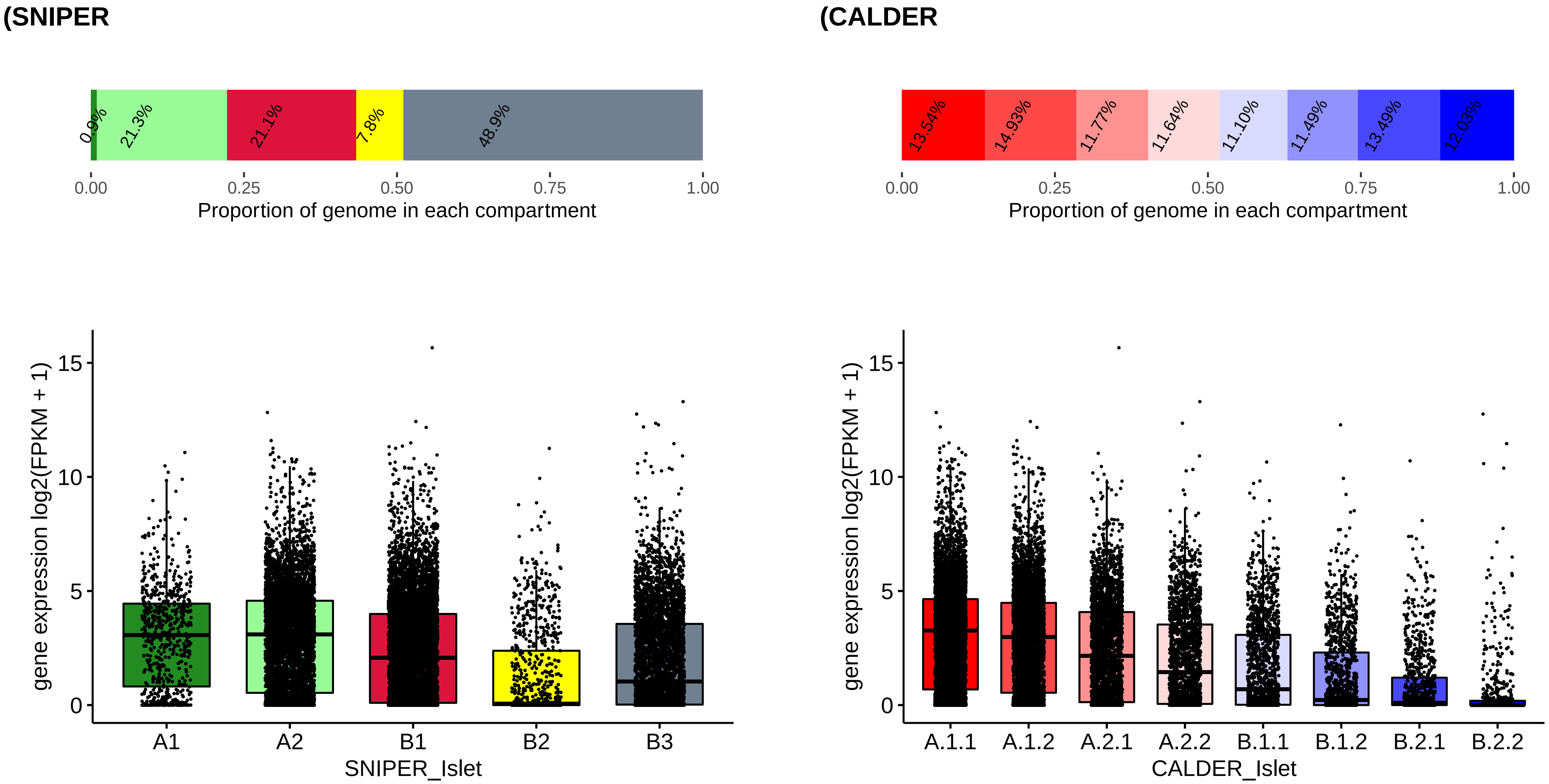
(SNIPER
0.9%
21.3%
21.1%
7.8%
48.9%
0.00
0.25
0.50
0.75
1.00
Proportion of genome in each compartment
gene expression log2(FPKM + 1)
0
5
10
15
A1
A2
B1
B2
B3
SNIPER_Islet
(CALDER
13.54%
14.93%
11.77%
11.64%
11.10%
11.49%
13.49%
12.03%
0.00
0.25
0.50
0.75
1.00
Proportion of genome in each compartment
gene expression log2(FPKM + 1)
0
5
10
15
A.1.1
A.1.2
A.2.1
A.2.2
B.1.1
B.1.2
B.2.1
B.2.2
CALDER_Islet

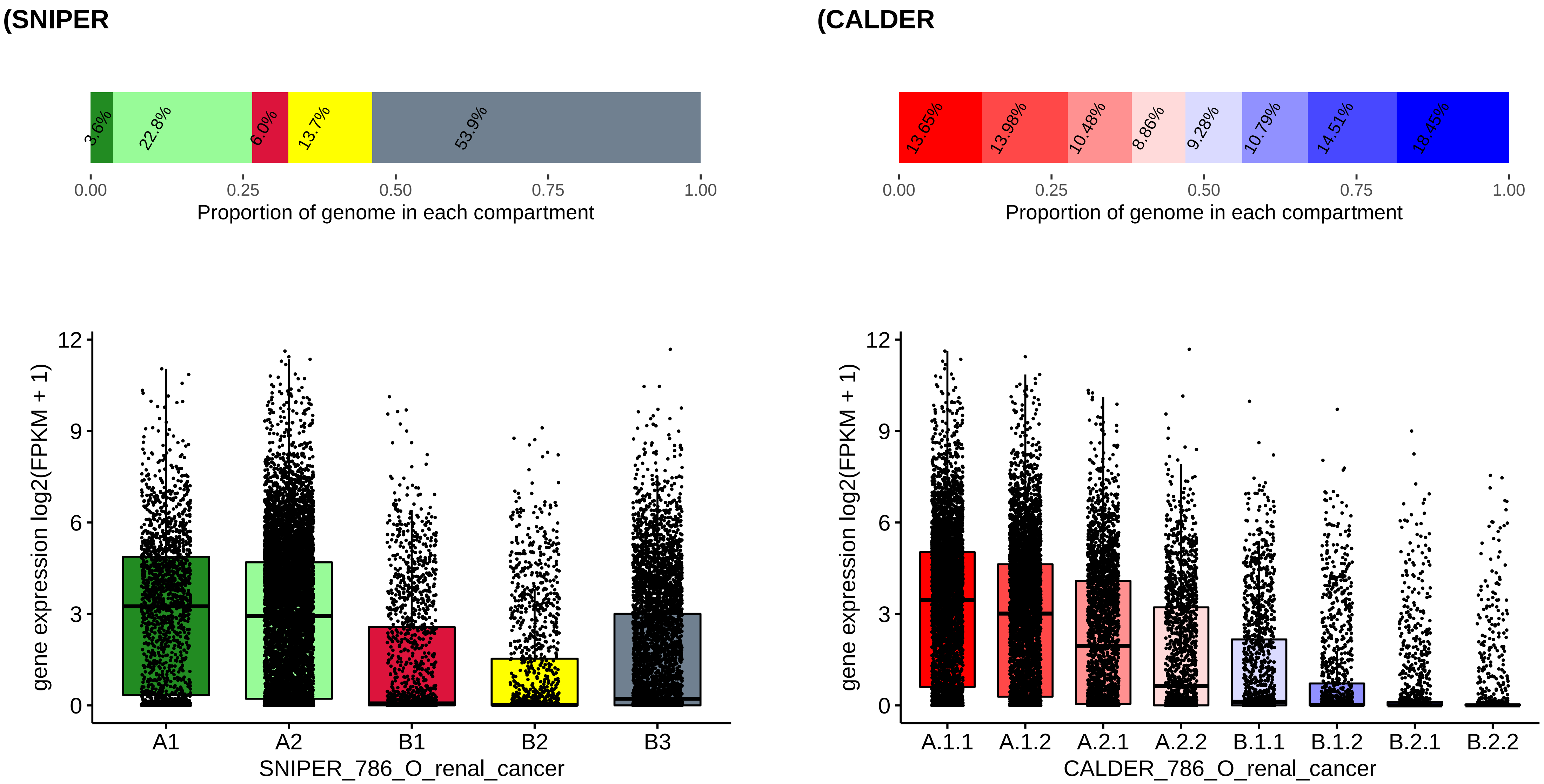

(SNIPER
3.6%
22.8%
6.0%
13.7%
53.9%
0.00
0.25
0.50
0.75
1.00
Proportion of genome in each compartment
gene expression log2(FPKM + 1)
12
9
6
3
0
A1
A2
B1
B2
B3
SNIPER_786_O_renal_cancer
(CALDER
13.65%
13.98%
10.48%
8.86%
9.28%
10.79%
14.51%
18.45%
0.00
0.25
0.50
0.75
1.00
Proportion of genome in each compartment
gene expression log2(FPKM + 1)
12
9
6
3
0
A.1.1
A.1.2
A.2.1
A.2.2
B.1.1
B.1.2
B.2.1
B.2.2
CALDER_786_O_renal_cancer

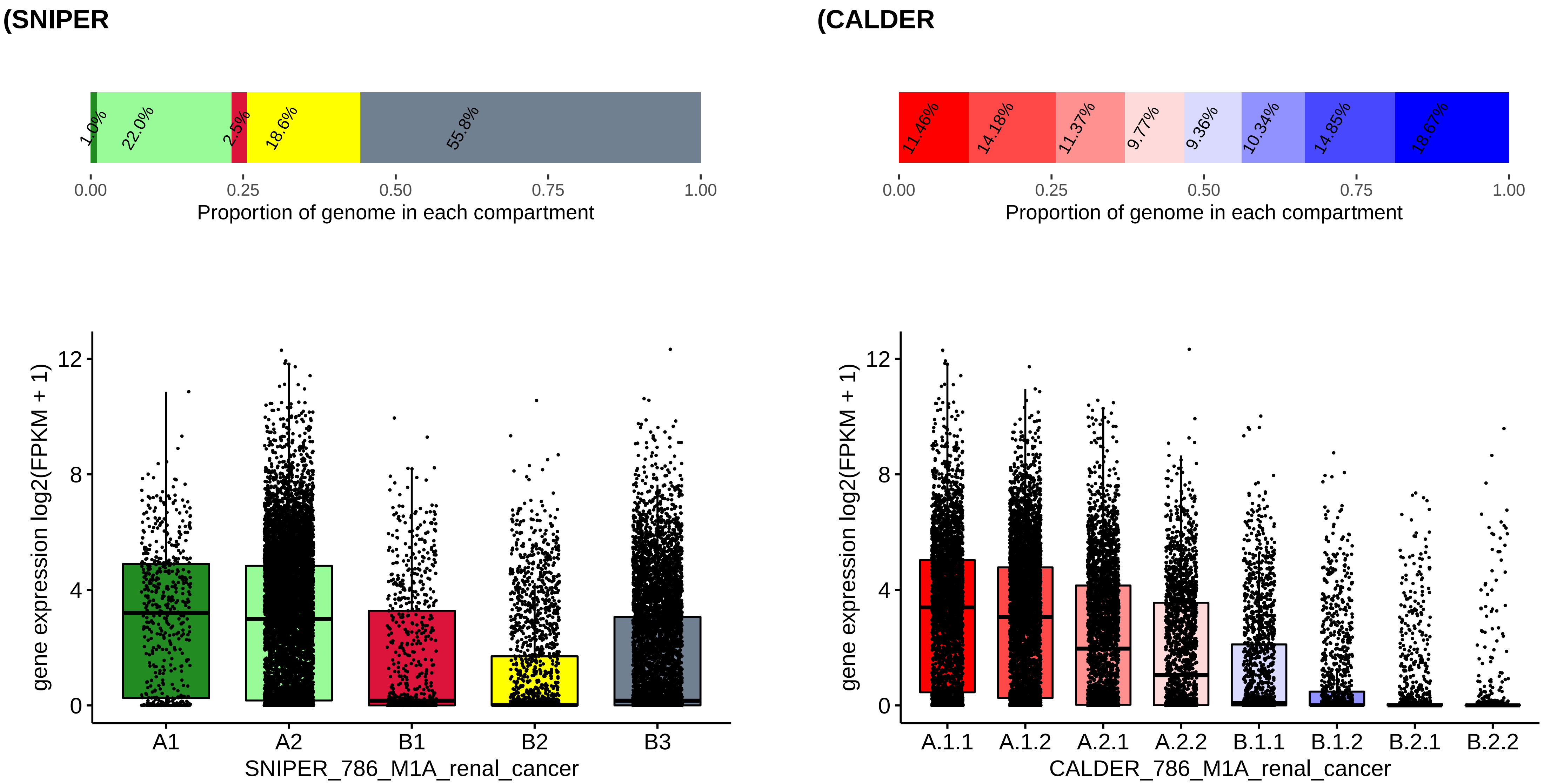
(SNIPER
1.0%
22.0%
2.5%
18.6%
55.8%
0.00
0.25
0.50
0.75
1.00
Proportion of genome in each compartment
gene expression log2(FPKM + 1)
0
4
8
12
A1
A2
B1
B2
B3
SNIPER_786_M1A_renal_cancer
(CALDER
11.46%
14.18%
11.37%
9.77%
9.36%
10.34%
14.85%
18.67%
0.00
0.25
0.50
0.75
1.00
Proportion of genome in each compartment
gene expression log2(FPKM + 1)
0
4
8
12
A.1.1
A.1.2
A.2.1
A.2.2
B.1.1
B.1.2
B.2.1
B.2.2
CALDER_786_M1A_renal_cancer

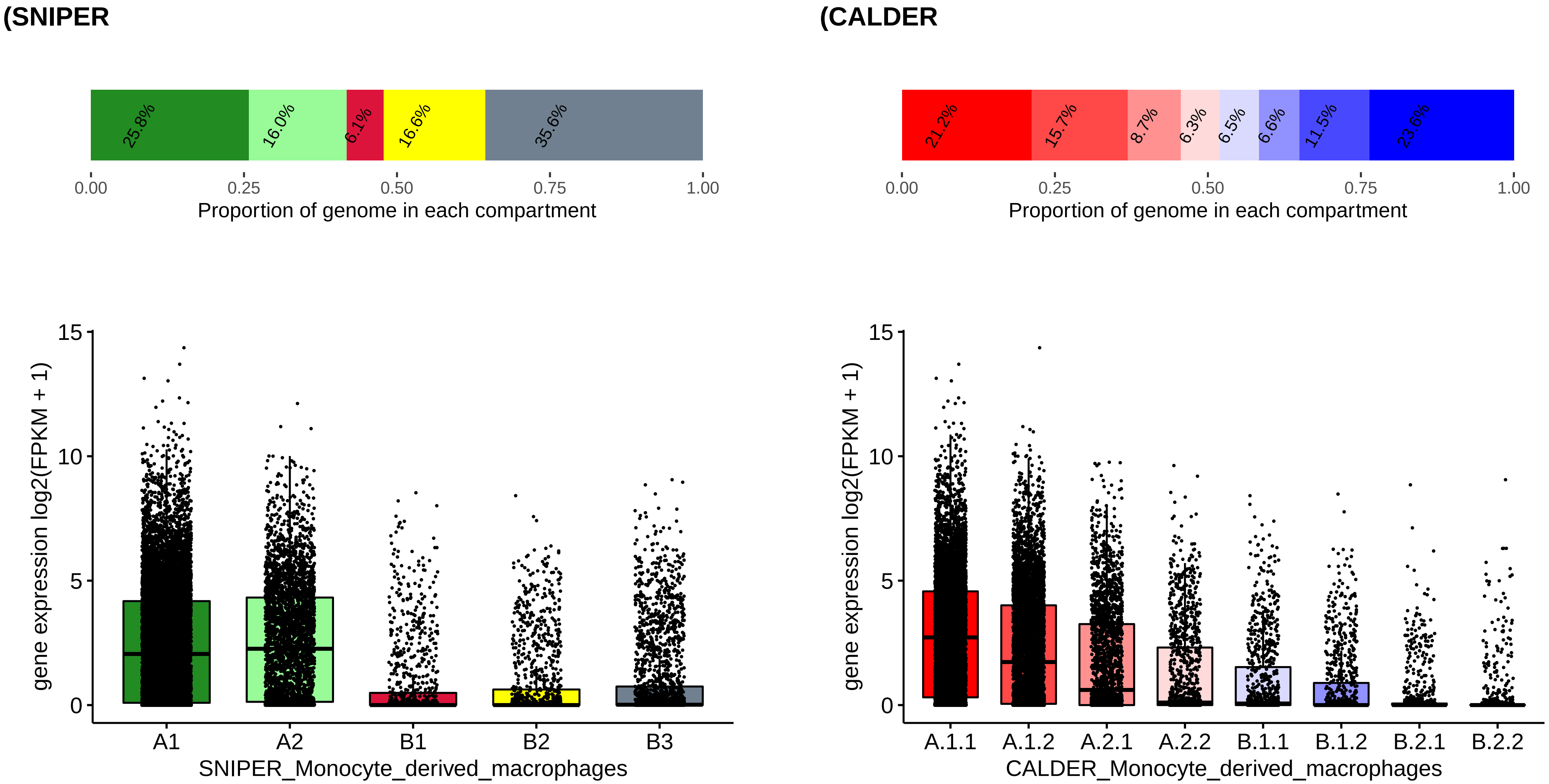

(SNIPER
25.8%
16.0%
6.1%
16.6%
35.6%
0.00
0.25
0.50
0.75
1.00
Proportion of genome in each compartment
(CALDER
21.2%
15.7%
8.7%
6.3%
6.5%
6.6%
11.5%
23.6%
0.00
0.25
0.50
0.75
1.00
Proportion of genome in each compartment
gene expression log2(FPKM + 1)
15
10
5
0
A1
A2
B1
B2
B3
SNIPER_Monocyte_derived_macrophages
gene expression log2(FPKM + 1)
15
10
5
0
A.1.1
A.1.2
A.2.1
A.2.2
B.1.1
B.1.2
B.2.1
B.2.2
CALDER_Monocyte_derived_macrophages

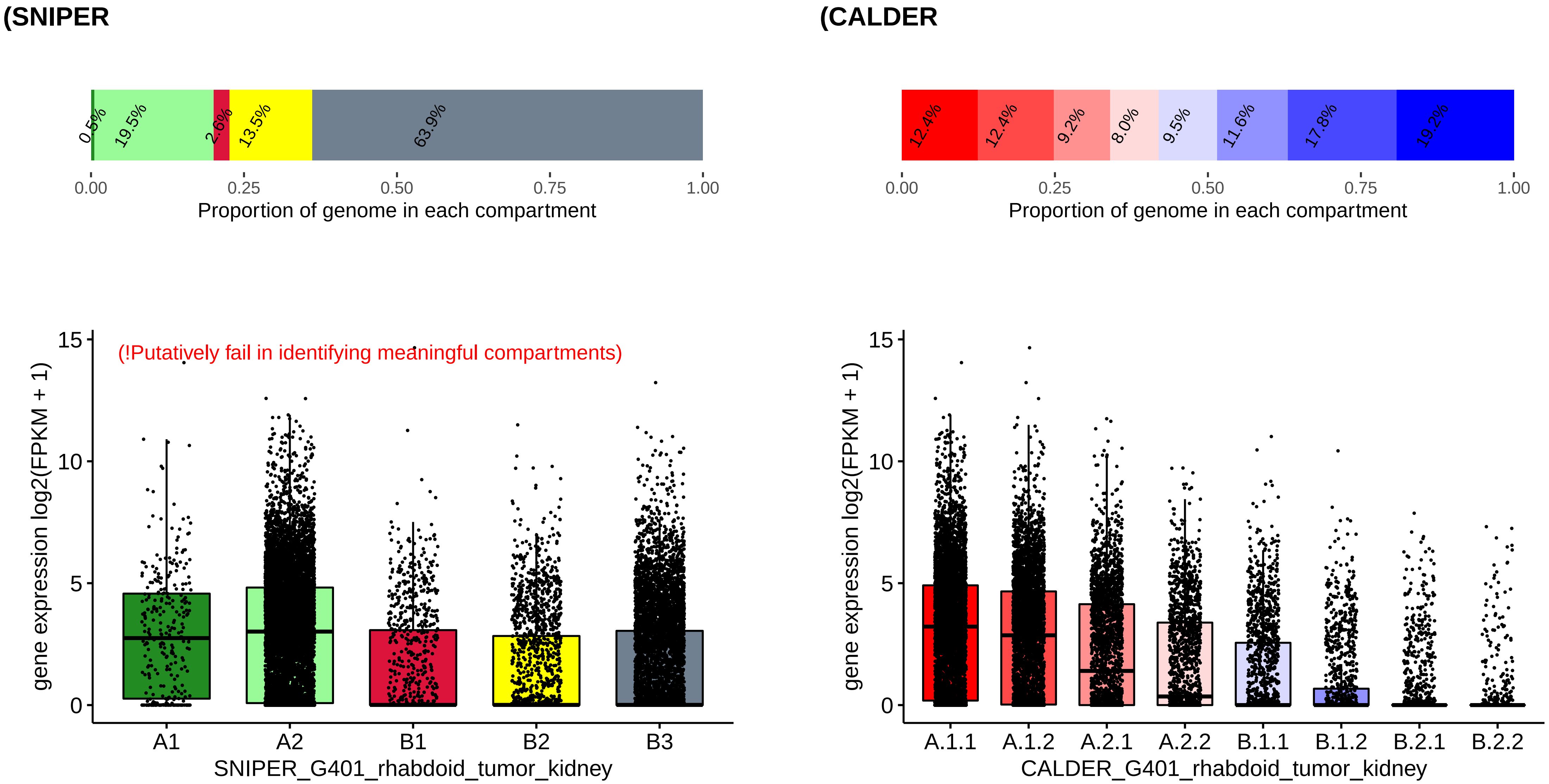

(SNIPER
0.5%
19.5%
2.6%
13.5%
63.9%
0.00
0.25
0.50
0.75
1.00
Proportion of genome in each compartment
(!Putatively fail in identifying meaningful compartments)
gene expression log2(FPKM + 1)
15
10
5
0
A1
A2
B1
B2
B3
SNIPER_G401_rhabdoid_tumor_kidney
(CALDER
12.4%
12.4%
9.2%
8.0%
9.5%
11.6%
17.8%
19.2%
0.00
0.25
0.50
0.75
1.00
Proportion of genome in each compartment
gene expression log2(FPKM + 1)
15
10
5
0
A.1.1
A.1.2
A.2.1
A.2.2
B.1.1
B.1.2
B.2.1
B.2.2
CALDER_G401_rhabdoid_tumor_kidney

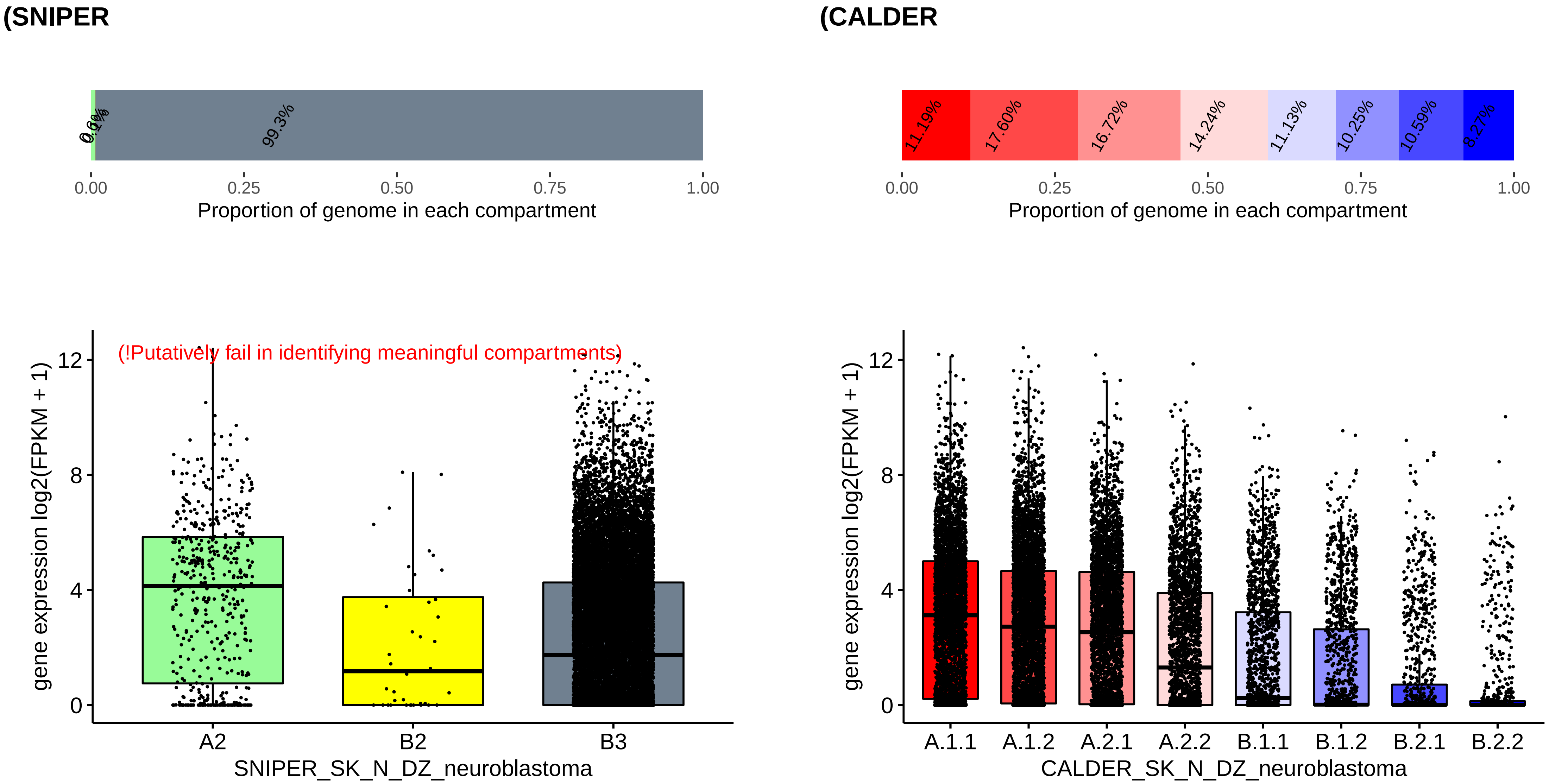
(SNIPER
(CALDER
0.6%
0.1%
99.3%
0.00
0.25
0.50
0.75
1.00
Proportion of genome in each compartment
11.19%
17.60%
16.72%
14.24%
11.13%
10.25%
10.59%
8.27%
Proportion of genome in each compartment
(!Putatively fail in identifying meaningful compartments)
gene expression log2(FPKM + 1)
0
4
8
12
A2
B2
B3
SNIPER_SK_N_DZ_neuroblastoma
A.1.1
A.1.2
A.2.1
A.2.2
B.1.1
B.1.2
B.2.1
B.2.2
CALDER_SK_N_DZ_neuroblastoma

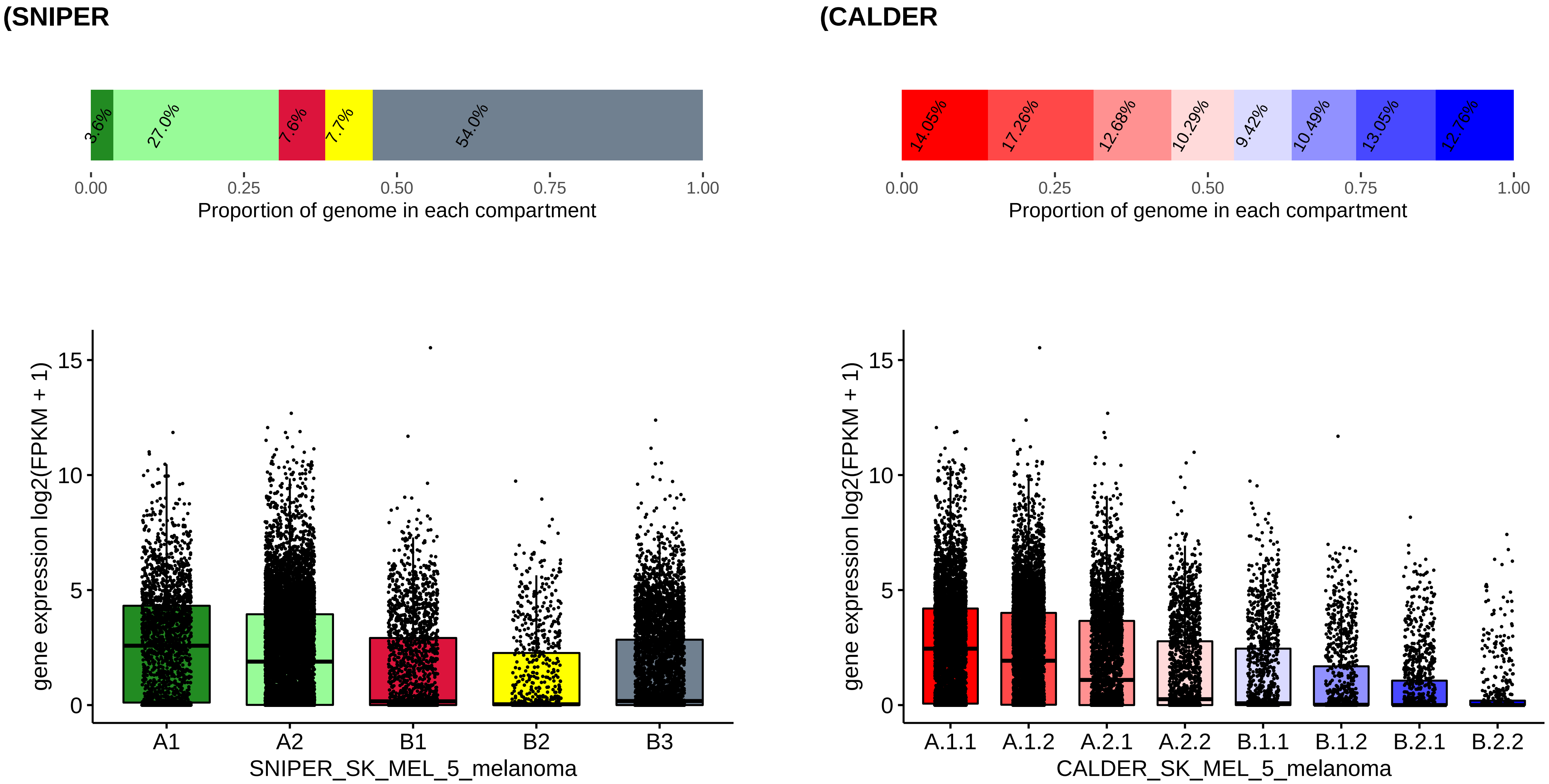

(SNIPER
3.6%
27.0%
7.6%
7.7%
54.0%
0.00
0.25
0.50
0.75
1.00
Proportion of genome in each compartment
(CALDER
14.05%
17.26%
12.68%
10.29%
9.42%
10.49%
13.05%
12.76%
0.00
0.25
0.50
0.75
1.00
Proportion of genome in each compartment
gene expression log2(FPKM + 1)
0
5
10
15
A1
A2
B1
B2
B3
SNIPER_SK_MEL_5_melanoma
gene expression log2(FPKM + 1)
0
5
10
15
A.1.1
A.1.2
A.2.1
A.2.2
B.1.1
B.1.2
B.2.1
B.2.2
CALDER_SK_MEL_5_melanoma

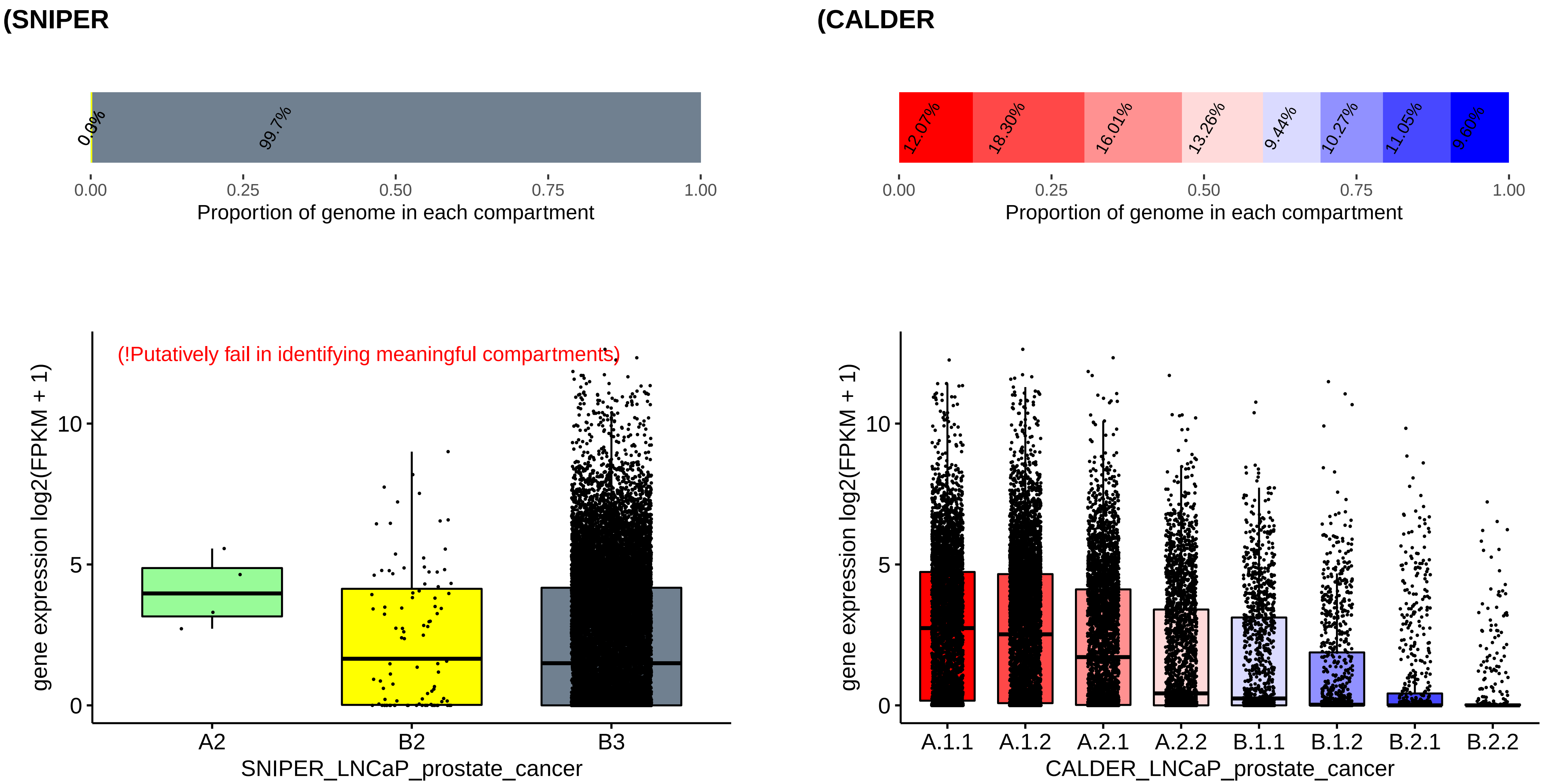
(SNIPER
0.3%
99.7%
0.00
0.25
0.50
0.75
1.00
Proportion of genome in each compartment
(!Putatively fail in identifying meaningful compartments)
gene expression log2(FPKM + 1)
0
5
10
A2
B2
B3
SNIPER_LNCaP_prostate_cancer
(CALDER
12.07%
18.30%
16.01%
13.26%
9.44%
10.27%
11.05%
9.60%
0.00
0.25
0.50
0.75
1.00
Proportion of genome in each compartment
gene expression log2(FPKM + 1)
0
5
10
A.1.1
A.1.2
A.2.1
A.2.2
B.1.1
B.1.2
B.2.1
B.2.2
CALDER_LNCaP_prostate_cancer

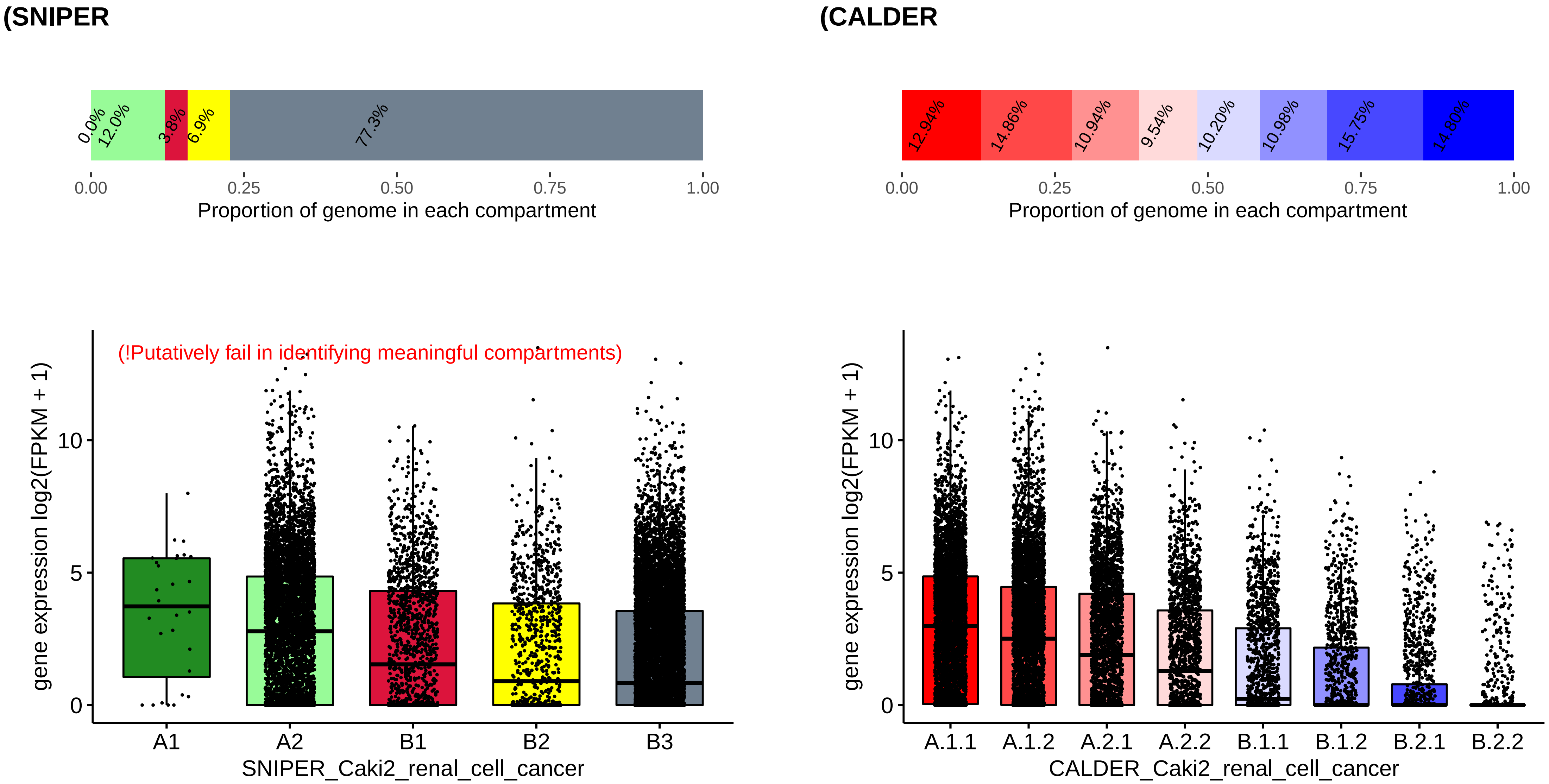

(SNIPER
(CALDER
0.0%
12.0%
3.8%
6.9%
77.3%
0.00
0.25
0.50
0.75
1.00
Proportion of genome in each compartment
12.94%
14.86%
10.94%
9.54%
10.20%
10.98%
15.75%
14.80%
Proportion of genome in each compartment
(!Putatively fail in identifying meaningful compartments)
gene expression log2(FPKM + 1)
0
5
10
A1
A2
B1
B2
B3
SNIPER_Caki2_renal_cell_cancer
gene expression log2(FPKM + 1)
A.1.1
A.1.2
A.2.1
A.2.2
B.1.1
B.1.2
B.2.1
B.2.2
CALDER_Caki2_renal_cell_cancer

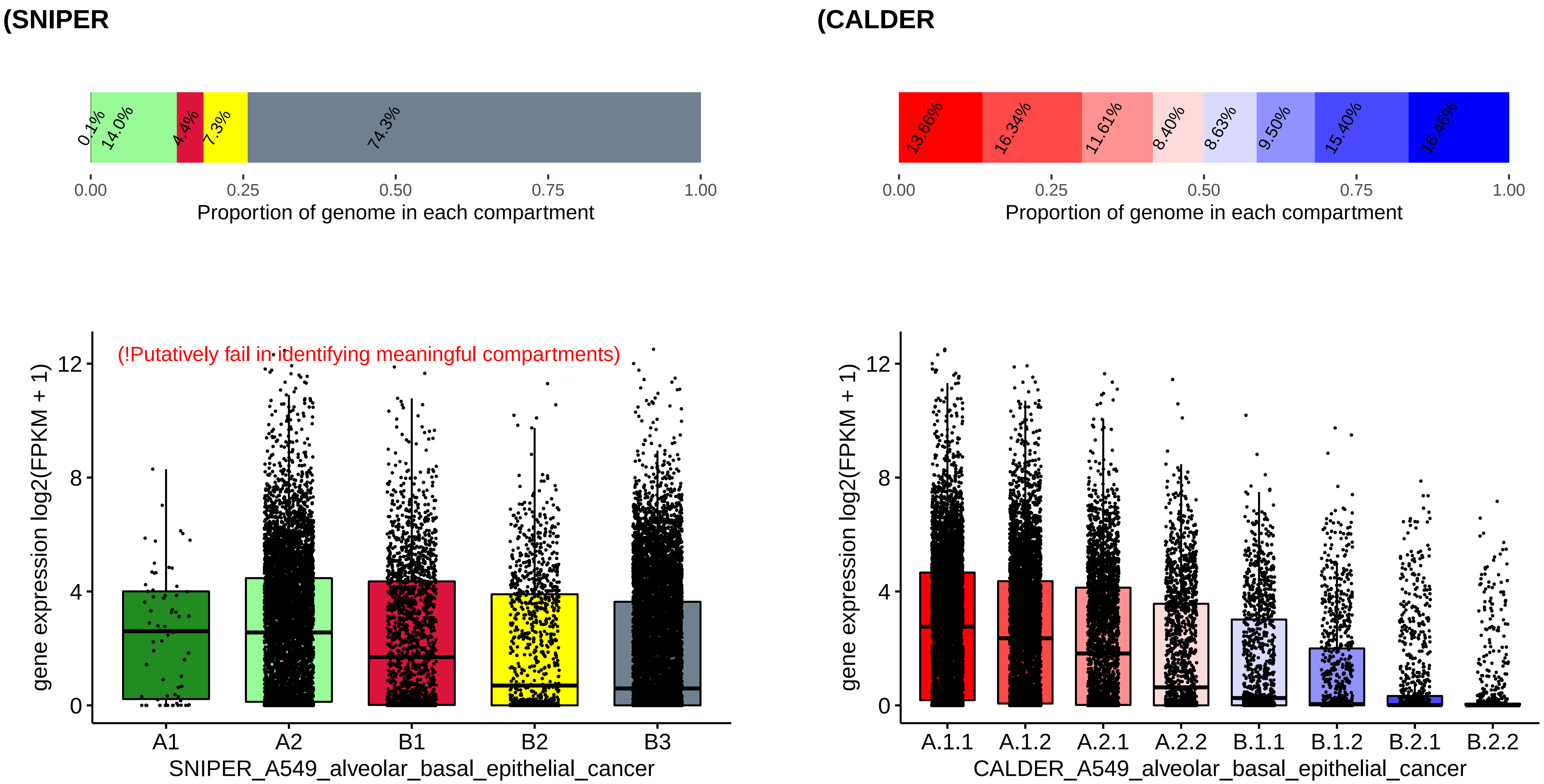

(SNIPER
0.1%
14.0%
4.4%
7.3%
74.3%
0.00
0.25
0.50
0.75
1.00
Proportion of genome in each compartment
(CALDER
13.66%
16.34%
11.61%
8.40%
8.63%
9.50%
15.40%
16.46%
0.00
0.25
0.50
0.75
1.00
Proportion of genome in each compartment
(!Putatively fail in identifying meaningful compartments)
gene expression log2(FPKM + 1)
12
8
4
0
A1
A2
B1
B2
B3
SNIPER_A549_alveolar_basal_epithelial_cancer
gene expression log2(FPKM + 1)
12
8
4
0
A.1.1
A.1.2
A.2.1
A.2.2
B.1.1
B.1.2
B.2.1
B.2.2
CALDER_A549_alveolar_basal_epithelial_cancer

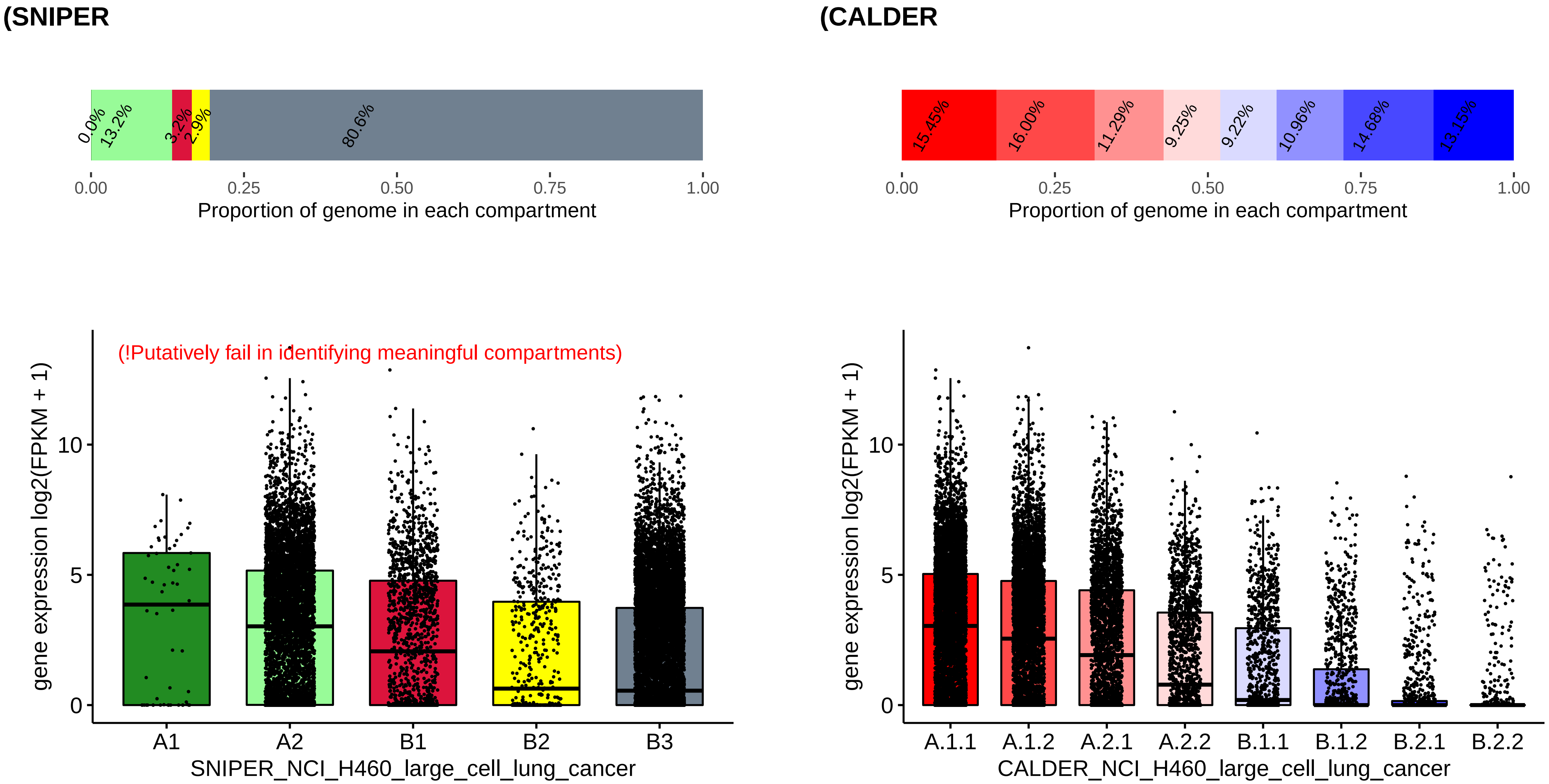
(SNIPER
(CALDER
0.0%
13.2%
3.2%
2.9%
80.6%
15.45%
16.00%
11.29%
9.25%
9.22%
10.96%
14.68%
13.15%
0.00
0.25
0.50
0.75
1.00
Proportion of genome in each compartment
(!Putatively fail in identifying meaningful compartments)
gene expression log2(FPKM + 1)
0
5
10
A1
A2
B1
B2
B3
SNIPER_NCI_H460_large_cell_lung_cancer
A.1.1
A.1.2
A.2.1
A.2.2
B.1.1
B.1.2
B.2.1
B.2.2
CALDER_NCI_H460_large_cell_lung_cancer

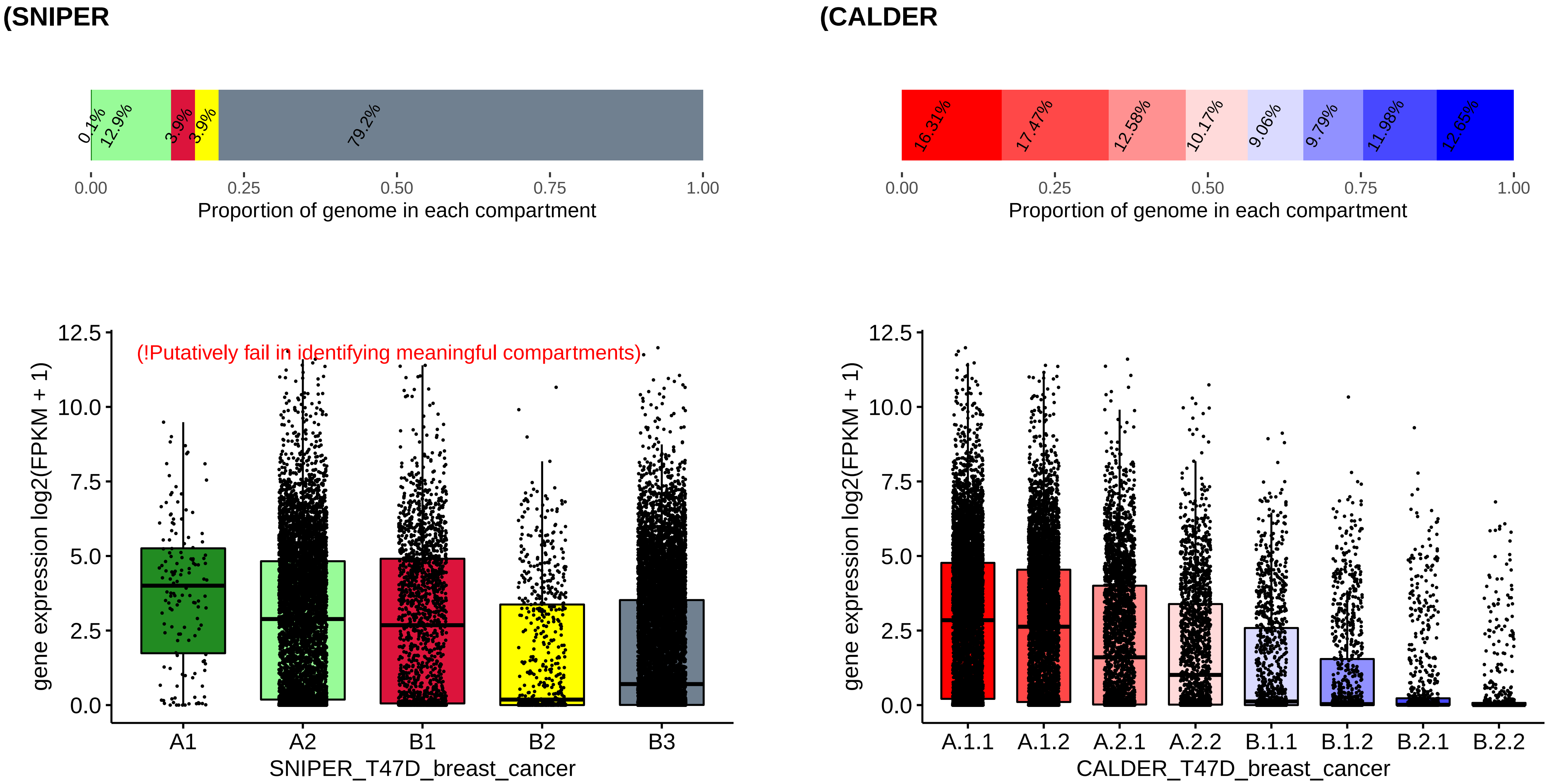

(SNIPER
0.1%
12.9%
3.9%
3.9%
79.2%
0.00
0.25
0.50
0.75
1.00
Proportion of genome in each compartment
(CALDER
16.31%
17.47%
12.58%
10.17%
9.06%
9.79%
11.98%
12.65%
0.00
0.25
0.50
0.75
1.00
Proportion of genome in each compartment
(!Putatively fail in identifying meaningful compartments)
gene expression log2(FPKM + 1)
12.5
10.0
7.5
5.0
2.5
0.0
A1
A2
B1
B2
B3
SNIPER_T47D_breast_cancer
gene expression log2(FPKM + 1)
12.5
10.0
7.5
5.0
2.5
0.0
A.1.1
A.1.2
A.2.1
A.2.2
B.1.1
B.1.2
B.2.1
B.2.2
CALDER_T47D_breast_cancer

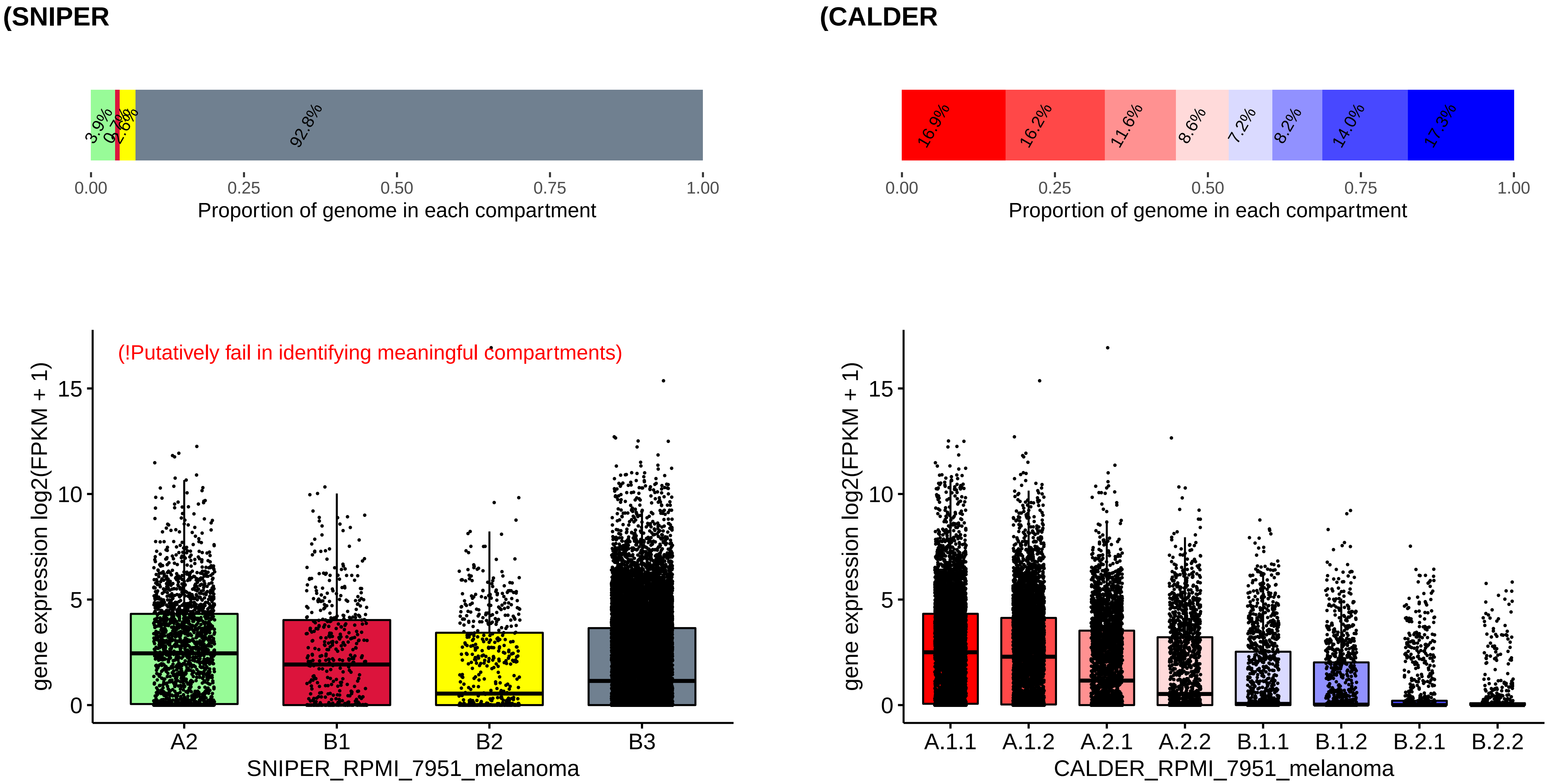
(SNIPER
3.9%
0.7%
2.6%
92.8%
0.00
0.25
0.50
0.75
1.00
Proportion of genome in each compartment
(!Putatively fail in identifying meaningful compartments)
gene expression log2(FPKM + 1)
0
5
10
15
A2
B1
B2
B3
SNIPER_RPMI_7951_melanoma
(CALDER
16.9%
16.2%
11.6%
8.6%
7.2%
8.2%
14.0%
17.3%
0.00
0.25
0.50
0.75
1.00
Proportion of genome in each compartment
gene expression log2(FPKM + 1)
0
5
10
15
A.1.1
A.1.2
A.2.1
A.2.2
B.1.1
B.1.2
B.2.1
B.2.2
CALDER_RPMI_7951_melanoma

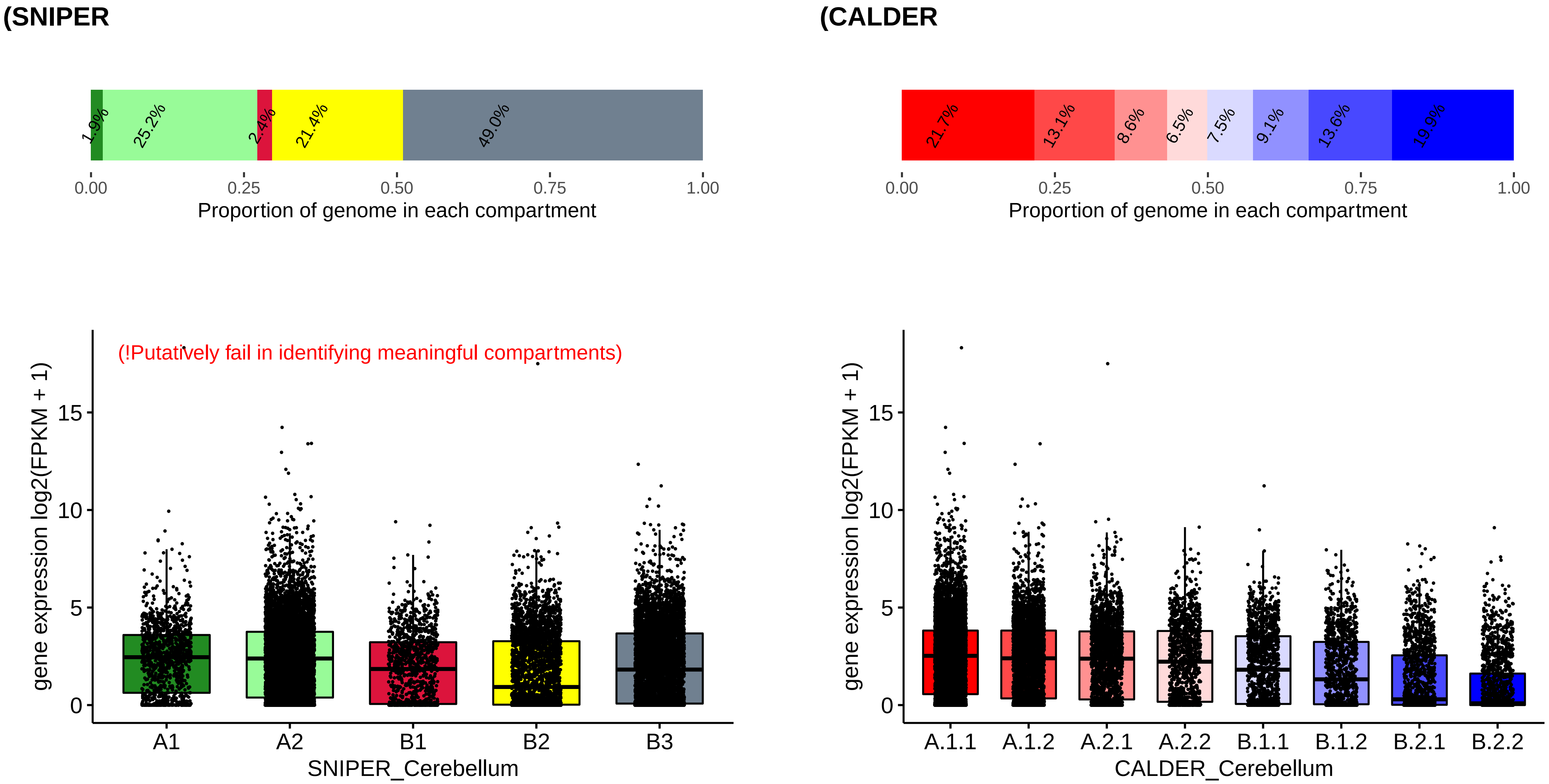
(SNIPER
(CALDER
1.9%
25.2%
2.4%
21.4%
49.0%
0.00
0.25
0.50
0.75
1.00
Proportion of genome in each compartment
21.7%
13.1%
8.6%
6.5%
7.5%
9.1%
13.6%
19.9%
0.00
0.25
0.50
0.75
1.00
Proportion of genome in each compartment
(!Putatively fail in identifying meaningful compartments)
gene expression log2(FPKM + 1)
0
5
10
15
A1
A2
B1
B2
B3
SNIPER_Cerebellum
gene expression log2(FPKM + 1)
0
5
10
15
A.1.1
A.1.2
A.2.1
A.2.2
B.1.1
B.1.2
B.2.1
B.2.2
CALDER_Cerebellum

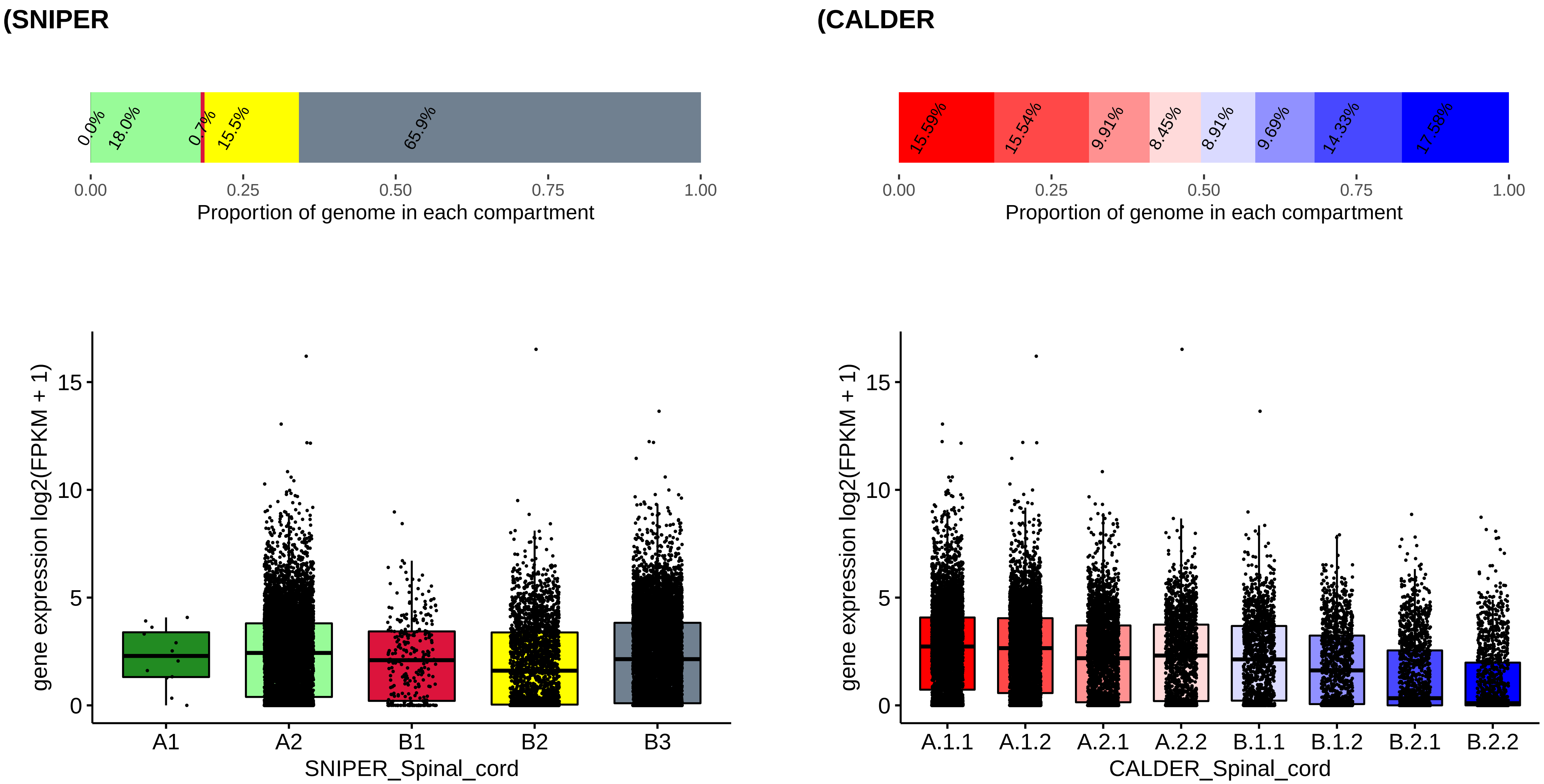

(SNIPER
0.0%
18.0%
0.7%
15.5%
65.9%
0.00
0.25
0.50
0.75
1.00
Proportion of genome in each compartment
gene expression log2(FPKM + 1)
0
5
10
15
A1
A2
B1
B2
B3
SNIPER_Spinal_cord
(CALDER
15.59%
15.54%
9.91%
8.45%
8.91%
9.69%
14.33%
17.58%
0.00
0.25
0.50
0.75
1.00
Proportion of genome in each compartment
gene expression log2(FPKM + 1)
0
5
10
15
A.1.1
A.1.2
A.2.1
A.2.2
B.1.1
B.1.2
B.2.1
B.2.2
CALDER_Spinal_cord

(SNIPER

(CALDER

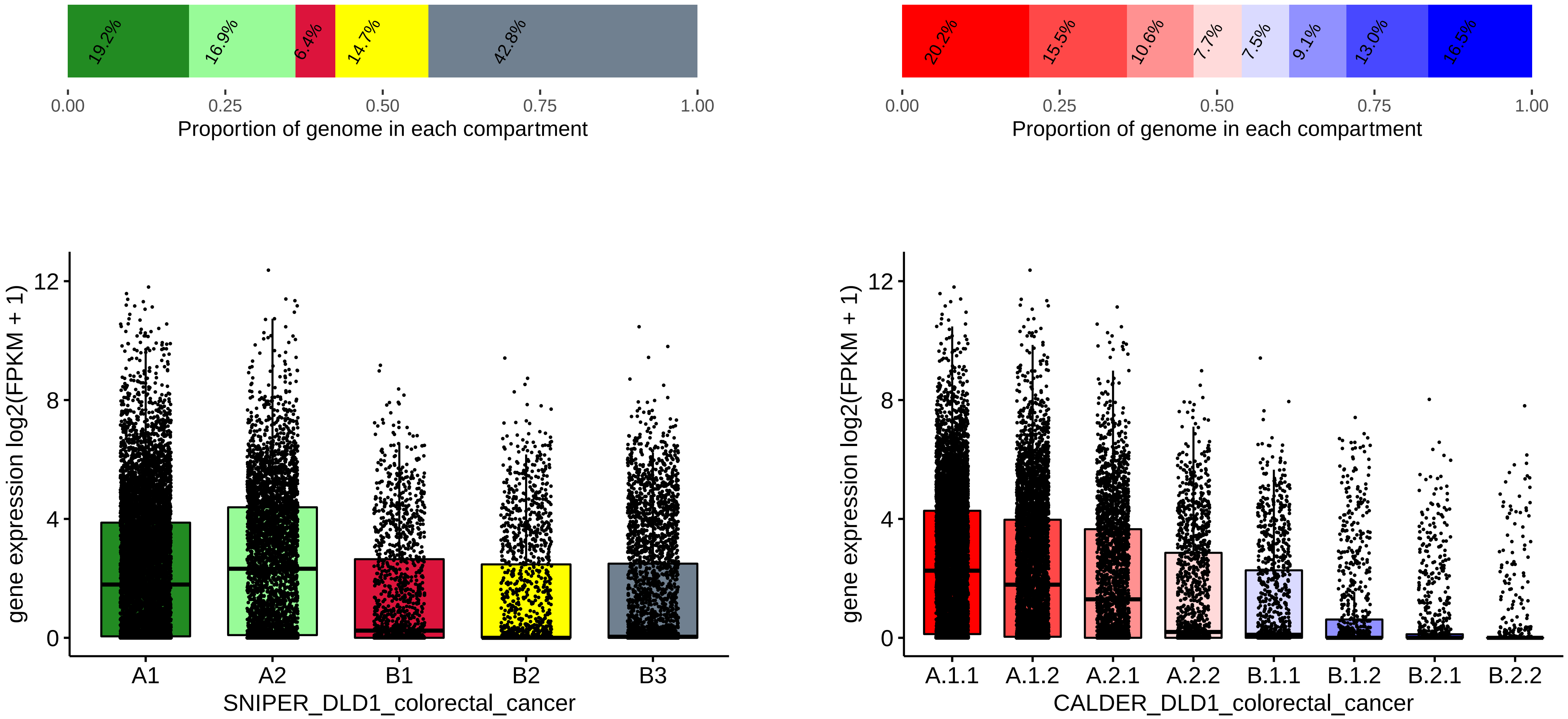

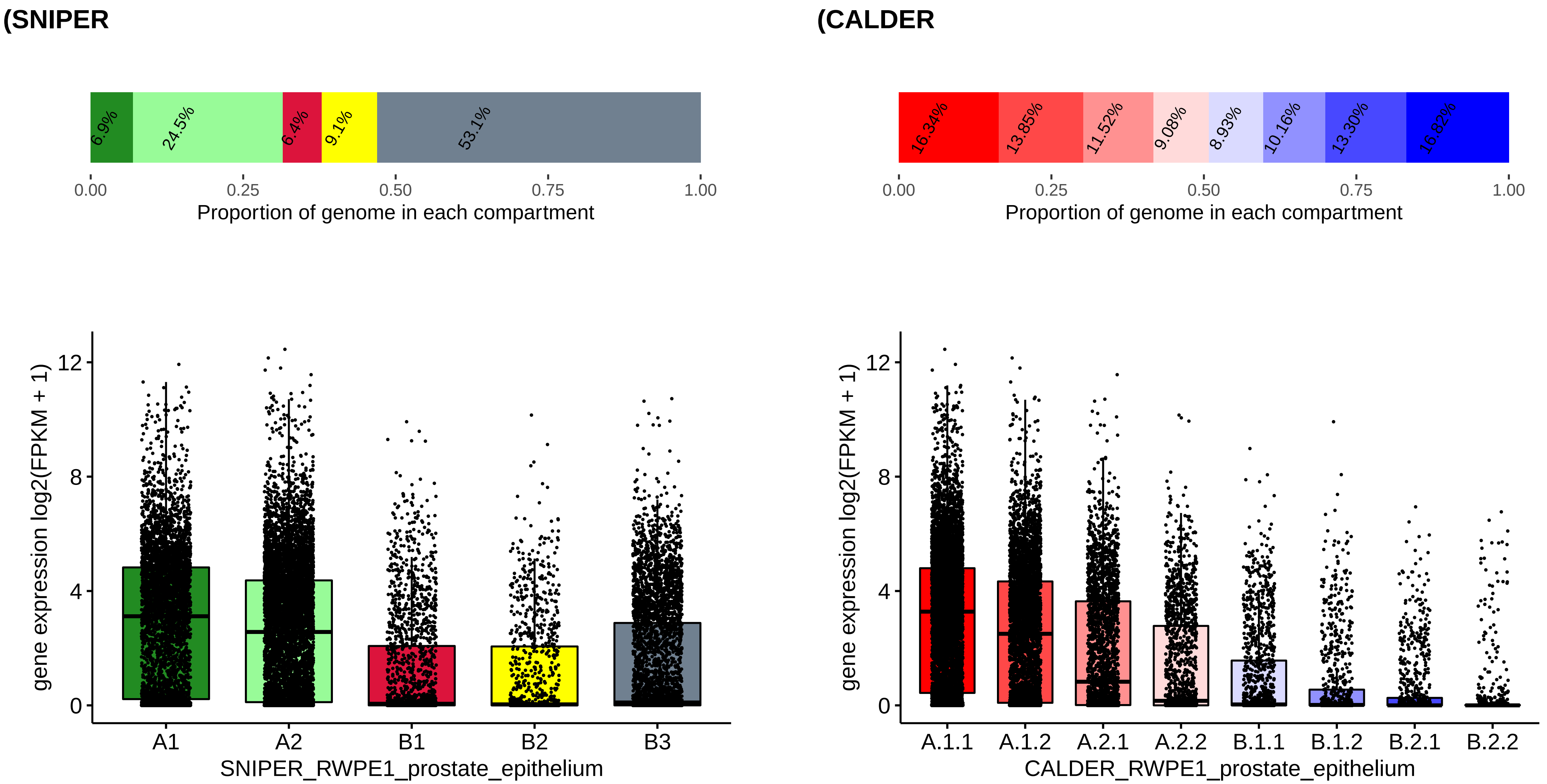

(SNIPER
6.9%
24.5%
6.4%
9.1%
53.1%
0.00
0.25
0.50
0.75
1.00
Proportion of genome in each compartment
gene expression log2(FPKM + 1)
0
4
8
12
A1
A2
B1
B2
B3
SNIPER_RWPE1_prostate_epithelium
(CALDER
16.34%
13.85%
11.52%
9.08%
8.93%
10.16%
13.30%
16.82%
0.00
0.25
0.50
0.75
1.00
Proportion of genome in each compartment
gene expression log2(FPKM + 1)
0
4
8
12
A.1.1
A.1.2
A.2.1
A.2.2
B.1.1
B.1.2
B.2.1
B.2.2
CALDER_RWPE1_prostate_epithelium

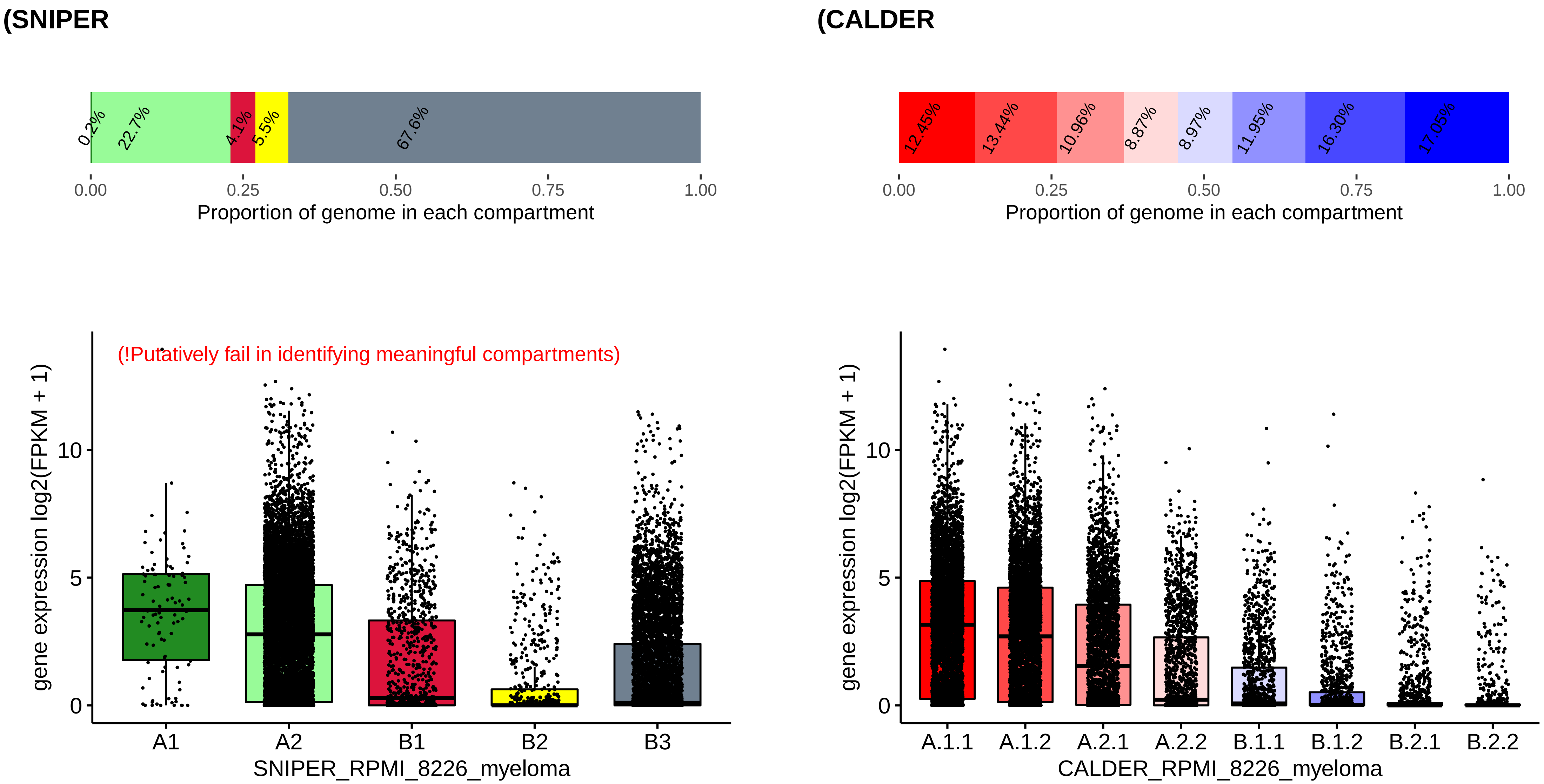
(SNIPER
0.2%
22.7%
4.1%
5.5%
67.6%
0.00
0.25
0.50
0.75
1.00
Proportion of genome in each compartment
(!Putatively fail in identifying meaningful compartments)
gene expression log2(FPKM + 1)
0
5
10
A1
A2
B1
B2
B3
SNIPER_RPMI_8226_myeloma
(CALDER
12.45%
13.44%
10.96%
8.87%
8.97%
11.95%
16.30%
17.05%
0.00
0.25
0.50
0.75
1.00
Proportion of genome in each compartment
gene expression log2(FPKM + 1)
0
5
10
A.1.1
A.1.2
A.2.1
A.2.2
B.1.1
B.1.2
B.2.1
B.2.2
CALDER_RPMI_8226_myeloma

(SNIPER

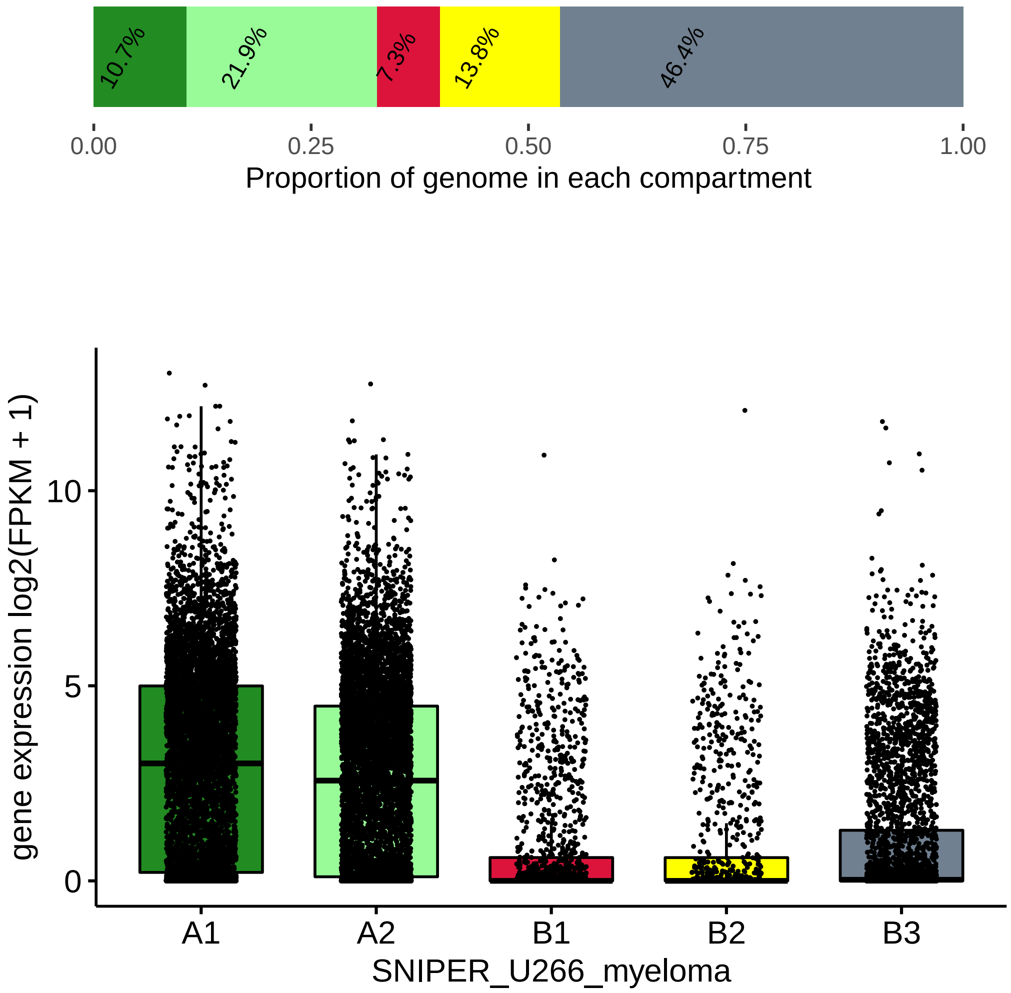


(CALDER

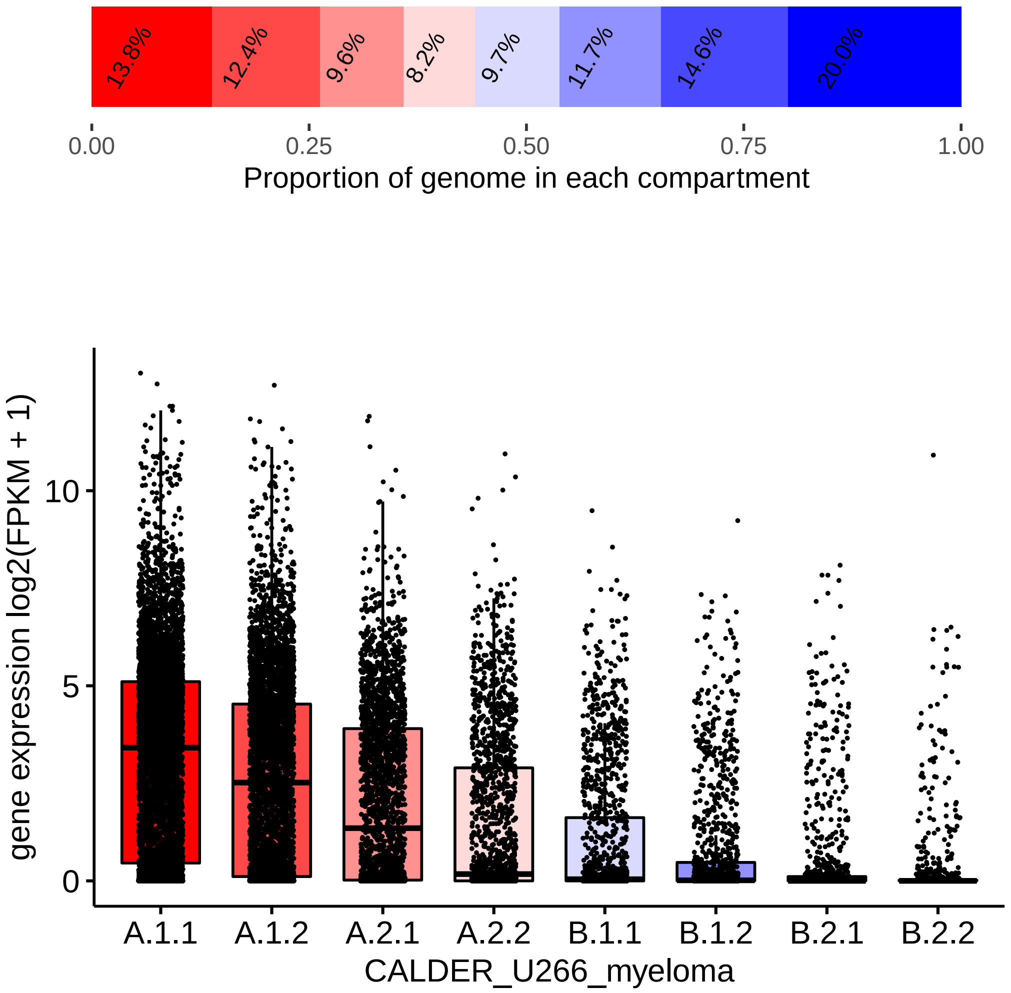

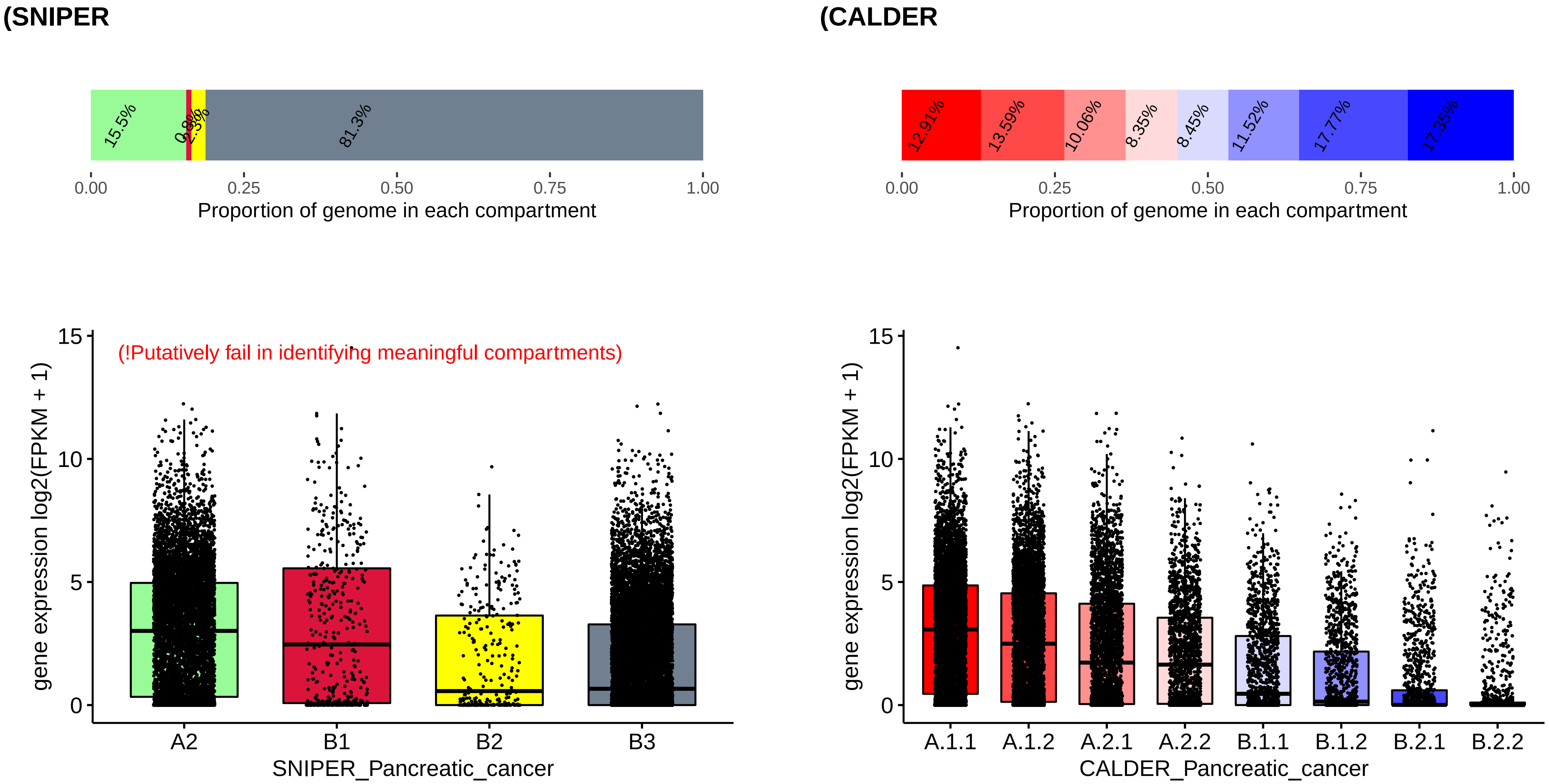
(SNIPER
15.5%
0.8%
2.3%
81.3%
0.00
0.25
0.50
0.75
1.00
Proportion of genome in each compartment
(!Putatively fail in identifying meaningful compartments)
gene expression log2(FPKM + 1)
15
10
5
0
A2
B1
B2
B3
SNIPER_Pancreatic_cancer
(CALDER
12.91%
13.59%
10.06%
8.35%
8.45%
11.52%
17.77%
17.35%
0.00
0.25
0.50
0.75
1.00
Proportion of genome in each compartment
gene expression log2(FPKM + 1)
15
10
5
0
A.1.1
A.1.2
A.2.1
A.2.2
B.1.1
B.1.2
B.2.1
B.2.2
CALDER_Pancreatic_cancer

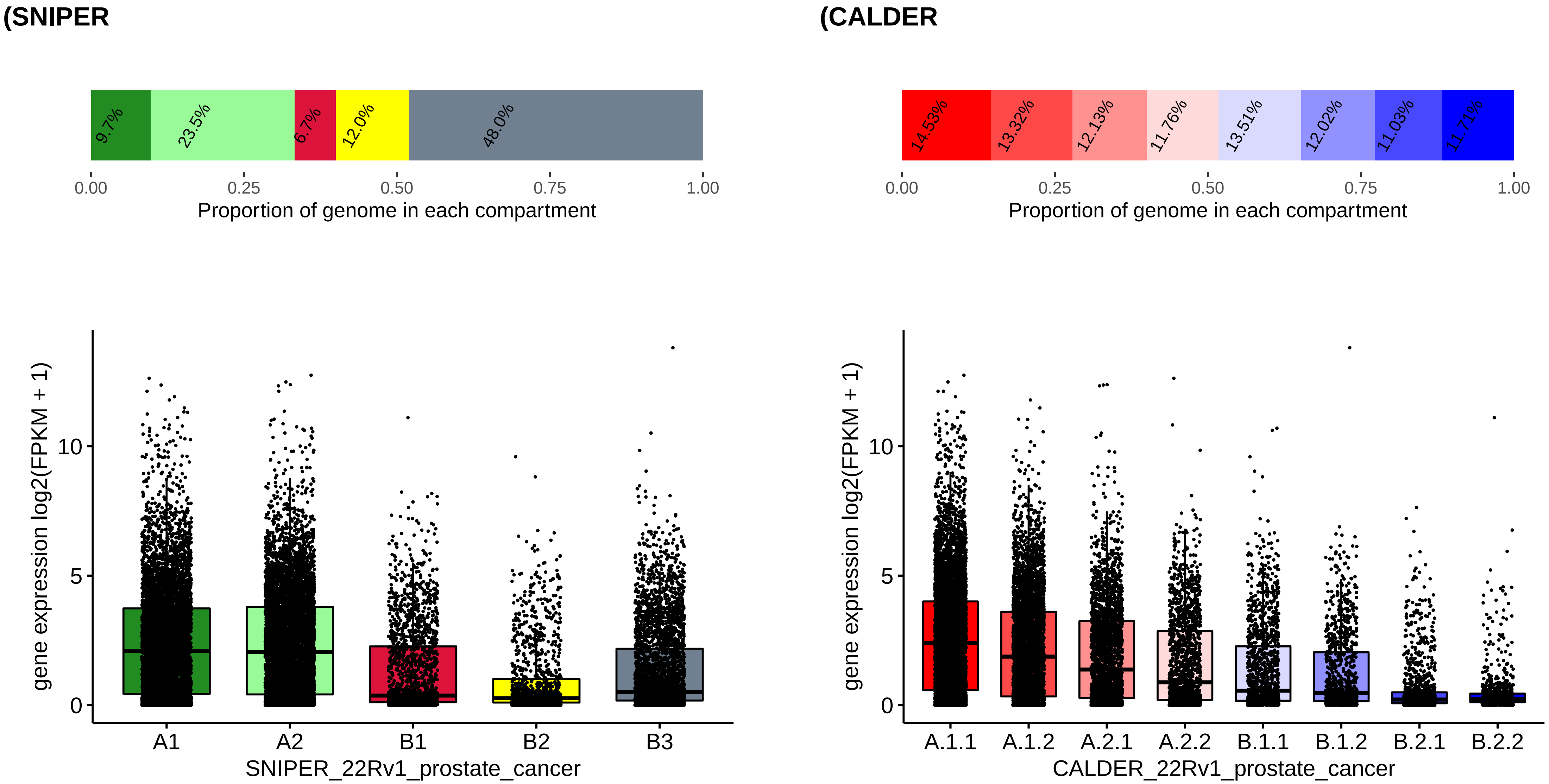
(SNIPER
9.7%
23.5%
6.7%
12.0%
48.0%
0.00
0.25
0.50
0.75
1.00
Proportion of genome in each compartment
gene expression log2(FPKM + 1)
0
5
10
A1
A2
B1
B2
B3
SNIPER_22Rv1_prostate_cancer
(CALDER
14.53%
13.32%
12.13%
11.76%
13.51%
12.02%
11.03%
11.71%
0.00
0.25
0.50
0.75
1.00
Proportion of genome in each compartment
gene expression log2(FPKM + 1)
0
5
10
A.1.1
A.1.2
A.2.1
A.2.2
B.1.1
B.1.2
B.2.1
B.2.2
CALDER_22Rv1_prostate_cancer

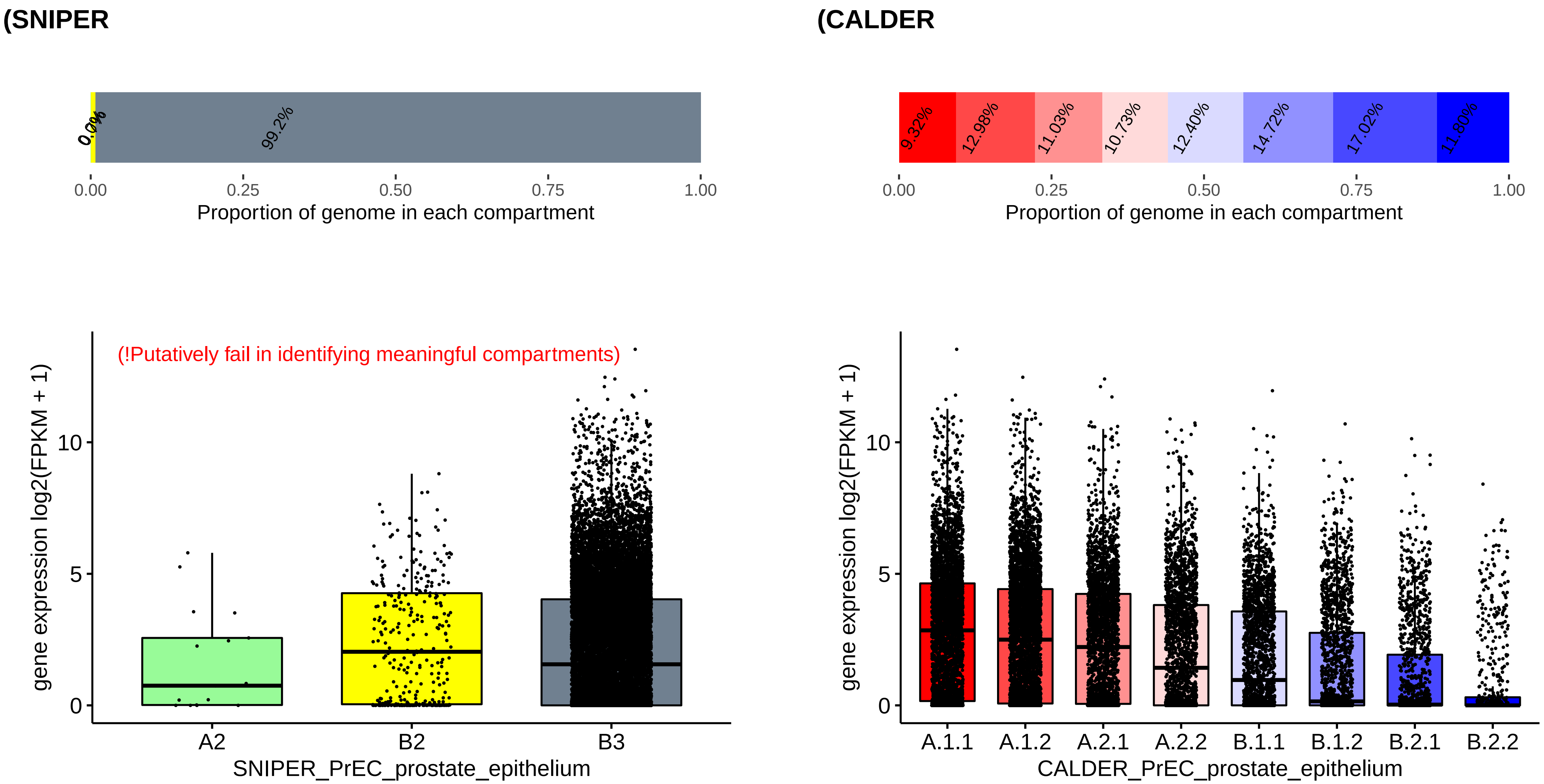

(SNIPER
0.0%
99.2%
0.00
0.25
0.50
0.75
1.00
Proportion of genome in each compartment
(!Putatively fail in identifying meaningful compartments)
gene expression log2(FPKM + 1)
0
5
10
A2
B2
B3
SNIPER_PrEC_prostate_epithelium
(CALDER
9.32%
12.98%
11.03%
10.73%
12.40%
14.72%
17.02%
11.80%
0.00
0.25
0.50
0.75
1.00
Proportion of genome in each compartment
gene expression log2(FPKM + 1)
0
5
10
A.1.1
A.1.2
A.2.1
A.2.2
B.1.1
B.1.2
B.2.1
B.2.2
CALDER_PrEC_prostate_epithelium
