# Supplementary material for: Systematic inference and comparison of multi-scale chromatin sub-compartments connects spatial organization to cell phenotypes
Source: Nat Commun. 2021 May 10;12:2439. doi: 10.1038/s41467-021-22666-3 (PMC8110550; doi:10.1038/s41467-021-22666-3)
Supplement: Supplementary file 8 — Supplementary Dataset 5 [file 41467_2021_22666_MOESM8_ESM.zip › 291893_0_merged_1609760806.pdf]

Systematic inference and comparison of multi-scale chromatin sub-compartments connects spatial organization to cell phenotypes

Yuanlong Liu[1,2,3], Luca Nanni[5], Stephanie Sungalee[2,4], Marie Zufferey[1,2,3], Daniele Tavernari[1,2,3], Marco Mina[1,2,3], Stefano Ceri[5], Elisa Oricchio[2,4], Giovanni Ciriello[1,2,3,*]

[1] Department of Computational Biology, University of Lausanne, Lausanne, Switzerland
[2] Swiss Cancer Center Leman, Lausanne, Switzerland
[3] Swiss Institute of Bioinformatics, Lausanne, Switzerland
[4] Swiss Institute for Experimental Cancer Research (ISREC) School of Life Sciences, EPFL
[5] Department of Electronics, Information, and Bioengineering, Politecnico di Milano, Milan, Italy

* Correspondence to: giovanni.ciriello@unil.ch

Chromatin compartmentalization reflects biological activity. However, inference of chromatin sub-compartments and compartment domains from chromosome conformation capture (Hi-C) experiments is limited by data resolution. As a result, these have been characterized only in a few cell types and systematic comparisons across multiple tissues and conditions are missing. Here, we present Calder, an algorithmic approach that enables the identification of multi-scale sub-compartments at variable data resolution. Calder allowed to infer and compare chromatin sub-compartments and compartment domains in >100 cell lines. Our results revealed sub-compartments enriched for poised chromatin states and undergoing spatial repositioning during lineage differentiation and oncogenic transformation.

In interphase, the chromatin is packaged into a hierarchy of three-dimensional (3D) structural elements (SEs) emerging from interactions and insulation of distinct DNA regions[1–3]. Hi-C technologies have allowed to quantify and computationally model such interactions to unravel chromatin spatial organization[4–6]. The formation of chromatin SEs is driven by two major mechanisms: loop extrusion of chromatin fibers mediated by CTCF and cohesin, and chromatin compartmentalization, which segregates chromatin regions with different patterns of histone acetylation and methylation[7]. Loop extrusion has been associated with the formation of topologically associating domains (TADs) and structural loops[8–10], whereas chromatin compartmentalization segregates the chromatin into spatial compartments[11,12] and compartment domains[10]. At a broad scale, chromatin segregates into two major compartments, one preferentially localized at the core of the nucleus and exhibiting high transcriptional activity (A compartment), and another localized closer to the nuclear lamina and enriched for repressed and gene-depleted chromatin (B compartment). Within each chromosome, DNA regions belonging to a given compartment are defined as compartment domains. Interestingly, recent experiments based on CTCF and cohesin depletion have shown that although often overlapping and sometime coincident, TADs and compartment domains are in fact distinct SEs[13]. Compartment domains from the same compartment preferentially interact among each other and, in Hi-C contact correlation maps, the alternance of A and B compartment domains generates a chessboard or "plaid" pattern reflecting enrichment or depletion of Hi-C interactions[4].

Computational inference of A and B compartments has been performed across multiple cell types and it showed, for example, that compartments are less conserved across cell types than TADs[5] and phenotypic changes are more frequently associated with compartment repositioning of a given genomic region than with structural disruption of loops or TADs[14]. Importantly, A and B compartments have been shown enriched for distinct histone modifications, which are consistent with the observed transcriptional activity. However, chromatin activity encompasses multiple states[15], some of which can only be captured through a more refined sub-compartmentalization[2]. It was previously proposed that the A and B dichotomy might not be sufficient to explain chromatin compartmentalization[16]. Indeed, up to 6 sub-compartments have been inferred by clustering inter-chromosomal interactions using a Gaussian Hidden Markov Model (GHMM)[2,10] and, recently, an intermediate compartment was characterized using Hi-C and imaging techniques in colorectal tumor samples[17]. Computational inference of sub-compartments has so far relied on inter-chromosomal contacts and, thus, it has been possible only for high-resolution experiments. For example, the GHMM approach was exclusively applied to the GM12878 cell line (4.9 billion read pairs). Machine learning-based imputation of Hi-C contacts has been used to enhance data resolution and allow sub-compartment inference in eight additional Hi-C experiments[18]. However, as we will show, this approach frequently fails to correctly infer sub-compartments when challenged with relatively low-resolution experiments or when patterns of inter-chromosomal interactions deviate significantly from the training dataset. As a consequence, the identification of chromatin sub-compartments and compartment domains remains unfeasible for the vast majority of available Hi-C datasets.

The Calder algorithm

Here we introduce an algorithmic approach that infers a complete hierarchy of compartment domains using exclusively intra-chromosomal interactions, which are more frequent than inter-chromosomal ones and thus alleviate the requirements on data resolution (**Fig. 1a**). Our approach consists of two main steps plus an optional one. First, it computes whole-chromosome contact similarities among genomic loci (Fisher's z-transformed correlations) and identifies compartment domains by segmenting each chromosome into regions having high intra-region similarity and low inter-region similarity. Next, compartment domains are clustered using a divisive hierarchical clustering approach exclusively based on their inter-domain contacts (3D-proximity) and ignoring their contiguity along the genome sequence (1D-proximity). Dendrograms generated for each chromosome are internally re-ordered, without disrupting the clustering structure, to match sub-compartments among different chromosomes. Lastly, a mixture log-normal distribution model can be applied to short-range contacts within each compartment domain to estimate the likelihood of nested sub-domains (see **Methods** for an in-depth description of the algorithm). The resulting compartment domain hierarchy describes how compartment domains group to progressively form sub-compartments at various scales. As such, sub-compartments can be explored at multiple levels of granularity, as each internal node of the dendrogram can in principle be thought as a sub-compartment comprising all the domains descending from it. For analytical purposes, compartment domains within each chromosome are assigned a *normalized rank* between 0 and 1, which identifies

their position in the dendrogram (0 being the most inactive and 1 the most active compartment domain). Importantly, the position of a compartment domain in the dendrogram can vary across cell models since it depends exclusively on 3D-proximity with other domains. This is in stark contrast to previous approaches inferring TAD hierarchies[19–21], which invariably preserved TAD 1D proximity, i.e. two contiguous TADs along the genome sequence will also be contiguous in the inferred hierarchy. We called our approach *Calder* to draw a suggestive analogy between the non-static nature of compartment domain hierarchies and the mobile sculptures of Alexander Calder (**Supplementary Fig. 1a**).

Chromatin sub-compartments and compartment domains

First, we used Calder to infer chromatin compartmentalization in 7 cell lines analyzed by Hi-C at high data resolution (**Supplementary Table 1** and **Supplementary Data 1**). Note that, in the following, we will refer to the total number of reads of an Hi-C experiment as its *data resolution*, which is related but distinct from the Hi-C *map resolution*, which typically refers to the bin size of the Hi-C contact map. At the top of the hierarchy, Calder identified A and B compartments that were largely coincident with those determined using previous approaches[2,4,18] (**Supplementary Fig. 1b**). Descending the hierarchy, sub-compartments could be explored at different levels of granularity (**Fig. 1a**) and were independent of data normalization (**Supplementary Fig. 2a**). Given that chromatin compartmentalization is expected to be associated with histone modifications[7], we determined a biologically meaningful number of sub-compartments by analyzing chromatin immunoprecipitation and sequencing (ChIP-seq) data for 7 histone marks. We found that 8 sub-compartments were sufficient to capture significant changes of these histone modifications in all cell lines (**Fig. 1b**): 4 within the A compartment (A.1.1, A.1.2, A.2.1, A.2.2) and 4 within the B compartment (B.1.1, B.1.2, B.2.1, B.2.2). Histone modifications associated with active transcription were overall enriched in A.1.1, A.1.2, and A.2.1 sub-compartments, whereas the repressive chromatin mark H3K27me3 was enriched in the intermediate sub-compartments B.1.1 and B.1.2, and the heterochromatin mark H3K9me3 in B.2.1 and B.2.2 (**Fig. 1c** and **Supplementary Fig. 2b**). To infer chromatin activity states from histone modifications, we used the ChromHMM algorithm[22] and confirmed the associations between active states (e.g. active promoters and enhancers) and A.1.1-A.1.2 and between heterochromatin and B.2.1-B.2.2 (**Fig. 1d**). Interestingly, this analysis further highlighted the associations between intermediate sub-compartments and poised chromatin states, with poised promoters enriched in A.2.1-to-B.1.1 and H3K27me3-rich polycomb repressed chromatin in B.1.1-to-B.2.1 (**Fig. 1d**). These results show that the broad dichotomy into A and B compartments is insufficient to capture the diversity of chromatin states and their biological activity.

Next, we compared Calder 8 sub-compartments with those inferred using the few available approaches. First, we used the adapted K-means (AKm) approach, originally used to detect 3 sub-compartments[16], to infer k = 8 sub-compartments. However, AKm sometimes failed to call the desired number of compartments, a behavior already observed in a previous study[23]. Correlations between Akm compartment assignments and average histone mark intensities or mRNA expression were consistently lower than observed for Calder sub-compartments

(**Supplementary Fig. 3a**). Next, we compared Calder 8 sub-compartments to the previously proposed partition into 5-to-6 sub-compartments[2]: A1, A2, B1, B2, B3, and B4 (the latter however is specifically associated with a ~11MB region of Chr. 19 in GM12878). Although the original GHMM approach was applied only to the GM12878 Hi-C map, a Hi-C contact imputation approach called SNIPER[18] was later proposed to enhance data resolution in 5 additional Hi-C maps[2] (IMR90, HMEC, K562, HUVEC and HeLa) and infer 5 sub-compartments in these models. Sub-compartments inferred by Calder and SNIPER in these 6 cell lines were highly concordant (**Supplementary Fig. 3b**) although those identified by Calder exhibited a stronger association with histone modifications and transcriptional activity than the ones identified by SNIPER (**Supplementary Fig. 3a**). To test the robustness and versatility of the two approaches, we inferred sub-compartments in 38 cell lines representing diverse conditions and with different data resolution. Here, we found that SNIPER frequently called highly unbalanced sub-compartments (**Supplementary Fig. 4a**): in 50% of the cases at least one sub-compartment (most often A1) accounted for less than 1% of the genome, and in 8 cases the method could not identify all 5 sub-compartments (**Supplementary Table 2**). In these unbalanced cases, sub-compartment assignments did not correlate with transcriptional activity, further suggesting that these did not reflect the true chromatin compartmentalization (**Supplementary Data 2**). Vice versa, Calder always called balanced sub-compartments (**Supplementary Fig. 4a**) exhibiting high correlation with transcriptional activity (**Supplementary Data 2**). In particular, we found that SNIPER failed to call sub-compartments in cell lines where inter-chromosomal contacts exhibited different frequency distributions from what was observed in the GM12878, which SNIPER uses as training dataset. This was most evident in cell lines that exhibited chromosomal translocations, which are frequent in cancer (e.g., see **Supplementary Fig. 4b**). Furthermore, by inferring sub-compartments at high map resolution (10kb bin), Calder revealed fine sub-compartmentalization of the chromatin that could not be captured by SNIPER (100kb bin). Representative examples showed that a shift to intermediate sub-compartments, such as B.1.1 or A.2.2, was associated with increased H3K27me3 even in regions smaller than 100kb, or that transitions from A.1.1 to A.1.2 and A.2.2 was associated with decreasing H3K27ac peak intensity (**Fig. 1e**). Overall, these results confirmed that histone mark heterogeneity is tightly linked to chromatin sub-compartmentalization, and Calder can robustly reveal this association across multiple experiments and with greater detail than previous approaches.

At the bottom level of the hierarchy, compartment domains were clustered and ranked based on their 3D inter-domain contacts and, indeed, the distance between two domains was anti-correlated with the mean number of inter-domain interactions (**Supplementary Fig. 5a**). Compartment domain ranks were highly associated with histone mark intensities. For example, markers of active enhancers (H3K27ac), promoters (H3K4me3), and transcribed regions (H3K36me3) were all positively correlated with the ranks of chromosome 1 domains in GM12878 whereas, H3K27me3 showed significant anti-correlation (**Fig. 2a**). These trends were confirmed for all chromosomes (**Fig. 2b**) and all cell lines used for testing (**Fig. 2c** and **Supplementary Data 1**). Whereas several TAD callers have been proposed in the literature[24], compartment domain boundaries have been simply inferred as genomic positions where a change of A/B compartment or sub-compartment occurs[10] or as positions where there is a switch of contact propensity towards either A or B compartments[25]. We obtained compartment

domain boundaries using these approaches in the GM12878 cell line, based on available sub-compartment annotations[2]. On the one hand, compartment domain boundaries inferred by Calder covered 92-93% of boundaries determined based on these annotations and approaches (**Supplementary Fig. 5b**). On the other hand, Calder inferred a much larger number of boundaries, consistent with its ability to infer a finer chromatin compartmentalization than other approaches. To explore the features of boundaries identified exclusively by Calder, we computed fold-changes of histone mark intensity between the contiguous domains that they separated. Histone mark fold-changes were significantly higher than expected at these boundaries (**Supplementary Fig. 5c**), supporting that they delimited domains within different sub-compartments. To further explore the features of compartment domain boundaries inferred by Calder, we assigned each boundary to one of 4 classes based on how far apart in the dendrogram were the domains it separated (**Fig. 2d**). Histone mark fold-changes were greater when the two domains were assigned to distinct A and B compartments (Level 1) and decreased as the domains were assigned to more similar sub-compartments, independently of CTCF binding status (**Fig. 2e**). Boundaries separating nested domains (Calder Step 3 – **Fig. 1a**) exhibited the lowest histone mark fold-changes (**Fig. 2e**). Interestingly, the enrichment of CTCF and cohesin binding also decreased from level 1 to level >3 compartment domain boundaries, but it was greatest at nested boundaries (**Supplementary Fig. 5d**). These results suggest that nested domain boundaries are not compartment domain boundaries, but they are more likely to be associated with structural loops or TADs.

To further assess the overlap and differences between compartment domain boundaries inferred by Calder and TAD boundaries inferred by other callers, we compared our results in the GM12878 cell line with those obtained using TopDom[26] and Arrowhead[2], two top performing TAD callers as evaluated in a recent benchmarking study[24]. Between 50% and 70% of Calder compartment domain boundaries were within one bin distance (+/- 10kb) from TAD boundaries inferred by TopDom or arrowhead, but this percentage increased to 70-85% for nested domain boundaries (**Fig. 2f**). Genome-wide, boundaries inferred by exclusively by one of these tools exhibited different features. Calder-specific boundaries were associated with greater changes of histone mark intensities than arrowhead- or TopDom-specific boundaries (**Fig. 2g**). Conversely, even though all boundaries identified by only one method were less enriched for CTCF and cohesin binding, this trend was particularly evident for Calder-specific boundaries (**Supplementary Fig. 5e**). Visual inspection of genomic regions where Calder and TopDom identified different boundaries further confirmed that TopDom boundaries delimited domains exhibiting local contact enrichment consistent with loop domains or TADs (**Fig. 2h** – left-side map). Conversely, Calder boundaries were aligned with the 'plaid' pattern characteristic of chromatin compartments, (**Fig. 2h** – right-side map). Lastly, we analyzed independent Hi-C cohorts generated before and after degradation of CTCF[9] or deletion of the cohesin loading factor Nipbl[11]. Across both comparisons, we observed a consistent loss of nested domain boundaries, with 44% and 37% fewer nested boundaries upon CTCF and cohesin removal, respectively (**Supplementary Fig. 5f**). Interestingly, after Nipbl deletion, we found that loss of nested boundaries was accompanied by an increased number of compartment domain boundaries inferred by Calder (**Supplementary Fig. 5f**), consistent with the fine compartmentalization reported after cohesin depletion[11]. The different effect on compartment

domain boundaries induced by CTCF and cohesin depletion was consistent with evidence that only cohesin loss impairs loop extrusion and lead to fine compartmentalization[7]. These results demonstrate that Calder specifically infers compartment domains, which are associated with histone post-translational modifications and, although often overlapping, are not coincident with TADs.

Compartment domain inference and repositioning across >100 cell lines

Consistent with the chromatin epigenetic status, compartment domains ranks correlated with transcriptional activity (**Supplementary Fig. 6a**). As transcriptional activity is highly variable among different cell types, we wondered whether this variability is reflected in spatial repositioning of compartment domains. We first tested this hypothesis by comparing the position of the domain containing the B-cell marker CD20 (gene name *MSA41*) in lymphoblastoid cells (GM12878), where the gene is highly expressed, and in lung fibroblasts (IMR90), where the gene is silenced (**Fig. 3a**). Calder inferred a drastic repositioning of the *CD20*-containing domain between IMR90 (rank: 0.0035, B.2.2 sub-compartment) and GM12878 (rank: 0.92, A.1.1 sub-compartment). Conversely, we did not observe any repositioning for the domain containing the *OSBP* gene ($rank_{IMR90}$: 0.92, $rank_{GM12878}$: 0.98, A.1.1 sub-compartment) (**Fig. 3b**), which is proximal to *CD20* in the genome sequence but similarly expressed in the two cell lines (**Fig. 3a**). *CD20* repositioning was associated with a different enrichment of Hi-C contacts at its locus in GM12878 and IMR90, whereas contact frequencies were similar at the *OSBP* locus (**Fig. 3c**). Notably, previous approaches to infer TAD hierarchies always preserved 1D-proximity, i.e., genome sequence contiguity, and thus cannot be used to capture repositioning events (**Supplementary Fig. 6b**).

To explore chromatin sub-compartments and compartment domain repositioning across a broader and more diverse set of conditions, we collected Hi-C data for 114 cell lines and tissue samples (**Supplementary Table 1, Supplementary Fig. 6a, Supplementary Data 1**). To assess the robustness of our approach to Hi-C data and map resolution, we analyzed 8 cell lines using different bin sizes (10kb and 40kb) and 5 down-sampled versions of the GM12878 interaction map, retaining only 50%, 20%, 10%, 1% and 0.5% of its total number of contacts. Overall, we clustered 127 Hi-C datasets based on Calder sub-compartment (n = 8) assignments for each bin (**Supplementary Data 3**). Cell lines clustered largely based on their lineage instead of resolution (**Fig. 3a,b** and **Supplementary Fig. 6b**), as shown by downsampled or differentially binned contact maps of the same cell line that always clustered together (**Fig. 3c**). For example, B-lymphoblastoid GM12878 contact maps (**Fig. 3c** - green cluster) and cancer cell line models of B-cell malignancies (**Fig. 3c** - red cluster) formed two separate but contiguous clusters, and together with other B-lymphocytes were characterized by the repositioning of the CD20-containing domain to the active compartment (**Supplementary Fig. 6c**). Other lineage-associated clusters included a stem-like cluster comprising cardiac and neural progenitor and embryonic stem cells (**Fig. 3c** - blue cluster), as well as two separate clusters comprising normal epithelial and cancer cells (e.g. breast and prostate normal and cancer cell lines – **Fig. 3c** – pink and purple clusters, respectively). A lineage-independent cluster was found for a set of low data resolution Hi-C experiments generated from tissue specimen (**Fig. 3c**). Even though other Hi-C

maps at similar resolution clustered based on their lineage, we cannot exclude the possibility that our results were here affected by low read counts and/or other technical issues. Previous approaches used the first principal component of the contact enrichment correlation matrix (PC1) either to rank genomics bins within a chromosome (based on the values of PC1) or to determine A and B compartments (based on the sign of PC1). Clustering of the Hi-C datasets based on PC1 values was largely dependent of data and map resolution, failing to recover lineage associated clusters and clustering apart even Hi-C profiles from the same cell line (**Supplementary Fig. 7a,b**). Clustering using the sign of PC1 (i.e. A/B compartment calls) was less sensitive to resolution, but still could not recover lineage associated clusters to the same extent of the 8-sub-compartment classification inferred by Calder (**Supplementary Fig. 7a,c**). Lastly, to further corroborate the relevance of intermediate compartments, we compared Hi-C maps from stem-like cell lines and fully differentiated tissues, using exclusively genomic bins that were assigned to the same compartment (A or B) in all cell lines, but that differed at the level of sub-compartments. Clustering based on these bins perfectly separated stem-like models from differentiated tissues (**Supplementary Figure 7d**).

Using Shannon's information entropy (S), we examined which regions were found in the same sub-compartment across multiple cell lines (low entropy) and which instead were frequently repositioned (high entropy). The overall *compartment entropy* was significantly lower than expected by chance (**Supplementary Fig. 8a**), and it was higher in cancer than in normal cell lines (**Supplementary Fig. 8b**), suggesting that tumor molecular heterogeneity is also reflected in structural heterogeneity. Low-entropy bins were almost exclusively assigned to the most extreme compartments, i.e. B.2.2 and A.1.1. In contrast, intermediate sub-compartments only appeared in bins with high entropy values (**Fig. 3d**), indicating that genomic regions in an intermediate sub-compartment in one cell line are the more likely to be repositioned in other cell lines. Among the 114 cell lines that we analyzed, on average 49-54% of regions in A.1.1 or B.2.2 in a given cell line changed sub-compartment in another, as opposed to 80-85% of regions in the intermediate sub-compartments (A.2.1 to B.1.2) (**Supplementary Fig. 8c**). Protein-coding genes located in genomic regions of low entropy ($S < 0.15$, $n = 532$) were significantly enriched for fundamental cellular processes, such as protein transport, localization, and phosphorylation, RNA processing and metabolism, and cell cycle (**Fig. 2e** - top). Vice versa, high entropy genes ($S > 0.95$, $n = 315$) were significantly enriched for cell differentiation, development, and morphogenesis (**Fig. 3e** – bottom and **Supplementary Table 3**). To explore how lineage differentiation is reflected in compartment repositioning, we examined Hi-C maps of H1 human embryonic stem cells and 3 H1-derived lineages. Compartment hierarchies exhibited extensive compartment changes. However, in each comparison, only a minor fraction corresponded to A to B (or B to A) switches, whereas in all comparisons more than 50% of the genome exhibited sub-compartment repositioning (**Fig. 3f**). Overall, these results suggest that sub-compartments repositioning is frequent and potentially associated with lineage commitment and cell differentiation.

Lastly, we investigated sub-compartment repositioning in malignant transformation and analyzed 3 relatively homogeneous groups of cell lines including normal and cancer cells derived from breast, prostate, and pancreatic tissues (**Fig. 4a**). Sub-compartment repositioning

between cancer and normal cells was associated with changes in gene expression (**Supplementary Fig. 9a**) and these were on average higher with greater repositioning. By focusing on common sub-compartment repositioning events between normal and cancer cell lines (**Supplementary Table 4**), we found a shift from active to inactive compartment of the Forkhead box O transcription factor *FOXO1*, which has been frequently associated with tumor suppressive functions[27,28]. The *FOXO1* locus was in A.1.1 or A.1.2 in all normal cells and it shifted towards intermediate and inactive compartments in cancer cell lines (**Fig 4b**). Although it was not genetically altered in these cells[29], *FOXO1* repositioning was associated with loss of H3K27ac upstream of the gene (**Fig. 4c**) and down-regulation of *FOXO1* mRNA expression (**Fig. 4d**). Interestingly, by analyzing large human tumor cohorts profiled by The Cancer Genome Atlas[30] (TCGA), we found that *FOXO1* was downregulated in the vast majority of human breast, prostate, and pancreatic tumors (**Fig. 4e**) despite being only rarely target of genetic alterations in these tumor types: ~8% in prostate, <2% in breast, and never in pancreatic cancer (source cBioPortal[31]). To verify that sub-compartment repositioning was effectively associated with modified 3D-proximity to other domains, we analyzed intra-chromosomal contact frequencies between the *FOXO1*-containing compartment domain and other compartment domains that remained in the same sub-compartment in normal and cancer cell lines (**Fig. 4f**). In normal cell lines, *FOXO1* interacted more frequently with regions in active than with regions in inactive sub-compartments, but this trend was reversed in cancer cells (**Fig. 4g**), supporting a physical repositioning of the *FOXO1* locus in the chromatin 3D structure. Sub-compartment repositioning events hence allow to map transcriptional changes to modified chromatin interactions, pinpointing associations between gene expression and spatial organization.

Overall, Calder allows to robustly identify chromatin sub-compartments across a variety of cell types and conditions, enabling their systematic comparison and quantification of spatial repositioning to aid the understanding of the link between structure and phenotype.

Methods

Data and Code Availability

All Hi-C, ChIP-seq, and RNA-seq datasets used in this study are publicly available and described in the **Supplementary Table 1**. Chromatin domain hierarchies and compartment scores generated by CALDER for all 127 Hi-C maps are available as **Supplementary Data 1** in a bed format.
The CALDER algorithm was implemented in R and it is available at https://github.com/CSOgroup/CALDER along with a detailed user manual and test dataset.

Generating Hi-C contact matrices

Hi-C intra-chromosomal contact maps (or matrices) were either generated from raw FASTQ files using Juicer tools[32] with default parameters or obtained from processed public data. Data sources are provided in Supplementary Table 1. The Knight-Ruiz (KR) method was used for

contact matrix normalization when the source data was not normalized. In cases when KR normalization failed to converge, the VR (vanilla coverage) normalization was applied. All Hi-C contact maps were binned at 40kb resolution. Contact maps of GM12878, HUVEC, IMR90, K562, NHEK, HMEC, KBM7, and HeLa were additionally analyzed using 10kb bins. In addition, for sub-compartment comparisons, SNIPER was run with a bin-size equal to 100kb and Adapted k-means with a bin-size equal to 1Mb. Contact maps from tamoxifen-control and cohesin-removed datasets were binned at 20kb. The 100kb, 1Mb or 20kb bin sizes were chosen to be consistent with the setting used in the study where each method was original proposed. Lastly, we generated six down-sampled contact maps for GM12878 at 40kb resolution by randomly retaining 50%, 20%, 10%, 1%, or 0.5% of the total contact reads. To reduce technical noise within regions of low mappability, matched rows and columns with more than 99% values equal to 0 were removed. For each processed contact map, we defined its *contact density* as the fraction of non-zero entries. Only autosomal chromosomes were analyzed.

<u>Analyzing ChIP-seq data of histone modifications</u>

Processed ChIP-seq value (fold change over control, pooled replicates) were retrieved from ENCODE (https://www.encodeproject.org/) or GEO (https://www.ncbi.nlm.nih.gov/geo/). We also processed H3K27ac for BT474 from publicly available raw sequencing data. FASTQ files were aligned to hg19 reference genome by bowtie2[33] version 2.3.4.3 with local alignment option. Peaks of H3K27ac enrichment compared to the corresponding input control were identified using MACS2[34] version 2.1.1. Extreme values, defined as those greater than the upper 0.999 quantile of each chromosome, were replaced with the 0.999 quantile. Data sources are provided in **Supplementary Table 1**.

<u>Quantification of gene expression</u>

Gene expression values at mRNA level for cell lines were quantified by FPKM (fragments per kilobase per million mapped fragments) and were retrieved from ENCODE or processed from publicly available raw FASTQ files using RSEM[35] v.1.3.1. For running RSEM, we set hg19 as the reference genome and used STAR[36] 2.7.0d for alignment. Other parameters were set as default. We also retrieved gene expression values for normal and cancer human samples from The Cancer Genome Atlas (TCGA) collected in the FireHose data repository (https://gdac.broadinstitute.org/). We used TPM (transcripts per million) as a measure of gene expression level for these samples. Data sources are provided in **Supplementary Table 1**.

<u>The CALDER Algorithm</u>

Step 1: Compartment Domain Calling

We defined compartment domains as consecutive bins having high intra-domain similarity while low inter-domain similarity, where the similarity is measured based on whole-

chromosome interaction pattern. Chromatin domains identified by CALDER have similar size and features as previously defined topologically associated domains (TADs) and can in practice being considered the same type of structural element. However, given the original definition of TAD was mainly based on short-range interaction intensity (e.g. within ~2MB), we refrain from using the same terminology. Identification of compartment domains proceeds through the following steps (**Supplementary Fig. 10a**).

1. Providing an intra-chromosomal contact matrix A, we generated an aggregated matrix A* if the bin size is smaller than 40kb, by compressing every 10 columns into a single column, thus the i_{th} row of A* represents the total contacts between bin_i and every 10 bins. If the bin size is ≥ 40kb we omit this step thus A* = A. The purpose of this step is to sum up contact values to reduce data sparsity for small bin size, thus, to allow applying our method to bin size up to 10kb.
2. A correlation matrix ρA was computed storing all pairwise correlations of the rows of A*. To enhance the correlation signals, we computed pairwise column correlations again from ρA to generate a second correlation matrix ρ_2A.
3. ρ_2A was transformed into a similarity matrix defined as sA = arctanh(ρ_2A). The function arctanh was chosen such that correlations with absolute value greater than ~0.3 have a greater amplification ratio. This is also commonly referred to as Fisher z-transformation, which the converted correlation score is approximately normally distributed.
4. Lastly, we called compartment domain boundaries separating regions having high intra-region values and low inter-region values. To this purpose, we used the same strategy proposed in the TopDom algorithm applied to the sA matrix (instead of the Hi-C contact matrix).

Step 2: Deriving the hierarchy of chromatin domains
Given *k* chromatin domains identified at Step 1, we derived a clustering dendrogram based on the similarity of whole-chromosome inter-domain contacts. To build a hierarchy of chromatin domains, we proceed through the following steps (**Supplementary Fig. 10b**).

1. First, we computed 4 raw trend matrices T^m, with m = 1,…,4, each of dimension (k-m) × k, to summarize the plaid pattern commonly observed in the Hi-C contact matrix and that reflects compartmentalization. Values of T^m are defined as: $T^m(i,j) = 1$ if the mean contact value between bins in domain *i* and in domain *j+m* is greater than the mean contact value between bins in domain *i* and bins in domain *j* (thus indicating enrichment of contacts); otherwise $T^m(i,j) = 0$ (depletion of contacts). These 4 trend matrices were concatenated by rows to form a combined trend matrix T. A correlation matrix ρT was computed storing all the pairwise correlations of the columns of T and then converted to a score matrix sT using arctanh transform. Although a single raw trend matrix (m=1) can also be used to compute the correlation in this step, we found the concatenation of multiple matrices can increase the correlation signals, however the marginal gain is negligible when the number if greater than 4.

2. Next, we used the first 10 principal components (PCs) of sT to perform a divisive hierarchical clustering. Starting from the complete set of compartment domains, we iteratively split a set into two sub-sets using k-means (k=2), until each compartment domain is on a separate set. PC values can be re-scaled by a factor w, to give different weight to different PCs. Given the first PC (PC_1) was the most associated with chromatin compartmentalization (similarly to the PC_1 of normalized Hi-C maps[4,37]), in the analyses presented in this manuscript, we set w=1 for PC_1 and w=0.25 for all other PCs. We chose the first 10 PCs in this step as we observed the marginal variance explained by each additional PC is negligible.

3. Lastly, since there are multiple possible ways to order the dendrogram branches without disrupting its structure, we sought a consistent ordering among chromosomes reflecting chromatin compartmentalization.
    a. Given the sign of PCs is arbitrary, we fixed the sign of PC_1 such that it correlates with gene density (gene density is expected to be higher in A than in B compartment).
    b. Then we derived the non-linear major axis of the point cloud (each point represent one domain) formed by PC_1 and PC_2 using loess regression, i.e. loess(PC_2 ~ PC_1), and projected each data point, i.e. domain, [PC_1(x),PC_2(x)] to this major axis.
    c. To each domain we assigned a score *z* based on its ranking *j* of this major axis: $z_j = 0$ if $j = 1$; and $z_j = \sum_{i=2}^{j} \sqrt{(x_i - x_{i-1})^2 + (y_i - y_{i-1})^2}$ if *j* > 1, which is the Euclidean distance of between each pair of adjacent domains along this axis.
    d. Lastly, we ordered the branches of the dendrogram such that the branch on the right side should have a greater average *z* value than the branch on the left side, for any sibling branch pair.

The left and right branch at the top level of the ordered dendrogram were labelled as B and A respectively. For a sub-branch X, its left child branch was labelled as X.2 and right child branch as X.1. The chromatin domain ranks were derived from the order of the domains in the final dendrogram and normalized by the total number of chromatin domains in that dendrogram.

Step 3: Calling for nested sub-domains

A stochastic approach was developed to call nested sub-domains within each compartment domain exclusively based on short-range contacts. The basic idea is to determine whether a hierarchical structure of smaller domains can be further inferred from local contact patterns within a given compartment domain. In this step of the CALDER (and unlike what is done in the previous step) hierarchies of nested domains contained in a domain are anchored to the genome sequence, i.e. adjacent nested domain in the hierarchy are also contiguous along the genome sequence. This step can be split into two major tasks: 1) finding the best-fitting dendrogram D; 2) trimming D to get meaningful nested domains.

1. **Finding the best-fitting dendrogram.** We assume the contact probability between bins from two sibling domains in the dendrogram D follows a mixture log-normal distribution with parameter $\boldsymbol{\theta}_k = \{\alpha_k, \mu_k, \sigma_k\}$

$$p_{mix}(x|\alpha_k, \mu_k, \sigma_k) = \alpha_k p_0(x) + (1 - \alpha_k) p_1(x|\mu_k, \sigma_k)$$

   where $p_0(x)$ is the density function of a degenerate distribution which has P(X=0) = 1; $p_1(x)$ is the density of a log-normal distribution which has $\ln(X) \sim N(\mu, \sigma^2)$.
   Given the contact matrix A, the likelihood function of the dendrogram and the associated parameters is given by

$$L(D, \{\boldsymbol{\theta}_k\}|A) = \prod_{k \in \{\text{all sibling pairs}\}} p_{mix}(A|\boldsymbol{\theta}_k)$$

   We computed the best-fitting dendrogram and corresponding parameters using maximum likelihood estimation

$$\{D; \{\boldsymbol{\theta}_k\}\} = \text{argmax}_{D;\{\boldsymbol{\theta}_k\}} L(D, \{\boldsymbol{\theta}_k\}|A),$$

   which can be computed via dynamic programming and has a computation complexity of $O(n^3)$, where n is the number of bins in the chromatin domain under investigation (**Supplementary Fig. 10c** - left).

2. **Trimming the dendrogram to get meaningful nested domains.** Given the best-fitting dendrogram D, we extracted meaningful nested domains by splitting iteratively a domain into sub-domains as described below.

   a. First, we compute the observed vs. expected contact matrix A_{oe} to eliminate distance-bias of the contact matrix A_{oe} is defined as the ration between the contact maps A and E, where E is the expected contact matrix with:

$$E_{i,j} = mean(A_{j,k} \text{ for all } |j - k| \text{ equal to } |i - j|).$$

   b. A_{oe} within a given domain is split into four parts: **A:** the triangle delimited by the off-diagonal corner of the domain and the two off-diagonal corners of the candidate nested domains; **B:** the triangle comprising the contacts between the two nested domains that are not included in (a); **C-D:** contacts within the two nested domains (**Supplementary Fig. 10c** - right).

   c. Finally, a given split was accepted if 1) contacts within **A** were significantly higher than those in **B** and 2) contacts in **C** and **D** were significantly higher than contacts in **B**. Contact differences are tested using a one-tailed Wilcoxon test and considered as significant if p-value < 0.05 and $\Delta mean$ between the compared

regions is greater than 0.1 (0.1 is equivalent to 10% of relative difference given that the expected mean in any region of the distance corrected matrix A_{oe} is 1) .

The above procedures result in a trimmed dendrogram representing nested sub-domains in a compartment domain.

Number of compartments required to capture chromatin epigenetic pattern variation

To determine the number of compartments that is sufficient to explain the chromatin epigenetic pattern variation, we proceed as follow:

- For each histone mark, we first log-transformed (ln(x+1)) bin-level ChIP-seq intensity values and took the mean of these for each chromatin domain.
- Next, given two sibling compartments in the domain hierarchy with mean intensity values $X=\{x_1,\ldots,x_n\}$ and $Y=\{y_1,\ldots,y_m\}$ and overall mean $\mu(X) > \mu(Y)$, we defined the effect size $\Delta\mu$ as the maximum possible value such that $\mu(X)-\Delta\mu$ is significantly greater than $\mu(Y)$ (one-tailed t-test, significance threshold $\alpha=0.001$).
- Mean intensity values of two adjacent compartments were considered as significantly different if the median $\Delta\mu$ of all 22 chromosomes was above a threshold γ. To penalize large number of compartments that did not lead to high gains in terms of explained variance (similar to what is done in a penalized regression), we used progressive thresholds: $\gamma = 0.05$ for 2 and 4 compartments, $\gamma = 0.1$ for 8 compartments, and $\gamma = 0.2$ for >8 compartments.

We performed this test for each histone mark starting from the top (A/B compartment) and iteratively on lower levels of the hierarchy until no significant differences were reached. The number of compartments corresponding right before the stopping step was retained as the minimal number of compartment sufficient to explain epigenetic pattern variation.

Comparison with (sub-)compartments identified by SNIPER and Adapted K-means

We applied SNIPER on 38 Hi-C datasets at the resolution of 100kb. SNIPER is a machine learning-based method which first learned the relation between the inter-chromosomal contact pattern and compartment annotation of (Rao et al. 2014)[2], then predict five compartments (labeled as A1, A2, B1, B2, B3) of a new dataset using the new inter-chromosomal contact pattern. The author provided five trained models for different down-sample ratios (2%, 3%, 4%, 5%, 10%). We chose the down-sample ratio that best matches the observed ratio between a given dataset and the training dataset of GM12878 (ratio is defined as total inter-chromosomal contacts of the given dataset divided by total inter-chromosomal contacts of the GM12878 dataset). We also applied Adapted k-means on 7 Hi-C datasets (GM12878, Hela, HMEC, HUVEC, IMR90, K562 and NHEK), using 1Mb bin size as specified in the original paper. We set *k=8* with the aim to identify the same number of sub-compartments as Calder. We ordered the inferred

sub-compartments based on the average H3Kme3 intensity of each sub-compartment (By default, Adapted-kmeans does not give a label or rank to each compartment it infers).

Comparison with domain boundaries defined from other approaches

Compartment domain boundaries have been simply inferred as genomic positions where a change of A/B compartment or sub-compartment occurs[10], or as positions where there is a switch of contact propensity with A/B compartments[25]. For the first approach, we retrieved compartment domain boundaries based on the publicly available sub-compartment annotation from[2]. For the second approach, we computed for each 10kb bin the average distance-corrected contact intensity with A or B compartment. An A-B index was defined as the difference between the A/B intensity. Compartment domain boundaries were retrieved at positions where a switch of the sign (+/-) of A-B index occurs. Switches that occupy only 1 bin were considered as noise and omitted. To compare boundaries identified by these approaches or Calder, we considered two boundaries as overlapping if they are less than 100kb apart (100kb was the resolution of sub-compartment annotation from[2]).

We also applied Arrowhead[2] and TopDom[26] to the Hi-C map of GM12878 at 10kb bin size to call TADs, using default parameters. Two boundaries were considered as overlapping if they are less than 40kb apart.

Inferring genome sequence dependent hierarchies using TADpole

We applied TADpole to the Hi-C data of GM12878 binned at 40kb to derive genome sequence dependent hierarchies, for the purpose to compare with Calder's genome sequence independent hierarchies as shown in **Supplementary Fig. 6b**. Parameters for TADpole was set as *max_pcs=50*, *bad_frac=0* and *centromere_search=FALSE*.

Computing the *chromatin entropy*

Given the 8-compartment model, we defined the Shannon's information entropy of a bin *b* as:

$$S_b = -\frac{1}{\ln(8)}\sum_{j=1}^{8} P_j \ln(P_j)$$

where P_j is the frequency with which *b* is assigned to compartment *j* across the 114 datasets. The normalizing factor ln(8) ensures that S is in the [0,1] range. We defined the distribution of entropy values for all bins of the genome as *chromatin entropy*. To estimate the expected chromatin entropy, we performed 1000 permutations of compartment labels. In each permutation, we randomly shuffled the compartment labels of each bin, within each dataset independently. Chromatin entropies computed from each permutation were aggregated to derive the overall random distribution.

Enrichment of histone mark intensities and ChromHMM states at compartments

For each histone mark, mean intensity values for each chromatin domain were computed. Next, we determined the enrichment of these values within a given compartment (8-compartment model) as the $\log_2$-transformed ratio between the median value in the compartment and the overall median value.

To determine the enrichment of ChromHMM states in each compartment, we used the 15-state definition reported in:
http://genome.ucsc.edu/cgi-bin/hgTrackUi?db=hg19&g=wgEncodeBroadHmm
To each genomic region associated to a given state by ChromHMM, we assigned the domain rank value and compartment label of the chromatin domain overlapping with that region. If a region was covered by multiple chromatin domains, we assigned to that region the mean domain rank and the "least active" compartment label, i.e. the label closest to B.2.2. As a result, each of the 15 ChromHMM states were associated with a vector of domain ranks and a vector of compartment labels. To determine the enrichment of a given compartment label in a ChromHMM state, we computed the number of occurrences of each compartment label for regions annotated with a given ChromHMM state S, and divided these numbers by the expected values obtained by multiplying the number of regions in S by the vector of compartment label frequencies across the entire genome. Genomic regions that did not overlap with any chromatin domain were discarded in this analysis.

Gene set enrichment analysis

Coding genes located in genomic regions of low entropy ($S < 0.15$, $n = 532$) and of high entropy ($S > 0.95$, $n = 315$) were tested for enrichment of Gene Ontology terms in the Molecular Function and Biological Process categories using the mSigDB web service (https://www.gsea-msigdb.org/gsea/msigdb/index.jsp) and retaining the top 100 solutions with adjusted p-value < 0.01. In the gene set enrichment analysis of genes with high entropy, we noticed strong enrichment for GO categories associated with cell adhesion (**Supplementary Table 3**). However, this enrichment was due to the presence of a cluster of protocadherin encoding genes within a single chromatin domain. For this reason, we decided to flag and disregard these results as they do not reflect functional similarities among genes in different high-entropy domains.

Figures and Figure legends

Figure 1

a) Main steps of CALDER: Step 1) identifying compartment domains from whole-chromosome contacts (top left); Step 2) deriving sequence-independent hierarchy of compartment domains (right); Step 3) finding nested domains from short-range contacts (bottom left)

b) Maximum number of sub-compartments among which significant differences between mean ChiP-seq intensities of the domains were found for different histone marks (rows) and cell lines (columns).

c) Enrichment of histone marks (rows) in each sub-compartment (columns) of IMR90 cell line. Log_2 fold-changes between the median value within a compartment and the expected median value is color coded. Histone modification names are color coded based on the regulatory element they mark.

d) Enrichment of ChromHMM states (rows) in each sub-compartment (columns) for GM12878. Ratios between the distribution of compartment labels for each ChromHMM state and their expected distribution is color coded and reported.

e) Representative examples of sub-compartments inferred by Calder and SNIPER (colored tracks at the bottom) in two regions of Chr.1. of the GM12878 cell line. ChIP-seq tracks for H3K27me3 (blue), H3K4me3 (green), and H3K27ac (red) are shown in these regions. The bivalent/poised promoter (marked by both H3K4me3 and H3K27me3) of the *ASAP3* gene is marked.

Figure 2

a) ChIP-seq intensity of H3K27ac, H3K4me3, H3K36me3, and H3K27me3 for chromosome 1 compartment domains in GM12878. Each dot is a chromatin domain color coded by ChIP-seq intensity (blue-to-red). Domains were ordered by CALDER-derived domain rank. *r* values are Spearman's correlation coefficients. All correlation tests return p-values < 1E-100.

b-c) LOESS fit lines of ChIP-seq intensity of H3K27ac, H3K4me3, H3K36me3, and H3K27me3 based on domain rank, for 22 chromosomes of GM12878 (**b**), and for chromosome 1 of GM12878, Hela, HMEC, HUVEC, IMR-90, K562 and NHEK (**c**). Lines were normalized to the same maximum Y-axis value of 1.

d) Compartment domain boundaries are classified (Level 1, 2, 3, >3) based on the distance in the hierarchy of the domains separated by the boundary.

e) Fold-change of H3K27ac, H3K4me3, H3K36me3, and H3K27me3 ChIP-seq intensity at boundaries in GM12878, grouped by their level (from left to right) and presence/absence of CTCF (color coded). Nested boundaries correspond to boundaries identified at Step 3 of the algorithm (see panel **Fig. 1a**)

f) Fraction of compartment domain boundaries inferred by Calder and inferred as TAD boundaries by arrowhead (black bars) and TopDom (white bars).

g) H3K27ac fold-changes at domain boundaries exclusively inferred by arrowhead (gray left), TopDom (gray right), and Calder (blue).

h) Visual comparison of compartment domains inferred by Calder (yellow track) and TADs inferred by TopDom (white track) on a Chr. 6 genomic region of the GM12878 cell line. The corner of the TAD identified by TopDom but not by Calder is circled. Domain tracks are overlaid to the Hi-C contact map (left) and the matrix of correlations of observed/expected contact ratios (right), which has been previously shown to reveal chromatin compartments. Sub-compartment annotations and ChIP-seq tracks for H3K27ac (dark red), H3K4me1 (red) and H3K36me3 (orange) are shown on the left.

Figure 3

a) mRNA expression levels (RNA-seq, log-transformed FPKM) of *OSBP* and *CD20* (gene name: *MS4A1*) in the IMR90 cell line and two replicates of the GM12878.

b) Chromatin domain hierarchy built by CALDER for chromosome 11 in IMR90 (bottom) and GM12878 (top). TADs containing OSBP and CD20 are indicated and the 8 sub-compartments are indicated in the hierarchy. The CD20 domain is repositioned from B.2.2 to A.1.1 compartment (arrow).

c) Observed/expected (O/E) contact map of GM12878 (lower triangle) and IMR90 (upper triangle) at Chr.11:58,700-61,000 kb. The *OSBP* and *MS4A1* gene loci are indicated in red.

d-f) Clustering of 127 Hi-C maps based on the sub-compartment annotation of each bin (8-compartment model). Hi-C maps are annotated with their contact density (histogram), bin size for maps analyzed at different bin sizes, and based on whether they were derived from a healthy or cancerous tissue (black boxes) (**e**). Representative clusters and cell lines are highlighted (**f**).

g) Compartment entropy distribution of genomic bins colored by their compartment annotation.

h) Gene ontology enrichment [-log10(q-value)] for genes in low (S < 0.15) and high (S > 0.95) entropy bins

i) Comparison of the number of bins assigned to each compartment (color coded) between H1 embryonic stem cells (X-axis) and H1 lineage differentiation in mesendoderm (left), neural progenitors (center), and trophectoderm (right).

Figure 4

a) Schematic comparison of the comparison between normal and cancer cell lines from breast (red), prostate (blue), and pancreas (purple) tissues.

b) Compartment (color coded) and domain rank (Y-axis) of the *FOXO1*-containing domain in normal and cancer cells.

c) H3K27ac intensity at the *FOXO1* locus for normal and cancer cell lines derived from breast (red), prostate (blue), and pancreatic (purple) tissues.

d-e) *FOXO1* mRNA expression level (log2-transformed FPKM normalized RNA-seq reads) in either the cell lines with matching Hi-C data (**d**), or in normal and cancer samples profiled by The Cancer Genome Atlas (TCGA) (**e**). Each data point represents a replicate in (**d**) and a sample in (**e**).

f) Schematic representation of inter-domain interactions (dashed lines) between the *FOXO1*-containing compartment domain (yellow) and regions that do not change compartment in normal and cancer cells (gray). The *FOXO1*-domain is repositioned in cancer cells.

g) Distance normalized contact values between *FOXO1* and regions that do not change compartment in the compared breast (left), prostate (center), and pancreatic (right) cell lines.

Supplementary Figure Legends

Supplementary Figure 1

a) *21 Feuilles Blanches* (1953), Alexandre Calder (1898-1976)

b) Comparison of A and B compartments called by Calder (yellow), inferred by principal component analysis (Liebermann et al. 2009) (red), and determined by clustering inter-chromosomal interactions either as reported in (Rao et al. 2004) or using SNIPER (blue). Values correspond to the percentage of genomic bins assigned to the same A or B compartment by the different methods.

Supplementary Figure 2

a) Clustering of sub-compartment profiles inferred from 32 Hi-C contact maps corresponding to 8 cell lines and 4 normalization methods for each cell line map. Clustering was based on Pearson's correlation coefficients between each pair of sub-compartment profiles (8 sub-compartments).

b) Enrichment of histone marks (rows) in each sub-compartment (columns). $\log_2$ fold-changes between the median value within a compartment and the overall median value is color coded and reported for 5 cell lines (cell line name reported on the top of the heatmap).

Supplementary Figure 3

a) Correlation between histone mark intensities and sub-compartment assignment inferred by Calder (C, red points), Adaptive K-means (K, dark blue points), and SNIPER (S, light blue points). Boxplots relative to each histone mark are arranged by columns and to each cell line by row.

b) Enrichment (observed/expected) of overlaps between sub-compartments called by SNIPER (columns) and CALDER (rows) in 6 cell lines (indicated in the label).

Supplementary Figure 4

a) Minimum and maximum sub-compartment size (measured as % of the genome) identified by Calder (top) and SNIPER (bottom) across 38 cell lines. Zoom-in pie charts of sub-compartment distribution are shown for the cell lines where SNIPER inferred most (IMR90) and least (THP1-macrophages) balanced sub-compartments.

b) Inter-chromosomal Hi-C contact map (Chr. 2 – Chr. 3) for the renal cancer cell line Caki-2. The dark red corner in the bottom-right area of the map indicates the presence of a chromosomal translocation with a breakpoint corresponding to the vertex of the dark right area (circled in

black). Sub-compartment called by CALDER and SNIPER and expression values (capped at $\log_2$FPKM=5) of genes in these chromosomes are reported on top and left side of the map.

Supplementary Figure 5

a) Distribution of expression contact enrichment (observed/expected) among compartment domains (Y-axis) based on their distance in the compartment hierarchy inferred by Calder (X-axis)

b) Fraction of compartment boundaries identified by both Calder and either one of the two studies referenced in the figure. The fraction is computed with respect to the total number of boundaries inferred by Calder (blue) or either of the referenced studies (red).

c) Histone mark intensity fold-change for 4 histone marks (labelled at the top of each boxplot) observed at Calder inferred boundaries and expected from the background distribution. P-values are computed by tow-tailed Wilcoxon test.

d) CTCF, RAD21 and SMC3 ChIP-seq peak signals (average number of peaks in 5-kb intervals) for boundaries inferred by Calder at different levels.

e) CTCF, RAD21 and SMC3 ChIP-seq peak signals (average number of peaks in 5-kb intervals) for boundaries inferred by both TopDom and Calder (top left), or arrowhead and Calder (bottom left), or exclusively by one of these tools.

f) Change in number of compartment boundaries (stratified by level) inferred by Calder before and after CTCF depletion (left) and depletion of the cohesin loading factor Nipbl (right).

Supplementary Figure 6

a) Distribution of expression differences among gene pairs (Y-axis) based on the distance of their corresponding compartment domains (X-axis)

b) TAD hierarchy inferred by TADpole for IMR90 (left) and GM12878 (right) cell lines. The TADs containing *MS4A1* (a.k.a. *CD20*) and *OSBP* are highlighted.

Supplementary Figure 7

a) Median contact map density across 22 chromosomes (Y-axis) of 114 Hi-C experiments (X-axis). The corresponding total number of contacts are shown for GM12878, KBM7, GM18516, BT474 and MCF-7.

b) Clustering of 127 Hi-C maps based on whole-genome bin compartment annotation. Hi-C dataset labels are shown for all Hi-C dataset including normal (black font) and cancer (blue font) cell lines.

c) Compartment annotation of the *MS4A1* (CD20) locus for all Hi-C datasets clustered as in panel (b) and as in Fig. 3a.

Supplementary Figure 8

a) Clustering of 127 Hi-C maps based on bin sub-compartment annotations inferred by Calder.

b) Clustering of 127 Hi-C maps based on the first principal component values of the observed/expected contact matrix for each bin.

c) Clustering of 127 Hi-C maps based on the sign of the first principal component values of the observed/expected contact matrix for each bin.

Hi-C dataset labels are shown for all Hi-C dataset including normal (black font) and cancer (blue font) cell lines.
d) Clustering of Hi-C maps derived from stem-like cell lines (blue cluster) and differentiated tissues (brown cluster) based on sub-compartment annotations of genomic regions found in the same A (or B) compartment across all cell lines.

Supplementary Figure 9
a) Density distribution of the observed chromatin entropy (light blue) and chromatin entropy after randomization of bin compartment labels (yellow).
b) Density distribution of chromatin entropy in cancer (light blue) and normal (yellow) cell lines.
c) Percentage of bins (color coded in heatmap) that had different compartment in at least one cell line among the 127 analyzed (X-axis) within each compartment (Y-axis).
d) mRNA expression change (log2 fold-change of FPKM normalized RNA-seq reads) of genes having at least 4 compartment difference of between normal and cancer cells derived from breast (left), prostate (center), and pancreatic (right) tissues.

Supplementary Figure 10
Schematic representation of the CALDER algorithm (detailed description in **Methods**).

Supplementary Tables and Data

Supplementary Table 1: Complete list of data sources
IDs and links to Hi-C, ChIP-seq, and RNA-seq datasets used in this study

Supplementary Table 2: Calder vs. SNIPER detailed comparison
Percentage of the genome assigned to each sub-compartment by Calder and SNIPER for the 38 Hi-C datasets in Figure 1e

Supplementary Table 3: Gene set enrichment analysis
Gene set enrichment analysis (mSigDB) results for high entropy ($S>0.95$) and low entropy ($S<0.15$) genes.

Supplementary Table 4: Repositioning events in cancer
Repositioned compartment domains between normal and cancer cell lines derived from breast, prostate, and pancreatic tissue samples.

Supplementary Data 1
Complete domain hierarchies inferred by CALDER from 127 Hi-C contact maps

Supplementary Data 2
Correlation between sub-compartment calls and gene expression for SNIPER and CALDER.

Supplementary Data 3

Matrix of sub-compartment domain assignments for 127 Hi-C contact maps used for the clustering analysis in Figure 3.

References

1. Bonev, B. & Cavalli, G. Organization and function of the 3D genome. *Nat Rev Genet* **17**, 661–678 (2016).
2. Rao, S. S. P. *et al.* A 3D map of the human genome at kilobase resolution reveals principles of chromatin looping. *Cell* **159**, 1665–1680 (2014).
3. Sexton, T. *et al.* Three-Dimensional Folding and Functional Organization Principles of the Drosophila Genome. *Cell* **148**, 458–472 (2012).
4. Lieberman-Aiden, E. *et al.* Comprehensive mapping of long-range interactions reveals folding principles of the human genome. *Science* **326**, 289–293 (2009).
5. Dixon, J. R. *et al.* Topological domains in mammalian genomes identified by analysis of chromatin interactions. *Nature* **485**, 376–380 (2012).
6. Nora, E. P. *et al.* Spatial partitioning of the regulatory landscape of the X-inactivation center. *Nature* **485**, 381–385 (2012).
7. Nuebler, J., Fudenberg, G., Imakaev, M., Abdennur, N. & Mirny, L. A. Chromatin organization by an interplay of loop extrusion and compartmental segregation. *PNAS* **115**, E6697–E6706 (2018).
8. Sanborn, A. L. *et al.* Chromatin extrusion explains key features of loop and domain formation in wild-type and engineered genomes. *Proc. Natl. Acad. Sci. U.S.A.* **112**, E6456-6465 (2015).
9. Nora, E. P. *et al.* Targeted Degradation of CTCF Decouples Local Insulation of Chromosome Domains from Genomic Compartmentalization. *Cell* **169**, 930-944.e22 (2017).
10. Rao, S. S. P. *et al.* Cohesin Loss Eliminates All Loop Domains. *Cell* **171**, 305-320.e24 (2017).
11. Schwarzer, W. *et al.* Two independent modes of chromatin organization revealed by cohesin removal. *Nature* **551**, 51–56 (2017).
12. Falk, M. *et al.* Heterochromatin drives compartmentalization of inverted and conventional nuclei. *Nature* **570**, 395–399 (2019).
13. Beagan, J. A. & Phillips-Cremins, J. E. On the existence and functionality of topologically associating domains. *Nature Genetics* **52**, 8–16 (2020).
14. Dixon, J. R. *et al.* Chromatin architecture reorganization during stem cell differentiation. *Nature* **518**, 331–336 (2015).
15. Kundaje, A. *et al.* Integrative analysis of 111 reference human epigenomes. *Nature* **518**, 317–330 (2015).
16. Yaffe, E. & Tanay, A. Probabilistic modeling of Hi-C contact maps eliminates systematic biases to characterize global chromosomal architecture. *Nature Genetics* **43**, 1059–1065 (2011).
17. Johnstone, S. E. *et al.* Large-Scale Topological Changes Restrain Malignant Progression in Colorectal Cancer. *Cell* **182**, 1474-1489.e23 (2020).
18. Xiong, K. & Ma, J. Revealing Hi-C subcompartments by imputing inter-chromosomal chromatin interactions. *Nature Communications* **10**, 5069 (2019).

19. Soler-Vila, P., Cuscó, P., Farabella, I., Di Stefano, M. & Marti-Renom, M. A. Hierarchical chromatin organization detected by TADpole. *Nucleic Acids Res* **48**, e39–e39 (2020).
20. An, L. *et al.* OnTAD: hierarchical domain structure reveals the divergence of activity among TADs and boundaries. *Genome Biology* **20**, 282 (2019).
21. Norton, H. K. *et al.* Detecting hierarchical genome folding with network modularity. *Nat. Methods* **15**, 119–122 (2018).
22. Ernst, J. & Kellis, M. Large-scale imputation of epigenomic datasets for systematic annotation of diverse human tissues. *Nature Biotechnology* **33**, 364–376 (2015).
23. Ashoor, H. *et al.* Graph embedding and unsupervised learning predict genomic sub-compartments from HiC chromatin interaction data. *Nature Communications* **11**, 1173 (2020).
24. Zufferey, M., Tavernari, D., Oricchio, E. & Ciriello, G. Comparison of computational methods for the identification of topologically associating domains. *Genome Biology* **19**, 217 (2018).
25. Rowley, M. J. *et al.* Evolutionarily Conserved Principles Predict 3D Chromatin Organization. *Molecular Cell* **67**, 837-852.e7 (2017).
26. Shin, H. *et al.* TopDom: an efficient and deterministic method for identifying topological domains in genomes. *Nucleic Acids Res.* **44**, e70 (2016).
27. Eijkelenboom, A. & Burgering, B. M. T. FOXOs: signalling integrators for homeostasis maintenance. *Nature Reviews Molecular Cell Biology* **14**, 83–97 (2013).
28. Fu, Z. & Tindall, D. J. FOXOs, cancer and regulation of apoptosis. *Oncogene* **27**, 2312–2319 (2008).
29. Barretina, J. *et al.* The Cancer Cell Line Encyclopedia enables predictive modelling of anticancer drug sensitivity. *Nature* **483**, 603–607 (2012).
30. Sanchez-Vega, F. *et al.* Oncogenic Signaling Pathways in The Cancer Genome Atlas. *Cell* **173**, 321–337 (2018).
31. Cerami, E. *et al.* The cBio cancer genomics portal: an open platform for exploring multidimensional cancer genomics data. *Cancer Discov* **2**, 401–404 (2012).
32. Durand, N. C. *et al.* Juicer Provides a One-Click System for Analyzing Loop-Resolution Hi-C Experiments. *Cell Syst* **3**, 95–98 (2016).
33. Langmead, B. & Salzberg, S. L. Fast gapped-read alignment with Bowtie 2. *Nat. Methods* **9**, 357–359 (2012).
34. Zhang, Y. *et al.* Model-based analysis of ChIP-Seq (MACS). *Genome Biol.* **9**, R137 (2008).
35. Li, B. & Dewey, C. N. RSEM: accurate transcript quantification from RNA-Seq data with or without a reference genome. *BMC Bioinformatics* **12**, 323 (2011).
36. Dobin, A. *et al.* STAR: ultrafast universal RNA-seq aligner. *Bioinformatics* **29**, 15–21 (2013).
37. Imakaev, M. *et al.* Iterative Correction of Hi-C Data Reveals Hallmarks of Chromosome Organization. *Nat Methods* **9**, 999–1003 (2012).

Figure 1

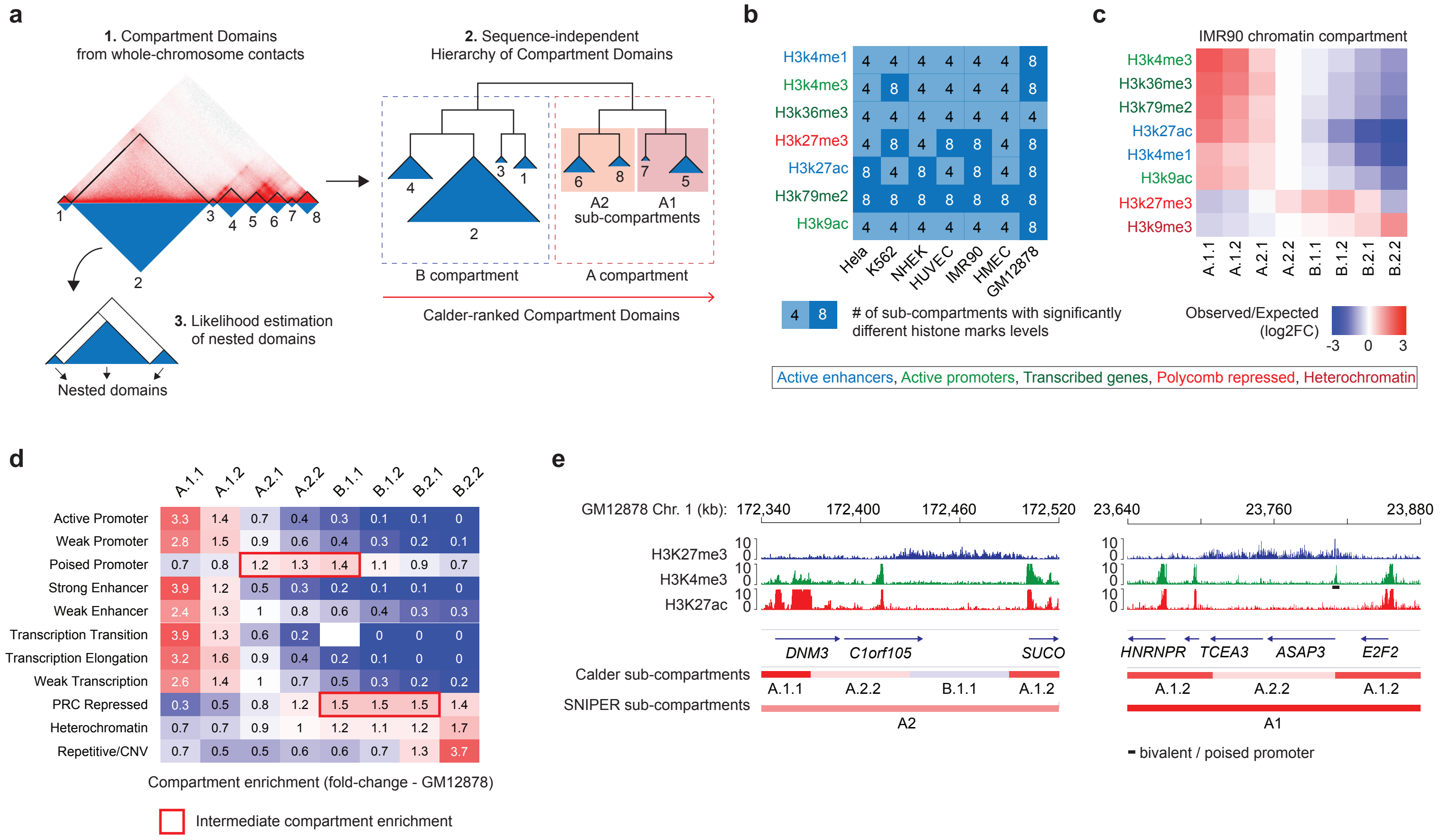
a
1. Compartment Domains from whole-chromosome contacts
2. Sequence-independent Hierarchy of Compartment Domains
3. Likelihood estimation of nested domains
Nested domains
B compartment
A compartment
A2 A1 sub-compartments
Calder-ranked Compartment Domains
b
H3k4me1 4 4 4 4 4 4 8
H3k4me3 4 8 4 4 4 4 8
H3k36me3 4 4 4 4 4 4 4
H3k27me3 4 8 4 8 8 4 8
H3k27ac 8 4 8 4 8 4 8
H3k79me2 8 8 8 8 8 8 8
H3k9ac 4 4 4 4 4 4 8
Hela K562 NHEK HUVEC IMR90 HMEC GM12878
# of sub-compartments with significantly different histone marks levels
c
IMR90 chromatin compartment
H3k4me3 H3k36me3 H3k79me2 H3k27ac H3k4me1 H3k9ac H3k27me3 H3k9me3
A.1.1 A.1.2 A.2.1 A.2.2 B.1.1 B.1.2 B.2.1 B.2.2
Observed/Expected (log2FC) -3 0 3
Active enhancers, Active promoters, Transcribed genes, Polycomb repressed, Heterochromatin
d
A.1.1 A.1.2 A.2.1 A.2.2 B.1.1 B.1.2 B.2.1 B.2.2
Active Promoter 3.3 1.4 0.7 0.4 0.3 0.1 0.1 0
Weak Promoter 2.8 1.5 0.9 0.6 0.4 0.3 0.2 0.1
Poised Promoter 0.7 0.8 1.2 1.3 1.4 1.1 0.9 0.7
Strong Enhancer 3.9 1.2 0.5 0.3 0.2 0.1 0.1 0
Weak Enhancer 2.4 1.3 1 0.8 0.6 0.4 0.3 0.3
Transcription Transition 3.9 1.3 0.6 0.2 0 0 0
Transcription Elongation 3.2 1.6 0.9 0.4 0.2 0.1 0 0
Weak Transcription 2.6 1.4 1 0.7 0.5 0.3 0.2 0.2
PRC Repressed 0.3 0.5 0.8 1.2 1.5 1.5 1.5 1.4
Heterochromatin 0.7 0.7 0.9 1 1.2 1.1 1.2 1.7
Repetitive/CNV 0.7 0.5 0.5 0.6 0.6 0.7 1.3 3.7
Compartment enrichment (fold-change - GM12878)
Intermediate compartment enrichment
e
GM12878 Chr. 1 (kb): 172,340 172,400 172,460 172,520
23,640 23,760 23,880
H3K27me3
H3K4me3
H3K27ac
DNM3 C1orf105 SUCO
HNRNPR TCEA3 ASAP3 E2F2
Calder sub-compartments
A.1.1 A.2.2 B.1.1 A.1.2
A.1.2 A.2.2 A.1.2
SNIPER sub-compartments
A2
A1
bivalent / poised promoter

Figure 2

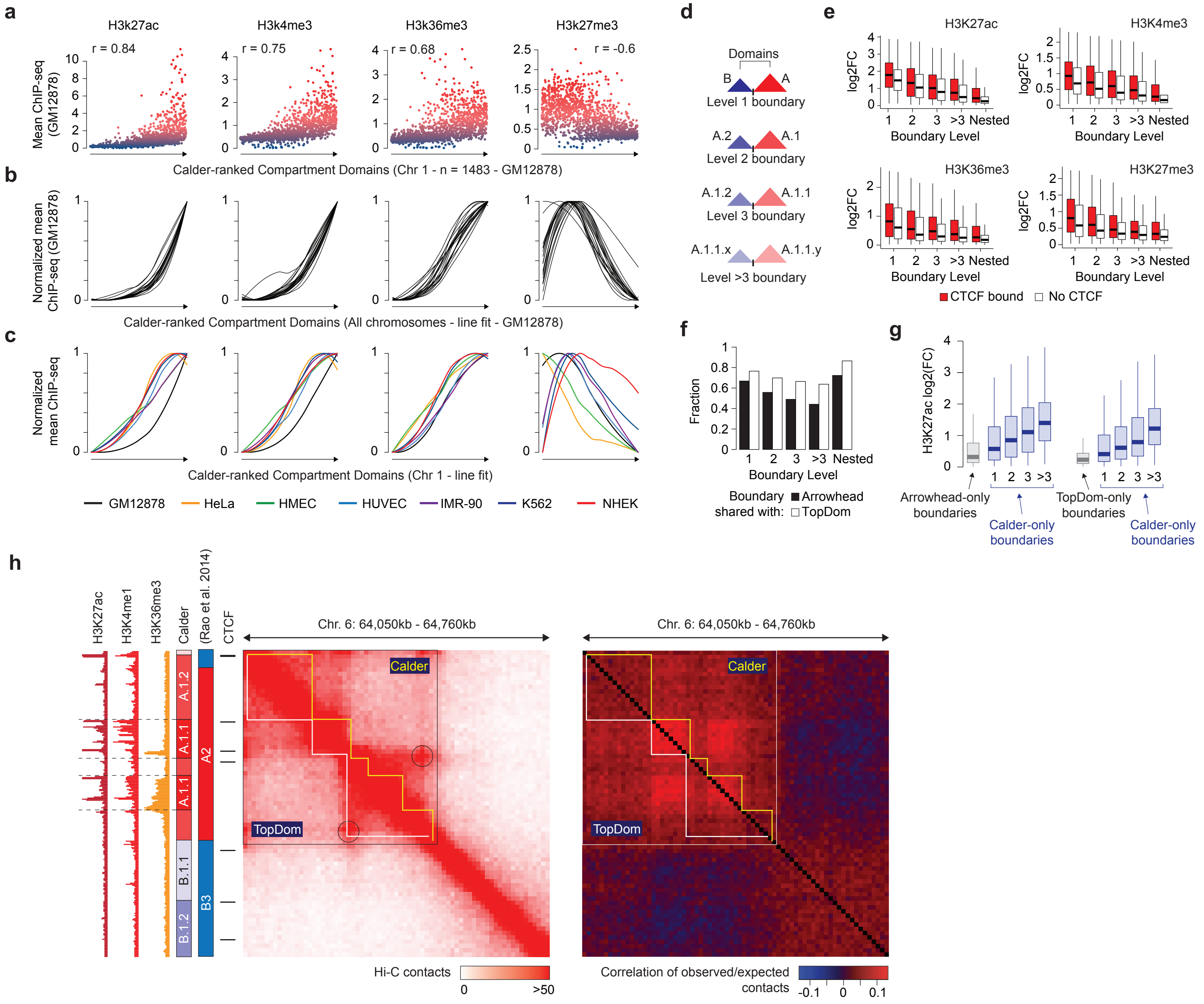
a
H3k27ac
H3k4me3
H3k36me3
H3k27me3
r = 0.84
r = 0.75
r = 0.68
r = -0.6
Mean ChIP-seq (GM12878)
Calder-ranked Compartment Domains (Chr 1 - n = 1483 - GM12878)
b
Normalized mean ChIP-seq (GM12878)
Calder-ranked Compartment Domains (All chromosomes - line fit - GM12878)
c
Normalized mean ChIP-seq
Calder-ranked Compartment Domains (Chr 1 - line fit)
GM12878
HeLa
HMEC
HUVEC
IMR-90
K562
NHEK
d
Domains
B
A
Level 1 boundary
A.2
A.1
Level 2 boundary
A.1.2
A.1.1
Level 3 boundary
A.1.1.x
A.1.1.y
Level >3 boundary
e
H3K27ac
H3K4me3
H3K36me3
H3K27me3
log2FC
Boundary Level
1 2 3 >3 Nested
CTCF bound
No CTCF
f
Fraction
1 2 3 >3 Nested
Boundary Level
Boundary shared with:
Arrowhead
TopDom
g
H3K27ac log2(FC)
1 2 3 >3
Arrowhead-only boundaries
Calder-only boundaries
TopDom-only boundaries
Calder-only boundaries
h
H3K27ac
H3K4me1
H3K36me3
Calder
(Rao et al. 2014)
CTCF
A.1.2
A.1.1
A.1.1
B.1.1
B.1.2
A2
B3
Chr. 6: 64,050kb - 64,760kb
Calder
TopDom
Hi-C contacts
0
>50
Correlation of observed/expected contacts
-0.1
0
0.1

Figure 3

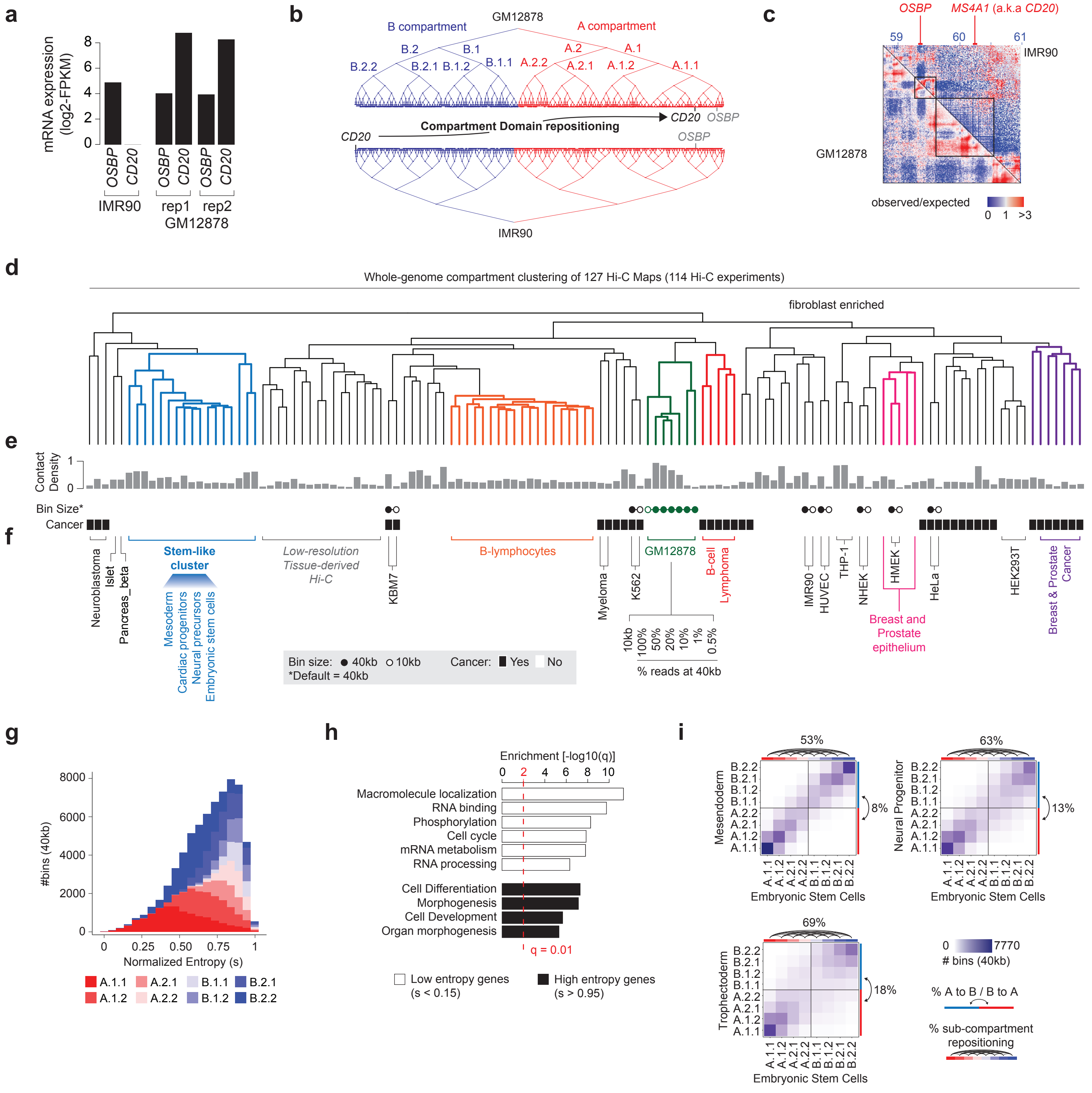
a
mRNA expression (log2-FPKM)
OSBP
CD20
IMR90
rep1
rep2
GM12878
b
GM12878
B compartment
A compartment
B.2
B.1
A.2
A.1
B.2.2
B.2.1
B.1.2
B.1.1
A.2.2
A.2.1
A.1.2
A.1.1
Compartment Domain repositioning
CD20
OSBP
IMR90
c
OSBP
MS4A1 (a.k.a CD20)
59
60
61
IMR90
GM12878
observed/expected
0
1
>3
d
Whole-genome compartment clustering of 127 Hi-C Maps (114 Hi-C experiments)
fibroblast enriched
e
Contact Density
Bin Size*
Cancer
f
Neuroblastoma
Islet
Pancreas_beta
Stem-like cluster
Mesoderm
Cardiac progenitors
Neural precursors
Embryonic stem cells
Low-resolution Tissue-derived Hi-C
KBM7
B-lymphocytes
Myeloma
K562
GM12878
B-cell Lymphoma
10kb
100%
50%
20%
10%
1%
0.5%
% reads at 40kb
IMR90
HUVEC
THP-1
NHEK
HMEK
Breast and Prostate epithelium
HeLa
HEK293T
Breast & Prostate Cancer
Bin size: 40kb 10kb
*Default = 40kb
Cancer: Yes No
g
#bins (40kb)
Normalized Entropy (s)
A.1.1
A.1.2
A.2.1
A.2.2
B.1.1
B.1.2
B.2.1
B.2.2
h
Enrichment [-log10(q)]
Macromolecule localization
RNA binding
Phosphorylation
Cell cycle
mRNA metabolism
RNA processing
Cell Differentiation
Morphogenesis
Cell Development
Organ morphogenesis
q = 0.01
Low entropy genes (s < 0.15)
High entropy genes (s > 0.95)
i
53%
Mesendoderm
8%
Embryonic Stem Cells
63%
Neural Progenitor
13%
Embryonic Stem Cells
69%
Trophectoderm
18%
Embryonic Stem Cells
0
7770
bins (40kb)
% A to B / B to A
% sub-compartment repositioning

Figure 4

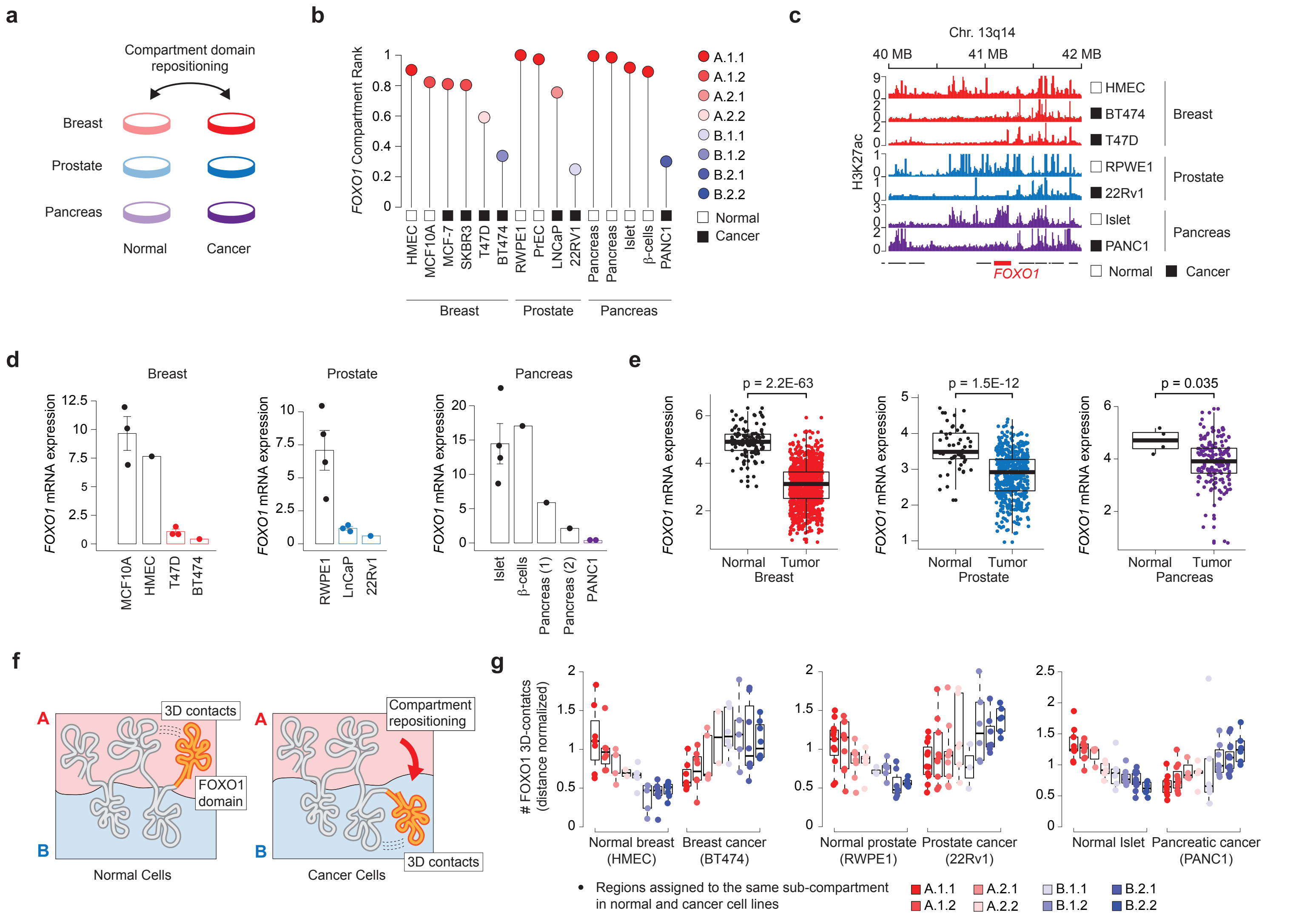
a
Compartment domain repositioning
Breast
Prostate
Pancreas
Normal
Cancer
b
FOXO1 Compartment Rank
HMEC
MCF10A
MCF-7
SKBR3
T47D
BT474
RWPE1
PrEC
LNCaP
22RV1
Pancreas
Pancreas
Islet
β-cells
PANC1
Breast
Prostate
Pancreas
A.1.1
A.1.2
A.2.1
A.2.2
B.1.1
B.1.2
B.2.1
B.2.2
Normal
Cancer
c
Chr. 13q14
40 MB
41 MB
42 MB
H3K27ac
HMEC
BT474
T47D
Breast
RPWE1
22Rv1
Prostate
Islet
PANC1
Pancreas
FOXO1
Normal
Cancer
d
Breast
Prostate
Pancreas
FOXO1 mRNA expression
MCF10A
HMEC
T47D
BT474
RWPE1
LnCaP
22Rv1
Islet
β-cells
Pancreas (1)
Pancreas (2)
PANC1
e
p = 2.2E-63
p = 1.5E-12
p = 0.035
FOXO1 mRNA expression
Normal
Tumor
Breast
Prostate
Pancreas
f
A
B
3D contacts
FOXO1 domain
Normal Cells
Compartment repositioning
3D contacts
Cancer Cells
g
FOXO1 3D-contatcs (distance normalized)
Normal breast (HMEC)
Breast cancer (BT474)
Normal prostate (RWPE1)
Prostate cancer (22Rv1)
Normal Islet
Pancreatic cancer (PANC1)
Regions assigned to the same sub-compartment in normal and cancer cell lines
A.1.1
A.1.2
A.2.1
A.2.2
B.1.1
B.1.2
B.2.1
B.2.2
