# Supplementary material for: Systematic inference and comparison of multi-scale chromatin sub-compartments connects spatial organization to cell phenotypes
Source: Nat Commun. 2021 May 10;12:2439. doi: 10.1038/s41467-021-22666-3 (PMC8110550; doi:10.1038/s41467-021-22666-3)

Supplementary figure 10

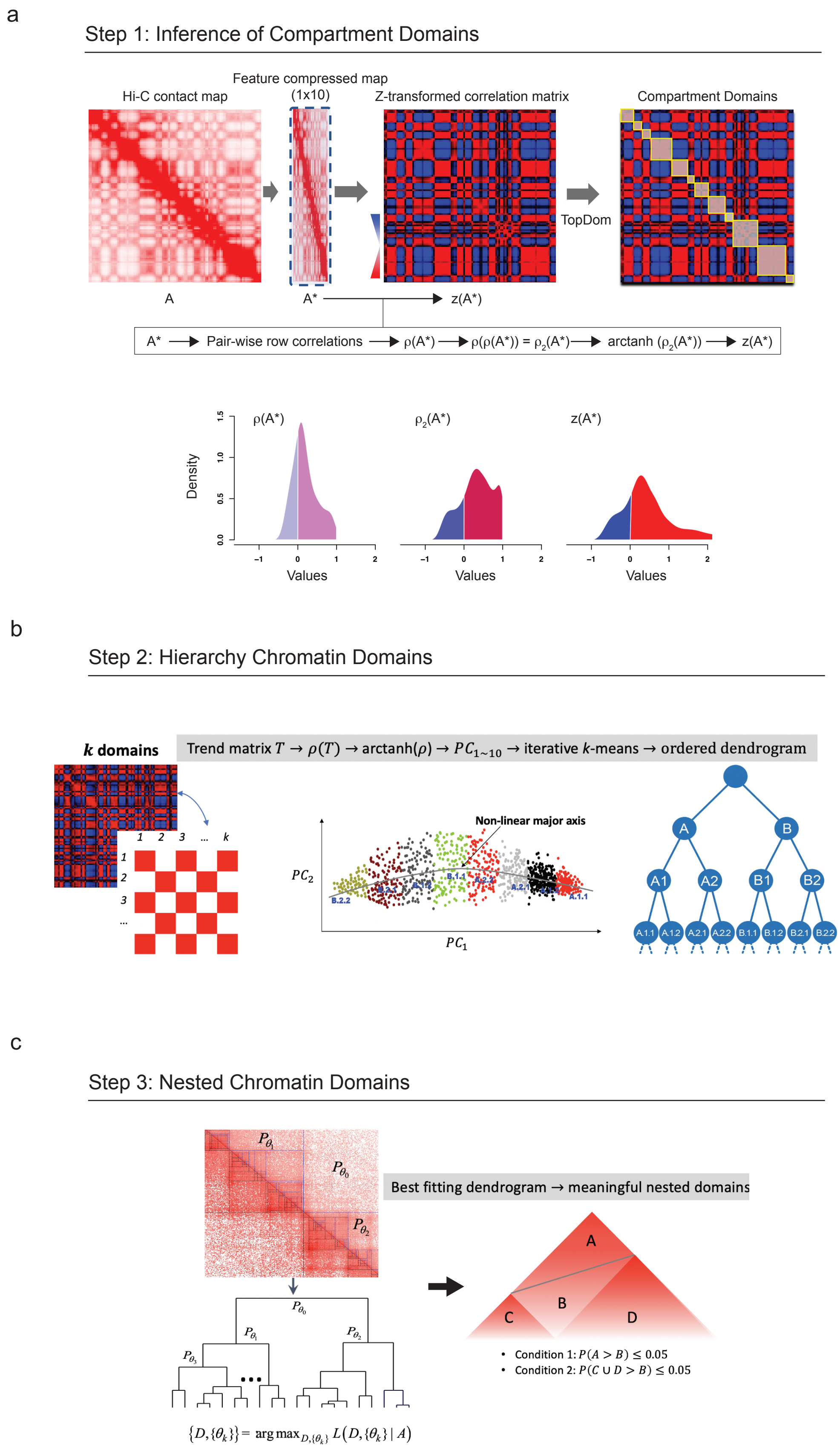

Supplement: Supplementary file 8 — Supplementary Dataset 5 [file 41467_2021_22666_MOESM8_ESM.zip › 291893_0_supp_5211662_qmlr7l.pdf]
