# Supplementary figures and images for: Systematic inference and comparison of multi-scale chromatin sub-compartments connects spatial organization to cell phenotypes
Source: Nat Commun. 2021 May 10;12:2439. doi: 10.1038/s41467-021-22666-3 (PMC8110550; doi:10.1038/s41467-021-22666-3)

Supplementary Figure 1

a

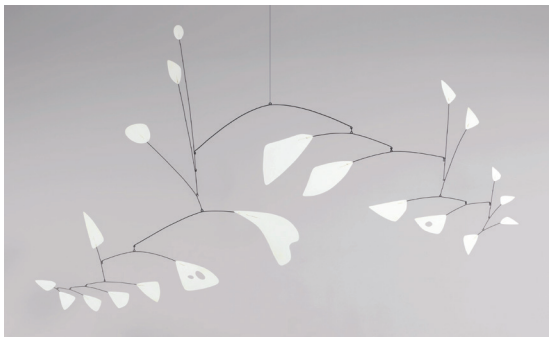

b

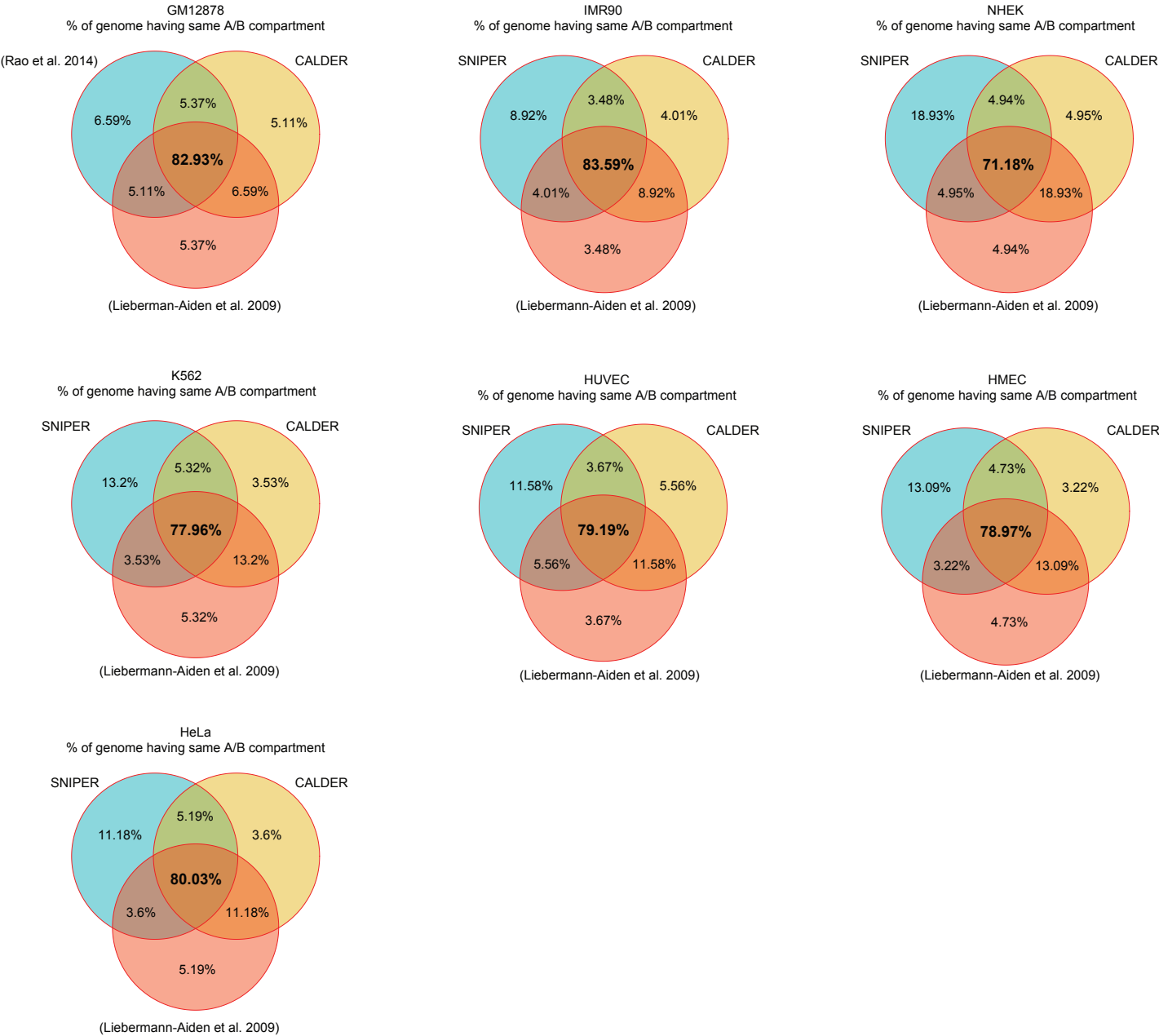

Supplement: Supplementary file 8 — Supplementary Dataset 5 [file 41467_2021_22666_MOESM8_ESM.zip › 291893_0_supp_5211588_qmbnxq.pdf]

Supplementary Figure 2

a

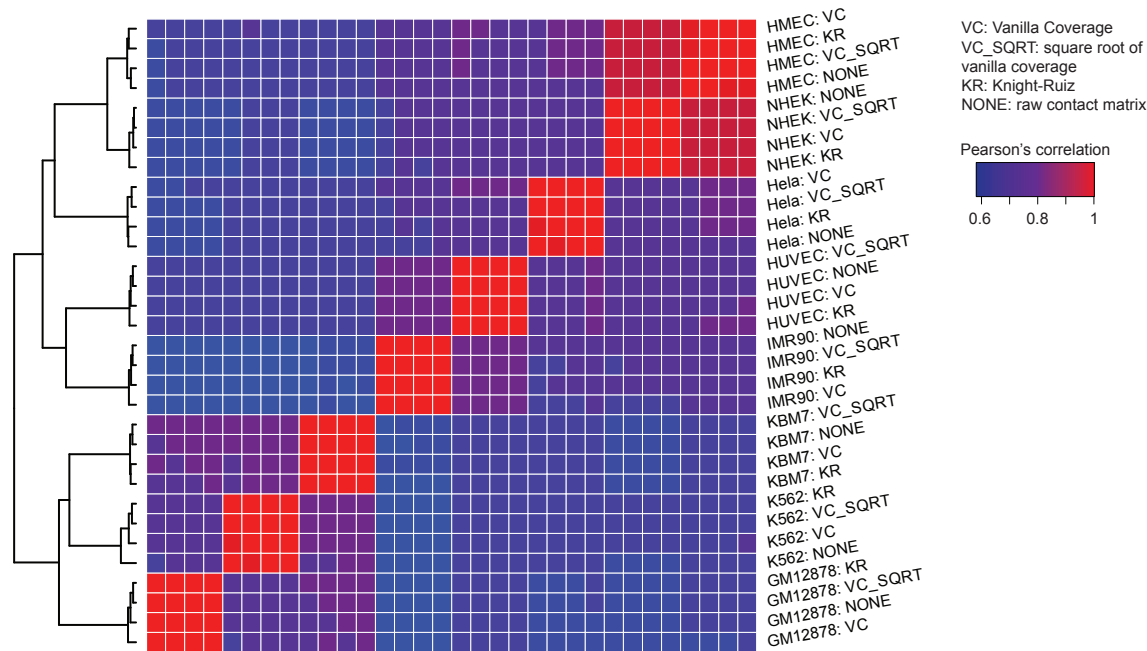

b

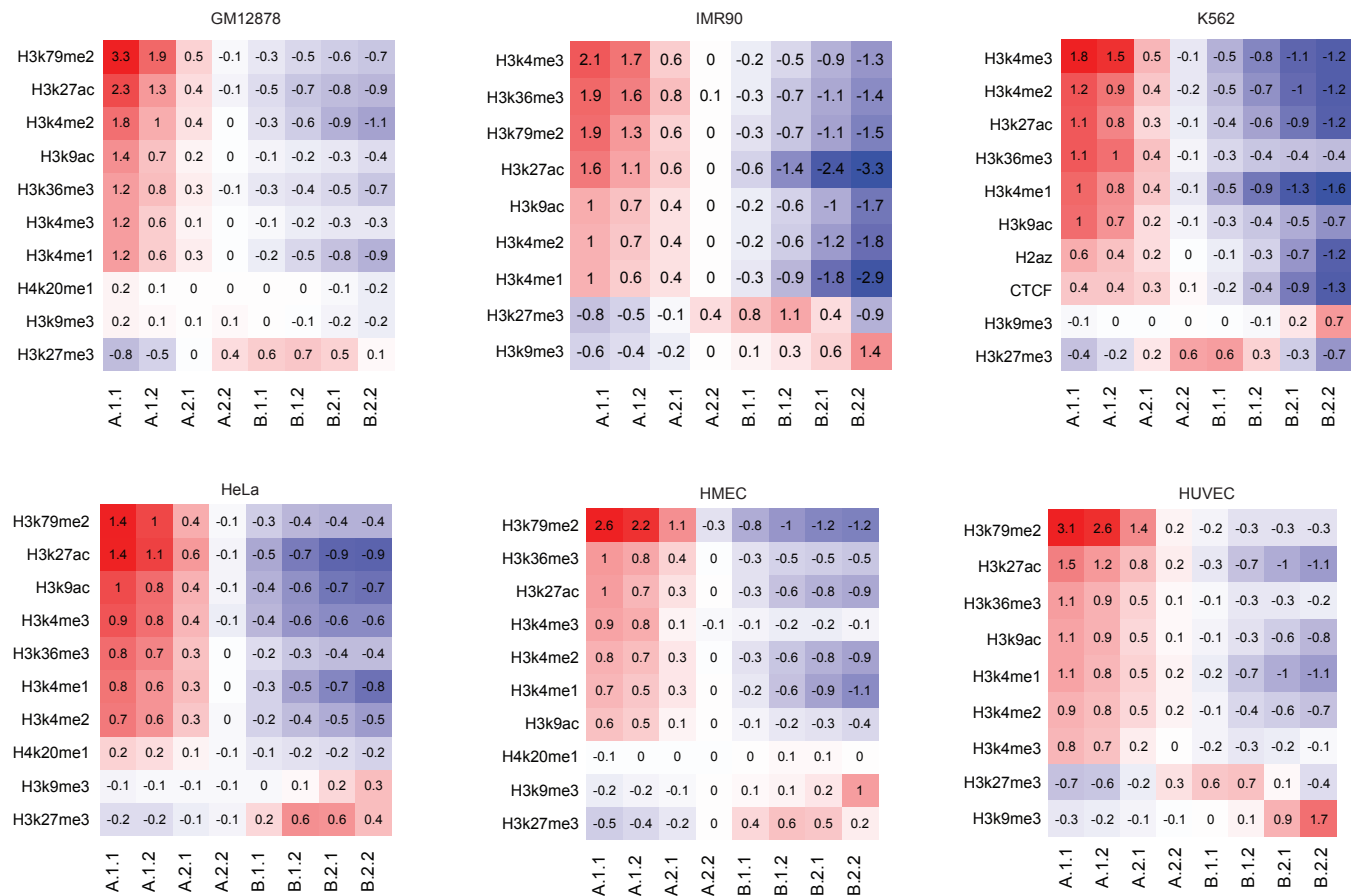

Supplement: Supplementary file 8 — Supplementary Dataset 5 [file 41467_2021_22666_MOESM8_ESM.zip › 291893_0_supp_5211584_qm1nxq.pdf]

Supplementary Figure 3

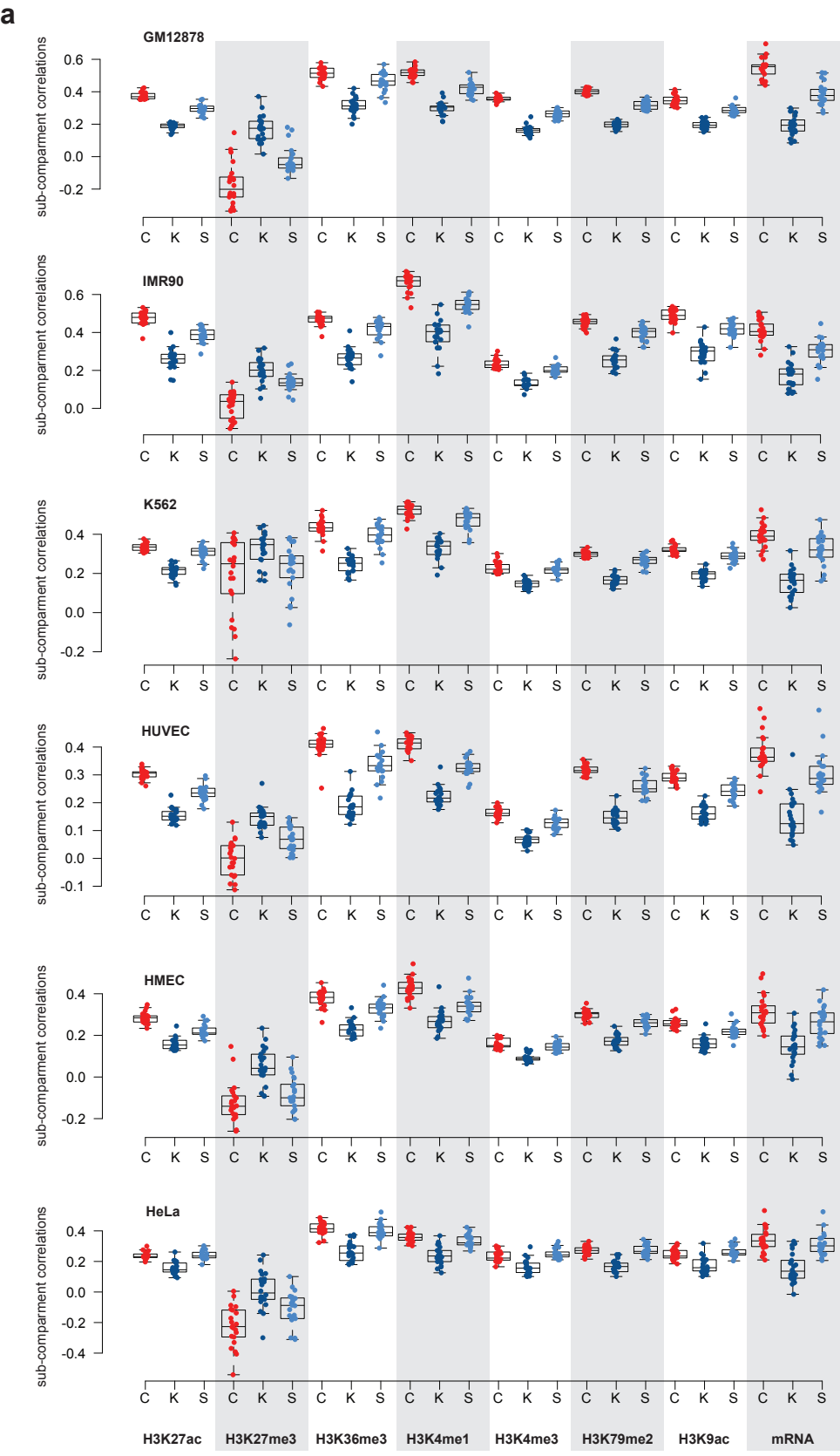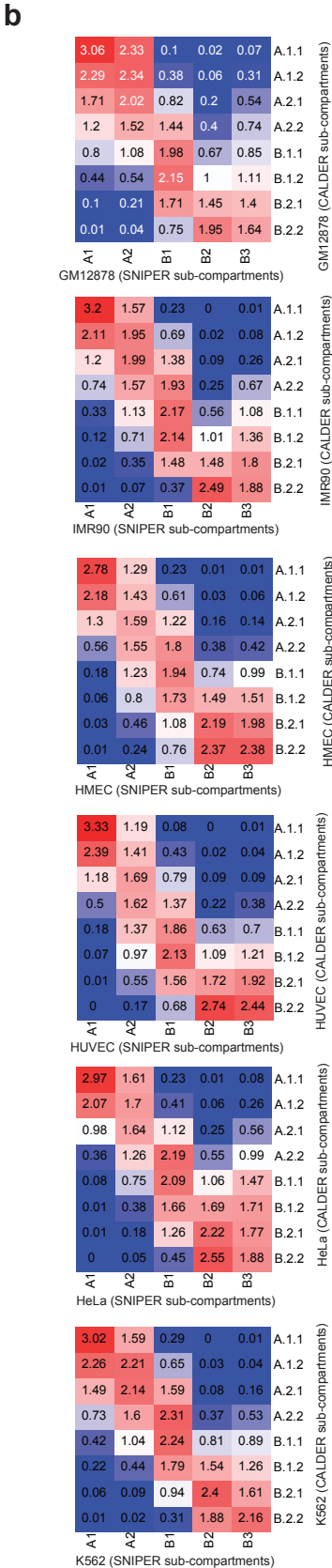

Supplement: Supplementary file 8 — Supplementary Dataset 5 [file 41467_2021_22666_MOESM8_ESM.zip › 291893_0_supp_5211590_qmdnxq.pdf]

Supplementary Figure 4

a

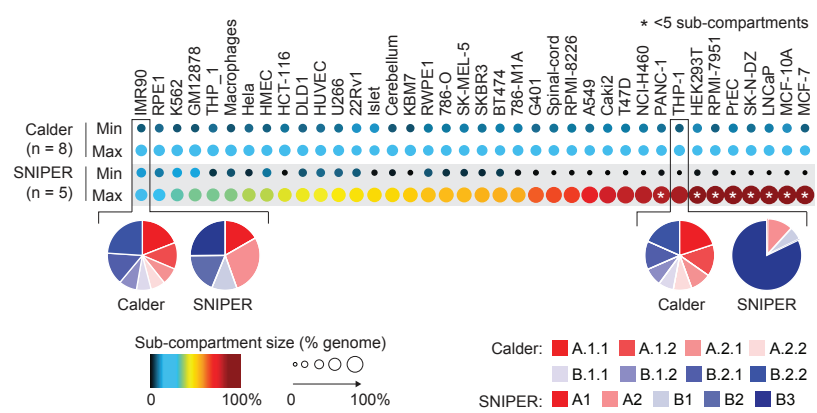

b

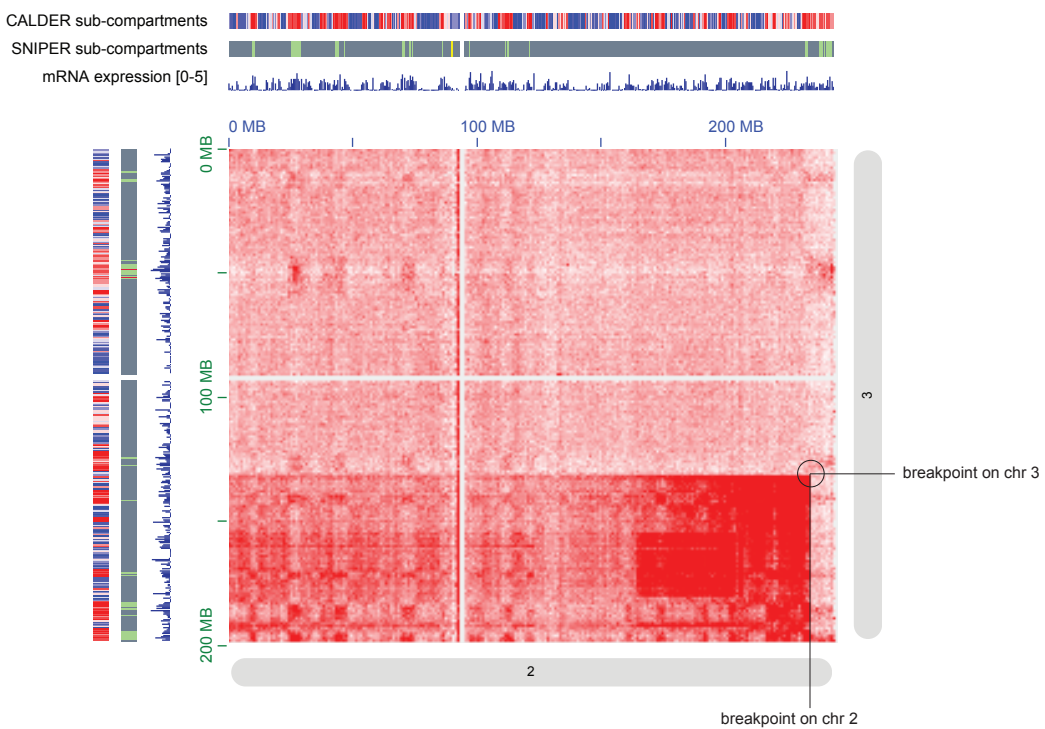

Supplement: Supplementary file 8 — Supplementary Dataset 5 [file 41467_2021_22666_MOESM8_ESM.zip › 291893_0_supp_5211587_qmtnxq.pdf]

## Supplementary Figure 5

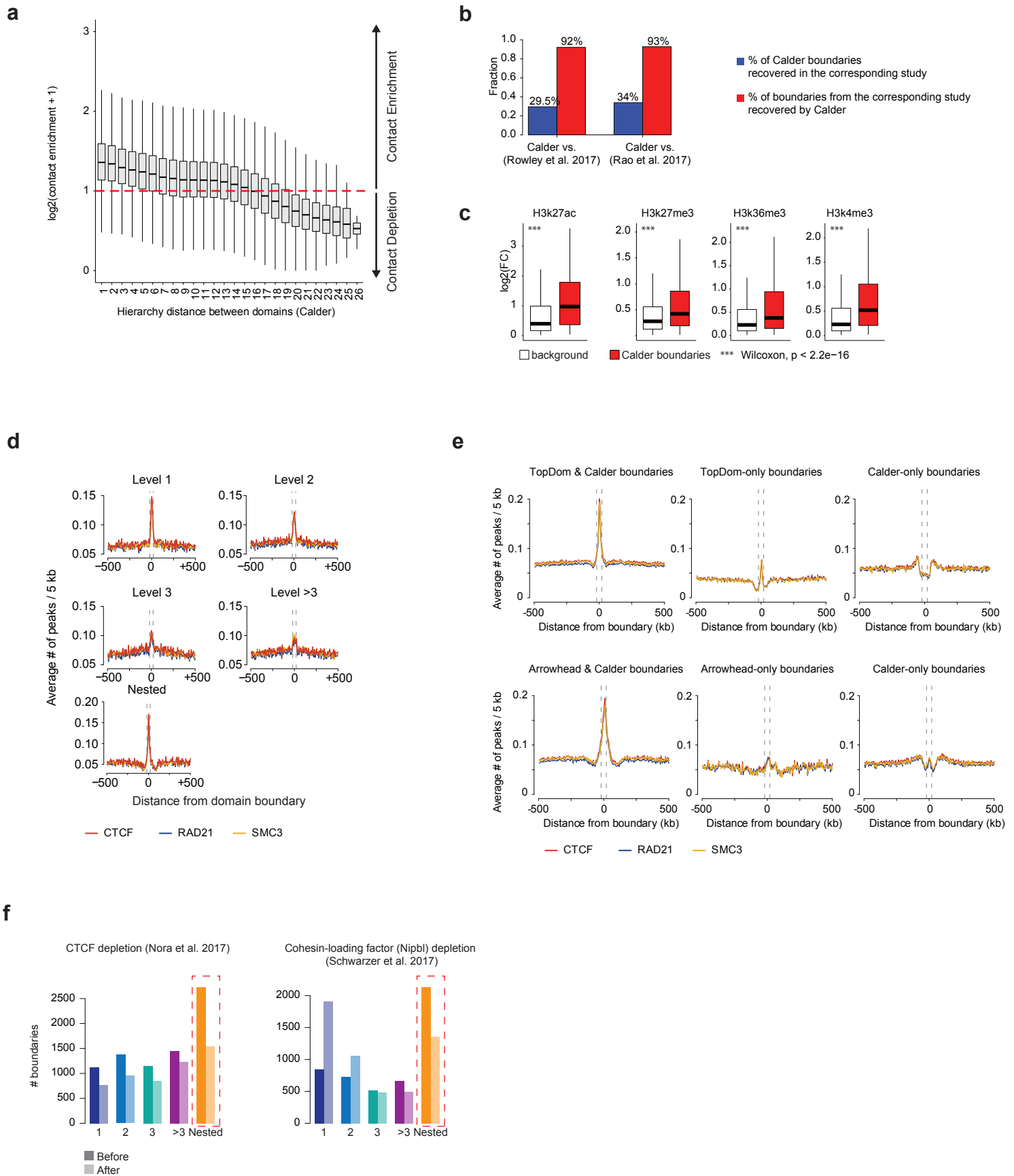

Supplement: Supplementary file 8 — Supplementary Dataset 5 [file 41467_2021_22666_MOESM8_ESM.zip › 291893_0_supp_5211591_qmxnxq.pdf]

## Supplementary Figure 6

**a**

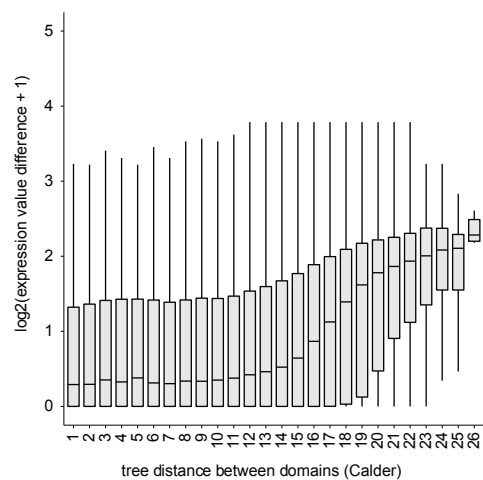

**b**

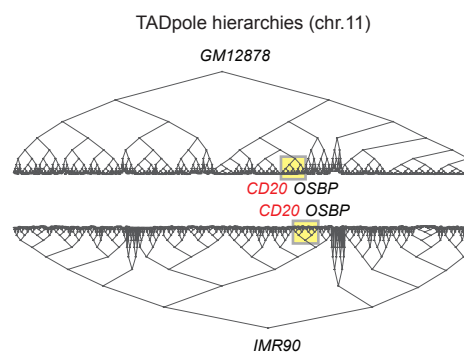

Supplement: Supplementary file 8 — Supplementary Dataset 5 [file 41467_2021_22666_MOESM8_ESM.zip › 291893_0_supp_5211589_qmtnxq.pdf]

Supplementary Figure 7

a

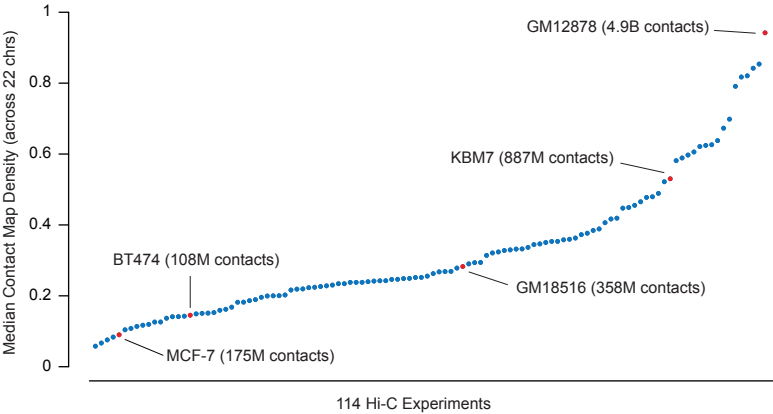

b

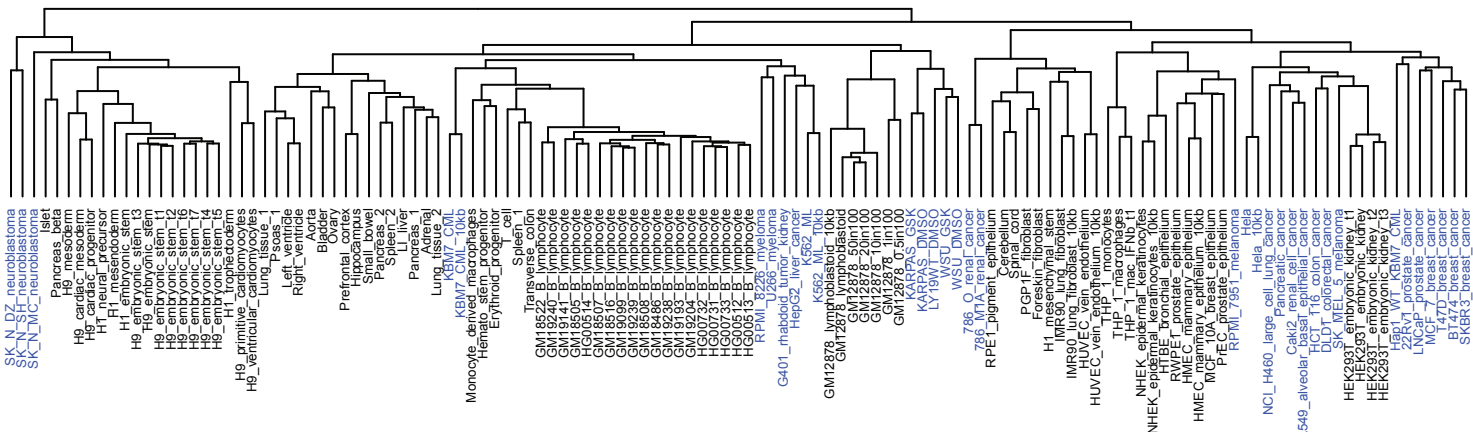

c

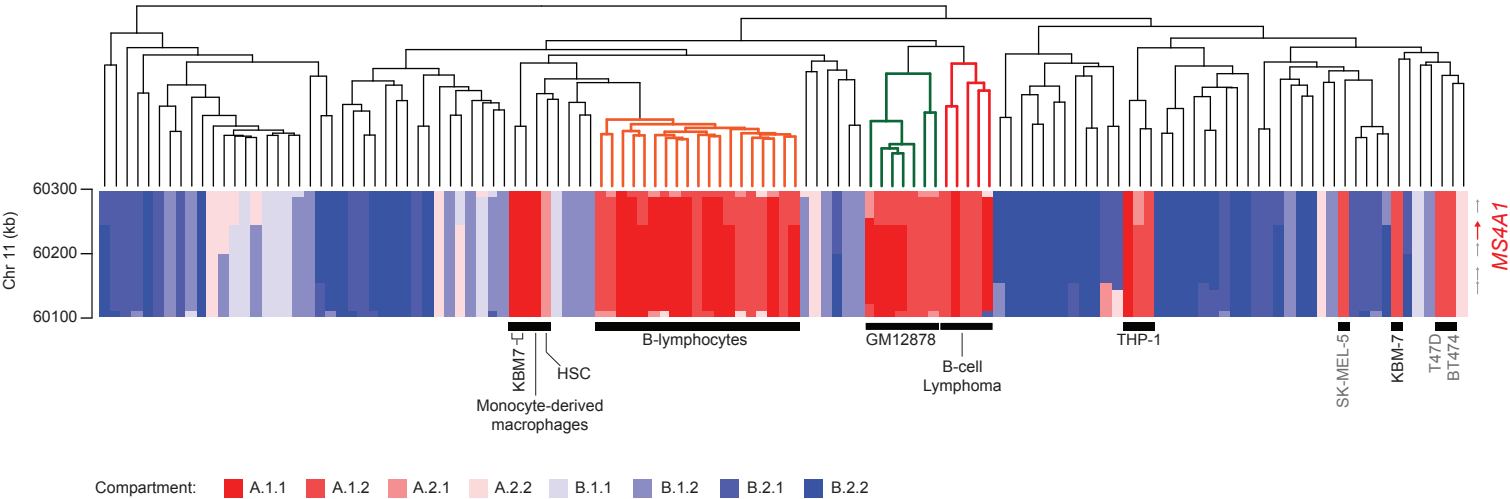

Supplement: Supplementary file 8 — Supplementary Dataset 5 [file 41467_2021_22666_MOESM8_ESM.zip › 291893_0_supp_5211585_qm4nxq.pdf]

Supplementary Figure 9

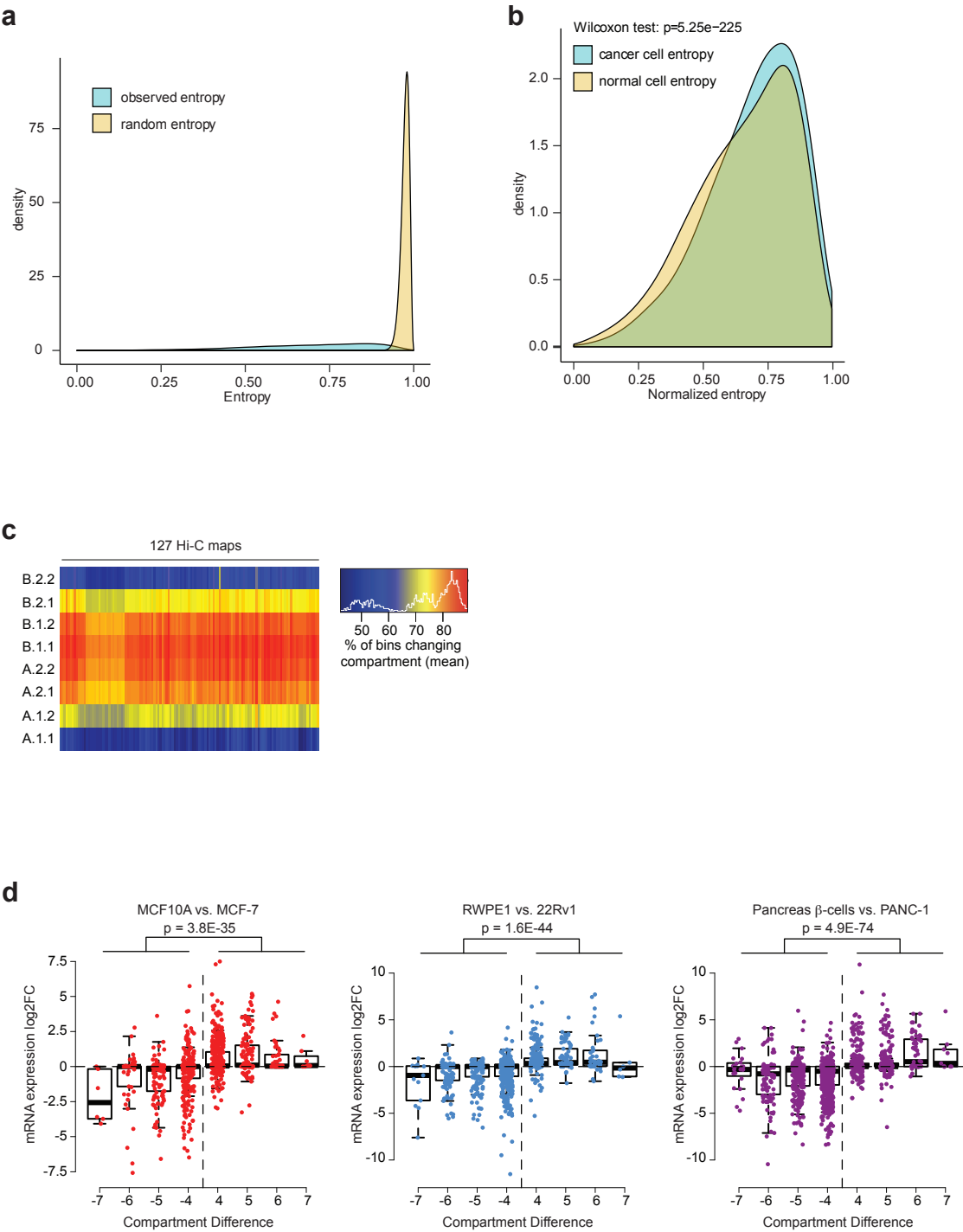

Supplement: Supplementary file 8 — Supplementary Dataset 5 [file 41467_2021_22666_MOESM8_ESM.zip › 291893_0_supp_5211661_qmzzk7.pdf]
