# Supplementary material for: Systematic inference and comparison of multi-scale chromatin sub-compartments connects spatial organization to cell phenotypes
Source: Nat Commun. 2021 May 10;12:2439. doi: 10.1038/s41467-021-22666-3 (PMC8110550; doi:10.1038/s41467-021-22666-3)

Supplementary Figure 8

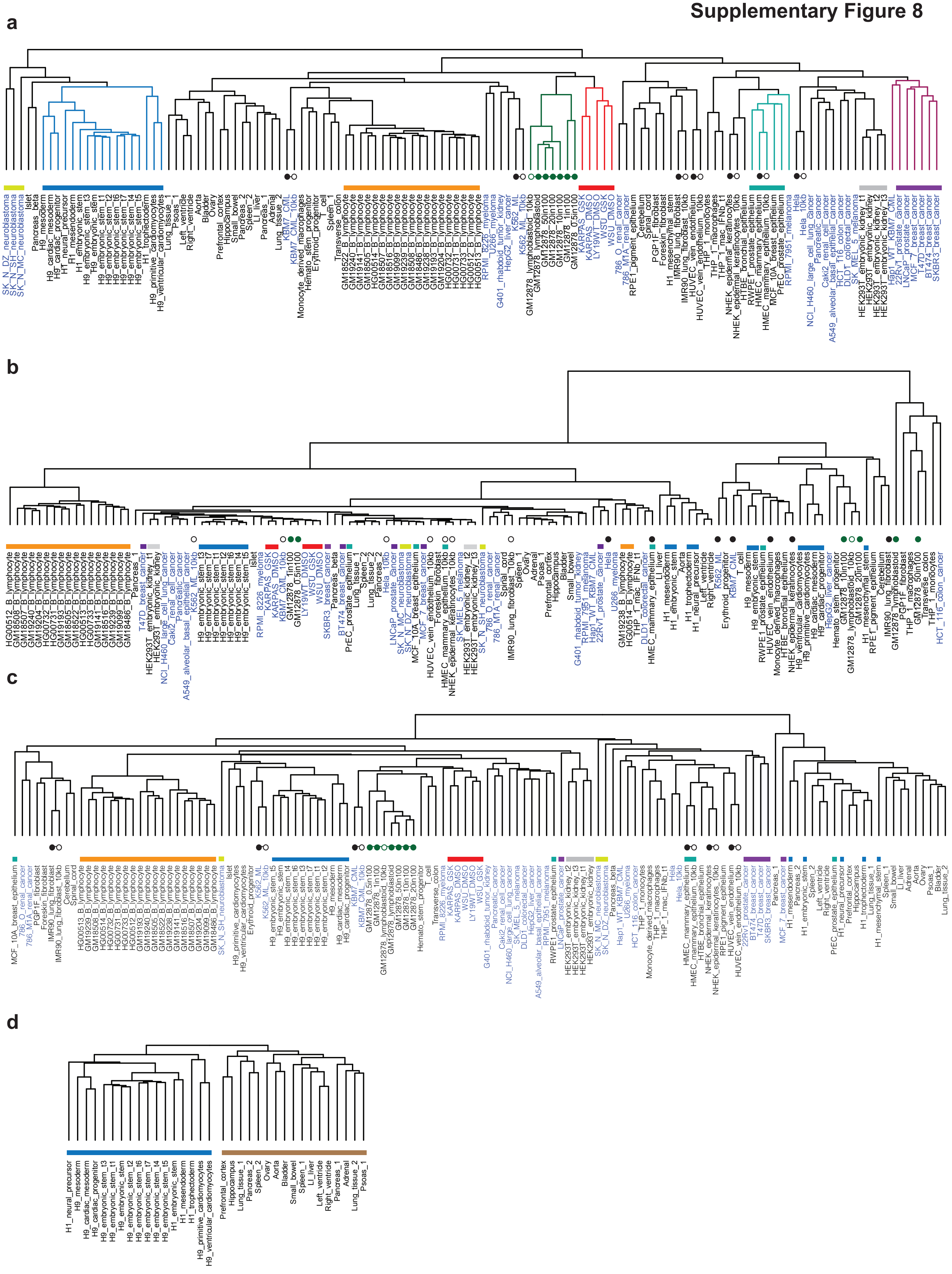

Supplement: Supplementary file 8 — Supplementary Dataset 5 [file 291893_0_supp_5211586_qmtnxq.pdf › 291893_0_supp_5211586_qmtnxq.pdf]
